# Phenotype-Specific Therapeutic Effect of *Rhodiola wallichiana* var. *cholaensis* Combined with Dexamethasone on Experimental Murine Asthma and Its Comprehensive Pharmacological Mechanism

**DOI:** 10.3390/ijms20174216

**Published:** 2019-08-28

**Authors:** Zhiqiang Pang, Nan Ran, Yuze Yuan, Cuizhu Wang, Guoqiang Wang, Hongqiang Lin, Alan Chen-Yu Hsu, Jinping Liu, Fang Wang

**Affiliations:** 1 Department of Pathogen Biology, College of Basic Medical Sciences, Jilin University, Changchun 130021, China; 2 Research Center of Natural Drug, School of Pharmaceutical Sciences, Jilin University, Changchun 130012, China; 3 Faculty of Health and Medicine, School of Medicine and Public Health, The University of Newcastle, Callaghan, NSW 2308, Australia; 4 Key Laboratory of Zoonosis, Ministry of Education, College of Veterinary Medicine, Jilin University, Changchun 130062, China

**Keywords:** asthma, *Rhodiola wallichiana* var. *cholaensis*, dexamethasone, phenotype, multiomics

## Abstract

The heterogeneity of asthma involves complex pathogenesis leading to confusion regarding the choice of therapeutic strategy. In the clinic, asthma is commonly classified as having either eosinophilic asthma (EA) or non-eosinophilic asthma (NEA) phenotypes. Microbiota colonizing in airways has been demonstrated to induce distinct phenotypes of asthma and the resistance to steroids. *Rhodiola wallichiana* var. *cholaensis* (RWC) has the potential to alleviate asthmatic inflammation according to recent studies, but its pharmacological mechanisms remain unclarified. In our study, murine asthmatic phenotypes were established and treated with RWC and/or dexamethasone (DEX). Combined treatment with RWC and DEX could improve spirometry and airway hyperresponsiveness (AHR) in asthmatic phenotypes, alleviate steroid resistance in NEA, and reduce the inflammatory infiltration of the both phenotypes. The combined treatment increased Th1, regulated the imbalance of Th2/Th1, and decreased the related cytokines in EA. As for NEA, the combined treatment reduced Th17 and promoted the accumulation of regulatory T cells (Tregs) in lung. A microbiome study based on 16S rDNA sequencing technique revealed the significantly changed structure of the lower airway microbiota after combined treatment in NEA, with 4 distinct genera and 2 species identified. OPLS-DA models of metabolomics analysis based on UPLC-Q/TOF-MS technique identified 34 differentiated metabolites and 8 perturbed metabolic pathways. A joint multiomics study predicted that the colonized microbiota in airways might be associated with susceptibility of asthma and steroid resistance, which involved systematic and pulmonary metabolic perturbation. In summary, the pharmacological network of RWC included the complicated interaction mechanisms of immune regulation, microbiota change, and metabolic perturbation.

## 1. Introduction

Asthma, as a heterogenous disease, displays different biological phenotypes which are tightly associated with prognosis [1,2]. The phenotypes are often identified based on clinical characteristics or the inflammatory status of asthmatics [3]. Inflammatory phenotyping according to the relative counts of eosinophils and neutrophils in the induced sputum or peripheral blood of patients has been widely used in clinical practices [4,5,6]. Typical asthmatic phenotypes include eosinophilic asthma (EA) and non-eosinophilic asthma (NEA) mainly consisting of neutrophilic asthma [7,8]. As previously described, eosinophilic asthma has a higher risk of exacerbation, but non-eosinophilic asthma usually shows steroid resistance and poorly controlled status [9]. 

Asthma is an airway-restricted disease involving significant mucus inflammation. Recent studies have focused on the pathogenesis of asthma and clarified more immune details of asthmatic phenotypes than ever before. Briefly, the airway inflammation of EA is related to the imbalance of Th1/Th2 [10]. Environmental allergens can stimulate airway epithelia and induce the recruitment of eosinophils and huge release of cytokines, including IL-4, IL-5, and IL-13, which are dominated by Th2 and downregulated by Th1 [11,12]. However, Th17 is activated under the influence of microbial colonization and smoking with the release of IL-17, which could recruit neutrophils into airways [11,13]. Regulatory T cells (Tregs) suppress the inflammation from Th17 with fibrinogen-like protein 2 and play a critical negative regulation on the neutrophilic inflammation [14]. Despite the use of dexamethasone and various monoclonal antibody medicines that have been developed to control Th2-dominated asthmatic inflammation, the neutrophilic phenotype has barely been controlled in the clinic [15,16].

Root aqueous extracts of *Rhodiola wallichiana* var. *cholaensis* (RWC) are a natural plant medicine traditionally used to strengthen myocardial contractility and reduce the myocardial oxygen [17]. However, the extensive pharmacological activities of RWC have rarely been reported. Recently, it was found that the main component of RWC, salidroside, could inhibit ovalbumin (OVA)-induced airway inflammation and alleviate airway hyperresponsiveness (AHR) of murine asthma and reduce the phosphorylation of p38 MAPK, involved a critical signaling pathway related with steroid resistance [18,19,20]. Moreover, a number of signaling pathways related to the pathogenesis of asthma have also been discovered to be regulated by the main chemical components of RWC, salidroside and tyrosol [21,22]. However, the detailed therapeutic effect of RWC on asthma or asthmatic phenotypes have never been evaluated. Dexamethasone (DEX), as the most characteristic therapeutic agent for treating asthma, can systemically reduce inflammation, decrease mucus production, and enhance the effects of β-agonists on asthma [23]. Whether RWC together with DEX can synergistically improve the syndrome of a specific asthma phenotype and contribute to reducing steroid resistance needs investigation.

Systematic biology has focused on the comprehensive details of diseases and made great progress in elucidating the pathogenesis of asthma. Using microbiome techniques, Hilty et al. first observed that Proteobacteria species were increased in the lower airway of asthmatics, especially *Haemophilus* [24]. Some of the specific pathogens that colonized in lower respiratory tracts, like *Haemophilus influenzae*, participate in the regulation of Th1/Th2/Th17 inflammation in asthma [25]. Our previous study has also reported on a significant distinction in microbiota between the two asthma phenotypes [2]. In addition, metabolomics has been used to identify changed metabolic patterns and to further clarify the pathogenesis of asthma. Various metabolites such as amino acids and phospholipids have been identified [26,27]. Differences in metabolic profile between the asthmatic phenotypes have also been confirmed by our team [28]. Environmental factors and metabolic markers in vivo of asthma might be novel points to illustrate the pathogenesis of asthmatic phenotypes and the possible pharmacological mechanism of a potential medicine for asthmatic therapy. Furthermore, integration of microbial and metabolomics data may have promise for identifying the microbial influence on host physiology through production, modification, or degradation of bioactive compounds [29].

OVA-induced experimental murine asthma models display similar inflammatory and immune dysregulated status as the asthmatic patients with distinct phenotypes [30,31], including eosinophil-dominated asthma-like airway inflammation and neutrophil-dominated asthma-like airway inflammation [32]. Therefore, in the present study, we established OVA-induced murine asthma models with different phenotypes, assessed the therapeutic effect of RWC on the experimental asthma, and explored the immune pharmacological mechanism of RWC on the inflammatory phenotypes. Furthermore, the pharmacological network based on multiomics was also constructed for an in-depth understanding of the mechanism of RWC.

## 2. Results

**Figure 1:**
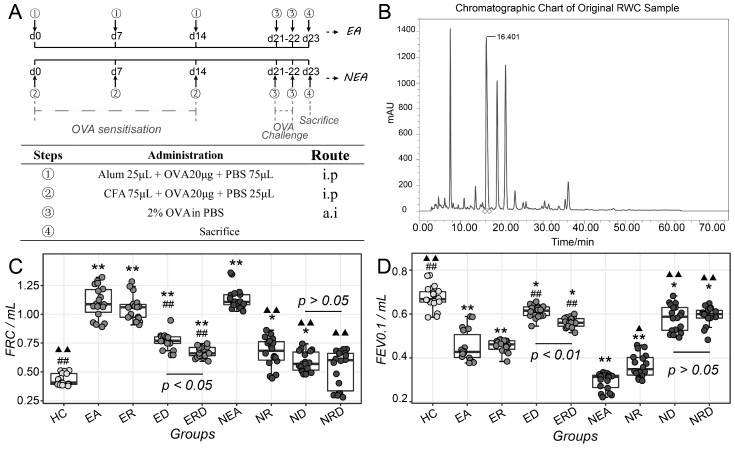
Flowchart and administration of establishing asthmatic phenotypes, including sensitization, challenge, and sacrifice (**A**). The characteristic chromatographic chart of testing injection samples after 20 consecutive injections (**B**). The statistics of functional residual capacity (FRC) (**C**) and forced expiratory volume in 0.1 s (FEV0.1) (**D**) of mice in all groups. i.p: intraperitoneal; a.i: atomized inhalation. HC: healthy control; EA: eosinophilic asthma murine model; ER: EA treated with RWC; ED: EA treated with DEX; ERD: EA treated with RWC and DEX; NEA: non-eosinophilic asthma murine model; NR: NEA treated with RWC; ND: NEA treated with DEX; NRD: NEA treated with RWC and DEX. * *p* < 0.05 vs. HC; ** *p* < 0.01 vs. HC; ## *p* < 0.01 vs. EA; ▲ *p* < 0.05 vs. NEA; ▲▲ *p* < 0.01 vs. NEA. The statistics between ED and ERD and ND and NRD were provided directly in the plot.

OVA-induced murine models of airway inflammation with different phenotypes, including EA and NEA, were established according to the flowcharts displayed in Figure 1A under a previous modeling theory [32]. The pharmacological mechanism of RWC, including under combined treatment with steroid, was further investigated using immunological and systematic biological techniques.

### 2.1. Improvement of Spirometry of Asthmatic Models and Quality Assessment on RWC

The quality of RWC injection based on the water extract of RWC root was evaluated according the quantitative levels of salidroside using optimized HPLC (high-performance liquid chromatography) conditions, as listed in Table S1. The stability and durability of the HPLC conditions (including stability, repeatability, intermediate precision, linear, and accuracy) was firstly assessed and displayed in Figures S1–S6 and Tables S2–S6. The level of salidroside in the injection of RWC was estimated corresponding to the retention time of 16.401 min, as displayed in Figure 1B, and was quantified as 6.1 mg/mL, which met the quality requirement in Chinese Pharmacopoeia (version 2015) on the quality of RWC injection.

Spirometry was used to assess lung function, as shown in Figure 1C,D. EA groups and NEA groups displayed similar general changing trends. Compared to healthy control (HC), EA and NEA model groups showed a higher level of pulmonary functional residual capacity (FRC). In EA groups, individual treatment with RWC did not alleviate the high FRC, though DEX alone could decrease it significantly, while the combined administration with dexamethasone (DEX) further remarkably reduced FRC. Despite that, FRC in the combination treatment group is still higher than HC. In the NEA groups, the high level of FRC could be not only be reduced by RWC but also by the combination treatment, which nearly lowered the level of FRC to HC (*p* > 0.05). As displayed in Figure 1D, the effect on FEV0.1 (forced expiratory volume in 0.1 s) after treatment in EA and NEA groups was similar to FRC. However, the combined treatment in NEA groups did not result in recovery of FEV0.1 to normal levels (*p* < 0.05 vs. HC).

### 2.2. Alleviatory Effect of RWC on AHR and Asthmatic Inflammation

**Figure 2:**
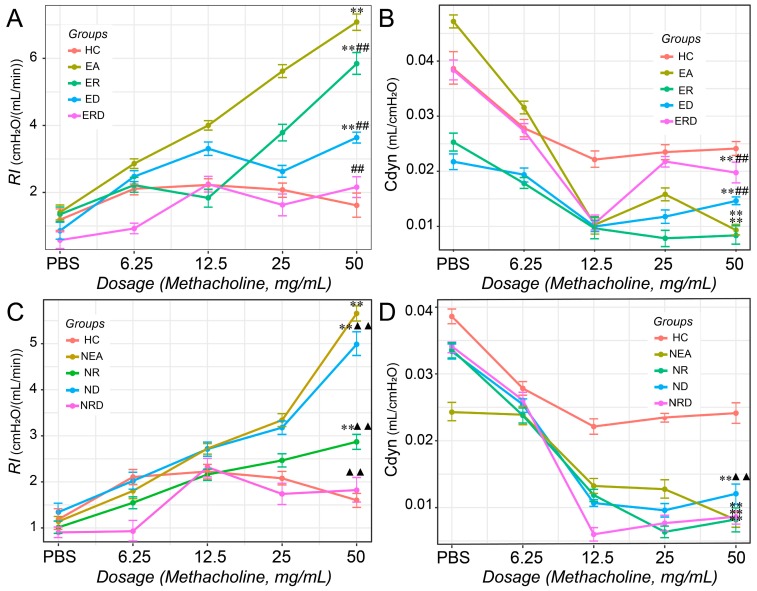
The testing results of airway hyperresponsiveness (AHR) of mice in the groups based on the inflammatory phenotypes. The airway of mice was stimulated with methacholine at different concentration (PBS, 6.25 mg/mL, 12.5 mg/mL, 25 mg/mL, and 50 mg/mL). Airway resistance of inspiration (RI) in EA (**A**) and NEA (**C**) and dynamic compliance (Cdyn) in EA (**B**) and NEA (**D**) were statistically evaluated based on the data of 50 mg/mL treatment. * *p* <0.05 vs. HC; ** *p* <0.01 vs. HC; # *p* <0.05 vs. EA; ## *p* <0.05 vs. EA; ▲ *p* < 0.05 vs. NEA; ▲▲ *p* < 0.01 vs. NEA.

The phenotype-specific evaluation of AHR is shown in Figure 2. Compared with HC, the airway resistance of inspiration (RI) indexes were remarkably increased in both model groups (EA and NEA), which indicted successful establishment of the OVA-induced asthma-like murine models with different inflammatory phenotypes. As for the treatment groups of EA, single administration with RWC did not inhibit AHR, while DEX and combined treatment could significantly reduce the high level of AHR in EA (Figure 2A). In NEA groups, however, compared to the model, DEX did not significantly alleviate AHR until combined with RWC. Moreover, single treatment with RWC also inhibited AHR of NEA at a large scale (Figure 2C).

In addition, pulmonary dynamic compliance (Cdyn) was also examined (Figure 2B,D). Compared with HC, Cdyn of the murine models decreased significantly, especially in NEA, which further indicated the success of experimental murine asthma models. In EA groups, combined treatment protected Cdyn from decreasing. However, all treatments did not recover Cdyn to the level of HC (*p* < 0.01 vs. HC, 50 mg/mL).

**Figure 3:**
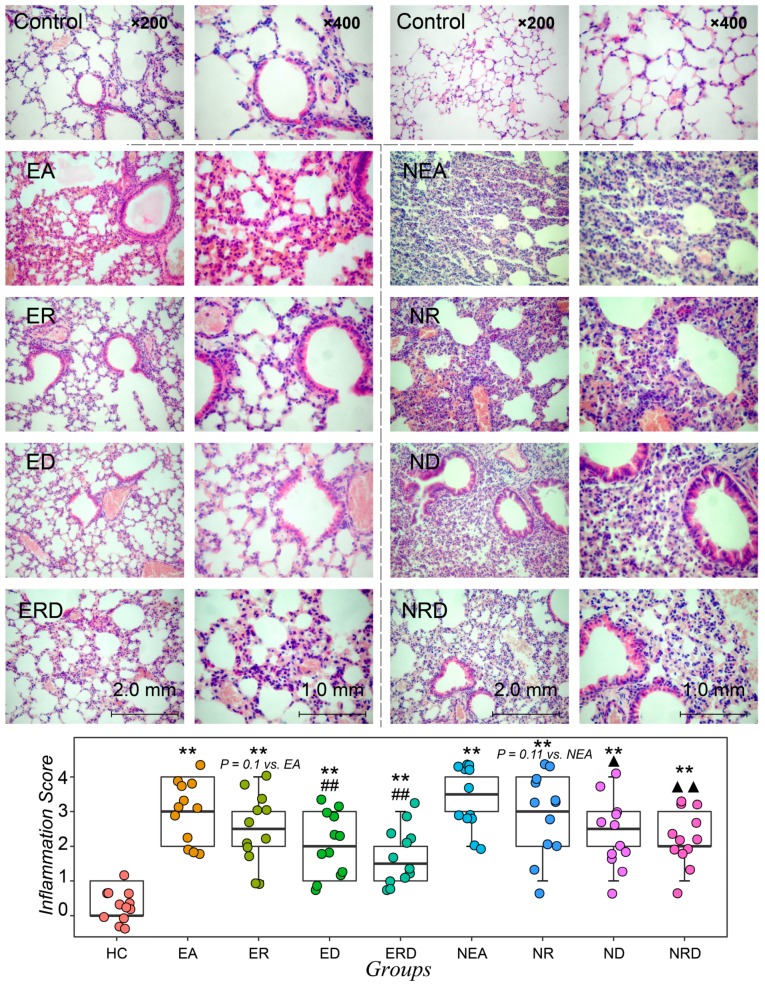
The H&E staining results of the left lung of mice in all groups under different magnifications (200× and 400×). The histopathological assessment score of inflammatory status of all groups was based on the assessment criteria provided in Section 4. ** *p* < 0.01 vs. HC; ## *p* < 0.05 vs. EA; ▲ *p* < 0.05 vs. NEA; ▲▲ *p* < 0.01 vs. NEA.

Formalin-fixed paraffin-embedded lung tissues of all groups were stained with H&E (hematoxylin and eosin) and displayed in Figure 3. The inflammation status was also quantitively scored and statistically assessed (Figure 3). In the HC groups, the frame of lung tissues and the structure of alveolus is clear and without significant infiltration of inflammatory cells. In EA model, the structure of lung is disordered and infiltered with eosinophils severely. RWC only could not alleviate eosinophilic inflammation (*p* > 0.05), but single DEX and combined treatment with RWC reduced the inflammation status. In the NEA groups, the morphology of the lung tissues of model group was obviously and significantly disordered, with significant infiltration of neutrophils and fewer normal bronchus structures observed. Individual and combined treatments with RWC and DEX alleviated neutrophilic inflammation and improved bronchus structure, especially in the combined treatment group (*p* < 0.01).

### 2.3. Improvement of Th2/Th1 Imbalance and Regulatory Effect on Th17/Tregs

**Figure 4:**
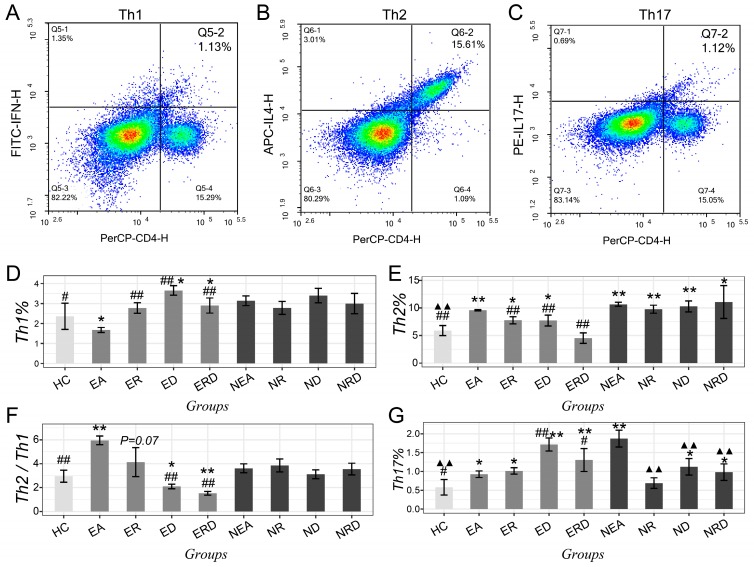
Typical flow cytometry dot plots of helper T cell clusters (including Th1/Th2/Th17, from one of the samples of all groups). (**A**) Th1 (CD4+–IFN-γ+) in lung tissue; (**B**) Th2 (CD4+–IL-4+) in lung tissue; (**C**) Th17 (CD4+–IL-7+) in lung tissue. The statistical data of (**D**) Th1 cells, (**E**) Th2 cells, (**F**) Th2/Th1 ratio and (**G**) Th17 cells in mice lung tissues with flow cytometry technique. * *p* < 0.05 vs. HC; ** *p* < 0.01 vs. HC; ## *p* < 0.01 vs. EA; # *p* < 0.05 vs. EA; ▲▲ *p* < 0.01 vs. NEA.

**Figure 5:**
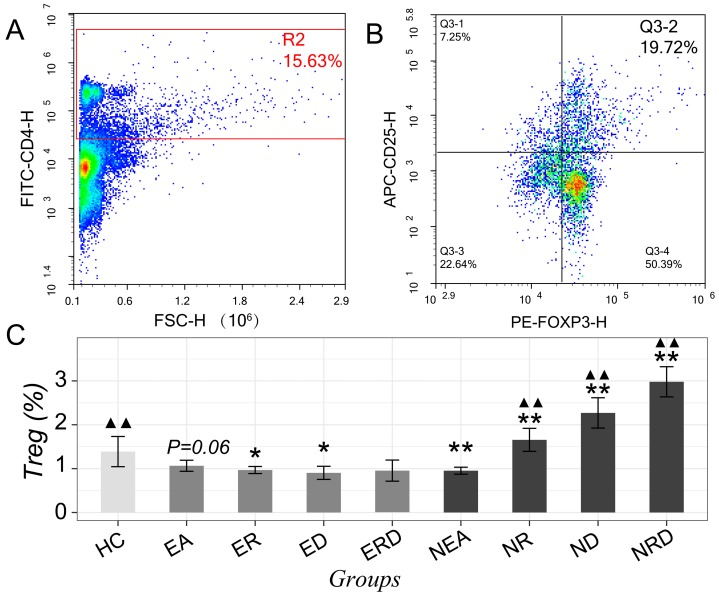
Typical flow cytometry dot plots of regulatory T cell clusters. (**A**) CD4 positive and negative cells in lung tissue; (**B**) Treg cells (CD4+–CD25+–Foxp3+) in lung tissue. (**C**) The statistical data of Tregs cells in mice lung tissues with flow cytometry technique. * *p* < 0.05 vs. HC; ** *p* < 0.01 vs. HC; ▲▲ *p* < 0.01 vs. NEA.

The Th2/Th1 imbalance and the level of Th17 in mice lungs was also evaluated with flow cytometry and gated as Figure 4A–C and Figure 5A,B. Compared with HC, the level of Th1 in the EA model decreased but improved significantly after treatments in all administration strategies (*p* < 0.01). Th2 cells in the EA and NEA model groups increased significantly but decreased remarkably in EA after all treatments, in particular, with combined treatment, which nearly lowered Th2 to normal levels (*p* > 0.05 vs. HC). However, as for NEA groups, none of were able to alleviate the high levels of Th2. The imbalance of Th2/Th1, referred as the ratio of Th2/Th1 cells, was most significantly dysregulated in the EA model group. EA mice treated with RWC displayed a trend of lowering this imbalance (*p* = 0.07 vs. HC). However, when treated with DEX, the imbalance was downregulated significantly, especially for the combination treatment. However, in NEA groups, the ratios of Th2/Th1 have an increasing trend but was not considered statistically significant, even after treating with RWC either individually or in combination. The changing trends of these T lymphocytes were not consistent between EA and NEA groups. Similar results were also observed in the spleen, as shown in Figure S7.

As for Th17, compared to HC, its level in EA increased slightly but increased more significantly in NEA. All treatment methods in the EA groups did not reduce the high level of Th17 but were increased after treatment with DEX. From this increase, RWC seems to be protective. However, in NEA groups, the administration of RWC alone could reduce Th17 to the level of HC (*p* > 0.05 vs. HC). Although DEX alone and the combination method could also regulate the level of Th17 in NEA significantly, these treatments did not recover levels to those observed in normal.

Tregs in lung tissues were also detected with flow cytometry and gated as Figure 5A,B. Compared to HC, Tregs in EA decreased slightly and did recover to normal level under the treatment with RWC or DEX separately (*p* > 0.05 vs. HC). The combined administration only increased Tregs to normal level at a small scale (*p* > 0.05 vs. HC or vs. EA). However, Tregs of the NEA model decreased remarkably in comparison with HC. After the treatment with either RWC, DEX, or their combination, pulmonary Tregs increased sharply (*p* < 0.01 vs. NEA), especially after the combined administration, even higher than HC (*p* < 0.01 vs. HC). But the levels of Tregs in spleen seem to have a trend of decreasing after the treatments in both phenotypes, as shown in Figure S8.

### 2.4. Influence on Th1/Th2/Th17-Related Cytokines

**Figure 6:**
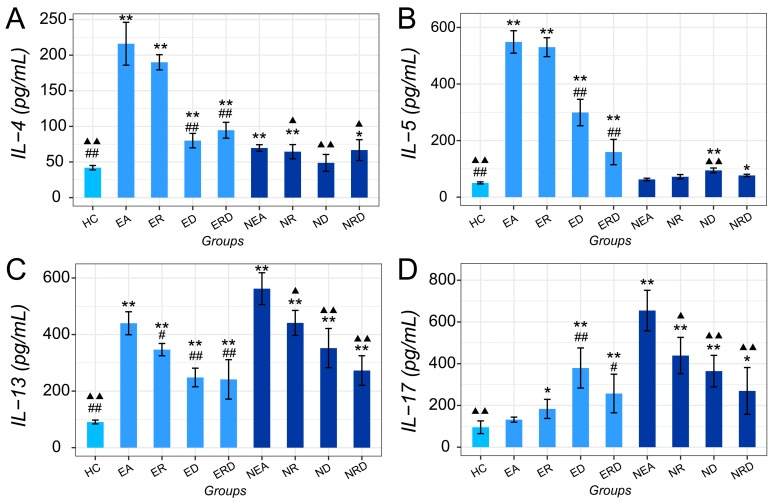
The results of asthma-related cytokines, including IL-4 (**A**), IL-5 (**B**), IL-13 (**C**), and IL-17 (**D**) in mice bronchoalveolar lavage fluid (BALF) of all groups. * *p* < 0.05 vs. HC; ** *p* < 0.01 vs. HC; # *p* < 0.05 vs. EA; ## *p* < 0.01 vs. EA; ▲ *p* < 0.05 vs. NEA; ▲▲ *p* < 0.01 vs. NEA.

Th1/Th2/Th17 related cytokines were quantified with ELISA assay, and results are shown in Figure 6. Compared to HC, IL-4, IL-5, and IL-13 in the EA groups increased significantly in the model group and were inhibited significantly by DEX. Single RWC treatment could only reduce the level of IL-13 (*p* < 0.05 vs. EA). In the EA model, the combined treatment sharply decreased the level of IL-4, IL-13, and especially IL-5. In the NEA model, IL-4 and IL-13 increased significantly in comparison to HC, but decreased after the treatments. IL-5 in NEA only showed an obvious increase after treatment with DEX.

Besides, IL-17 in EA did not change significantly in the model group but increased after therapy. However, in the NEA groups, the level of IL-17 evidently increased sharply in the model group (*p* < 0.01 vs. HC). Treatment with either RWC, DEX, or their combination could significantly reduce the level of IL-17 in NEA. Despite that, they were still higher than in HC (*p* < 0.01 vs. HC).

### 2.5. α-Diversity and β-Diversity of the Microbiota in Lower Airway

Microbiota in the lower airway of the OVA-induced murine models was detected using 16S rDNA sequencing techniques. The quality of the sequencing was over QC20 (Figure S9) and met the requirement for further analysis. An initial bioinformatic evaluation of the microecological features including α-diversity and β-diversity was performed.

**Figure 7:**
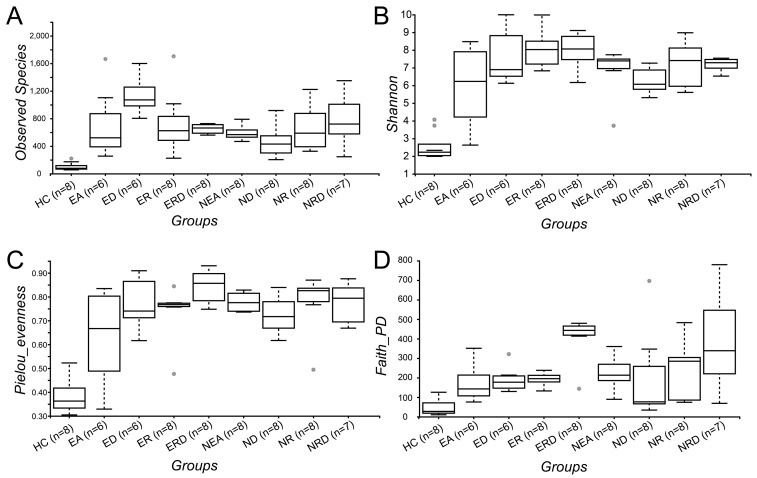
The box plots of α-diversity indexes, including (**A**) observed species index (OBS), (**B**) Shannon index, (**C**) Pielou’s evenness index, and (**D**) Faith’s PD index.

Compared to HC, as displayed in Figure 7A–C, the diversity (OBS, observed species index), richness (Shannon index) and evenness (Pielou’s evenness index) of all EA and NEA groups increased significantly, indicating a huge quantity of microorganisms in the airway of the OVA-induced mice. Although the multiple pairwise comparisons among many groups showed good significance (*p* < 0.05), few pairs passed the false discovery rate (FDR) test (*q* < 0.05). As shown in Tables S7–S9, only NEA treated with DEX showed a significantly lower level when compared with EA phenotype treated with DEX, and passed the FDR test.

The diversity index based on phylogenetic diversity (PD), Faith’s PD diversity index, was also used here to evaluate the diversity of the microbiota. As shown in Figure 7D and Table S10, the diversity of microbiota in all experimental asthma groups was significantly higher than HC. However, as reflected by Faith’s PD index, the diversity of EA phenotype treated with the combined strategy increased significantly compared with EA model (*p* < 0.05 and *q* < 0.05). However, no diversity changes were observed in the treated NEA groups when comparing with the NEA model. Moreover, other typical α-diversity indexes were all calculated (Figure S10) and similar results were also reported.

**Figure 8:**
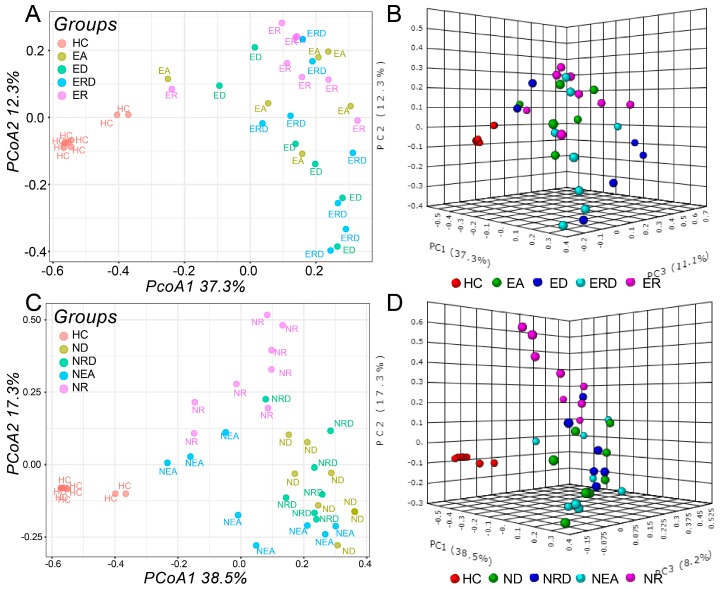
PCoA plots of β-diversity analysis of all groups, which are based on the Bray–Curtis index. PCoA plots (PCoA1 = 37.3%, PCoA2 = 12.3%, PCoA3 = 11.1%) of EA groups in (**A**) 2D and in (**B**) 3D. PCoA plots (PCoA1 = 38.5%, PCoA2 = 17.3%, PCoA3 = 8.2%) of NEA groups in (**C**) 2D and in (**D**) 3D.

β-Diversity based on principal coordinates analysis (PCoA) was evaluated according to each phenotype. The OVA-induced murine models and HC group were separately distributed in the plot and clustered within their groups (Figure S11). PCoA based on phenotypes revealed that there was no significant difference in the general structure of microbiota among the EA groups as displayed in Figure 8C,D. However, in the NEA groups, the groups with treatment are significantly different from NEA model group because they are separated from NEA model. However, as shown in Figure 8E,F, NEA administrated with DEX or the combination treatment seemed to be more similar. The difference among NEA groups was further confirmed using the NMDS (non-metric multidimensional scaling) method (Figure S12). Moreover, Adonis/PERMANOVA also determined a significant difference among the NEA groups (*p* < 0.0001).

### 2.6. Taxonomic Differences in Microbiota after Treatment

Differentiated taxonomies of the microbiota in different groups and phenotypes were analyzed with LEfse (linear discriminant analysis (LDA) effect size), a newly developed method which combines LDA with Kruskal–Wallis and Wilcoxon tests. Different taxonomies were identified among the model groups (including the EA model, NEA model, and HC group), EA groups (including EA model, ED group, ER group and ERD group), and NEA groups (including the NEA model, ND group, NR group, and NRD group).

**Figure 9:**
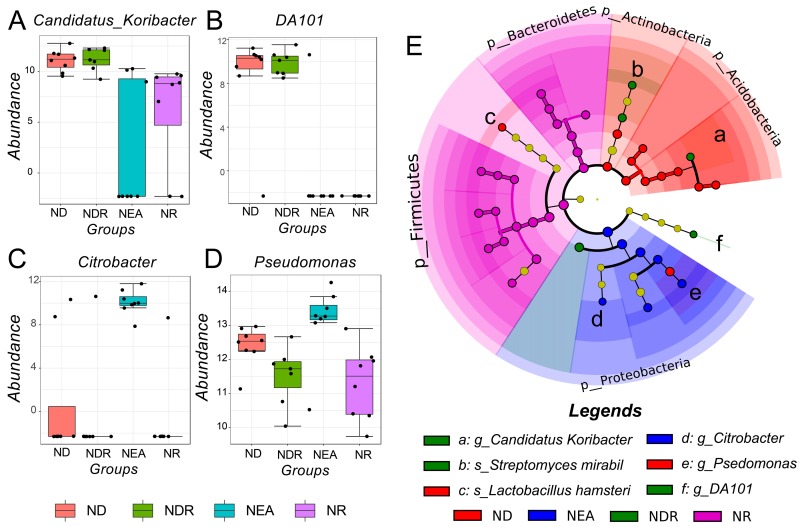
Distinct taxonomies in the NEA group. (**A-D**) Statistical results of all significantly distinct genera (*p* < 0.05, LDA > 4) in all NEA groups, including the original counts and log-transformed data. (**E**) Cladogram plot of different taxonomies (including genera and species, etc.) in the NEA groups.

In the model groups, the distinct taxonomies are summarized in Figures S13–S14 and Table S11. In the EA groups, we did not report any significant difference of taxonomies in the levels of phylum, genus, and species. In NEA, no significant differences were observed at the level of phylum (*p* > 0.05 or LDA < 4). However, a distinction involving 4 genera (Figure 9A–D) and 2 species (Table 1) was observed. The cladogram relationship of the different taxonomies in the NEA group is displayed in Figure 9E.

### 2.7. Difference of Systematic and Pulmonary Metabolic Pattern

To further identify the pathogenesis of asthma and the potential metabolic function of the differentiated microbiota in lower airway, non-targeted metabolomics was performed using UPLC-QTOF-MS/MS. The durability and stability of the chromatographic and spectrometric system was first evaluated. As displayed in Table S12, the relative standard deviation (RSD) of the mass-to-charge ratio was less than 0.001%, the RSD of the retention time of the chromatography was less than 1%, and the RSD of the chromatographic peak area was less than 6%. These data indicated a stable chromatographic and spectrometric system well-suited for following metabolomics analysis.

Principal component analysis (PCA) of the serum metabolomics was well-fitted (R2X = 0.921, Q2 = 0.712) and as displayed in Figure S15A,C, QC samples were highly clustered at the center of the plot, which further indicated the good stability of the metabolomics system. Generally, the treated groups with the exception of NRD obviously separated with the models and HC. As for NRD, it was closer to the HC and models, which indicted a potential significant change in metabolic profile. However, as displayed in Figure S15B,D, the metabolic profile of BALF showed significant differences among the different groups.

**Figure 10:**
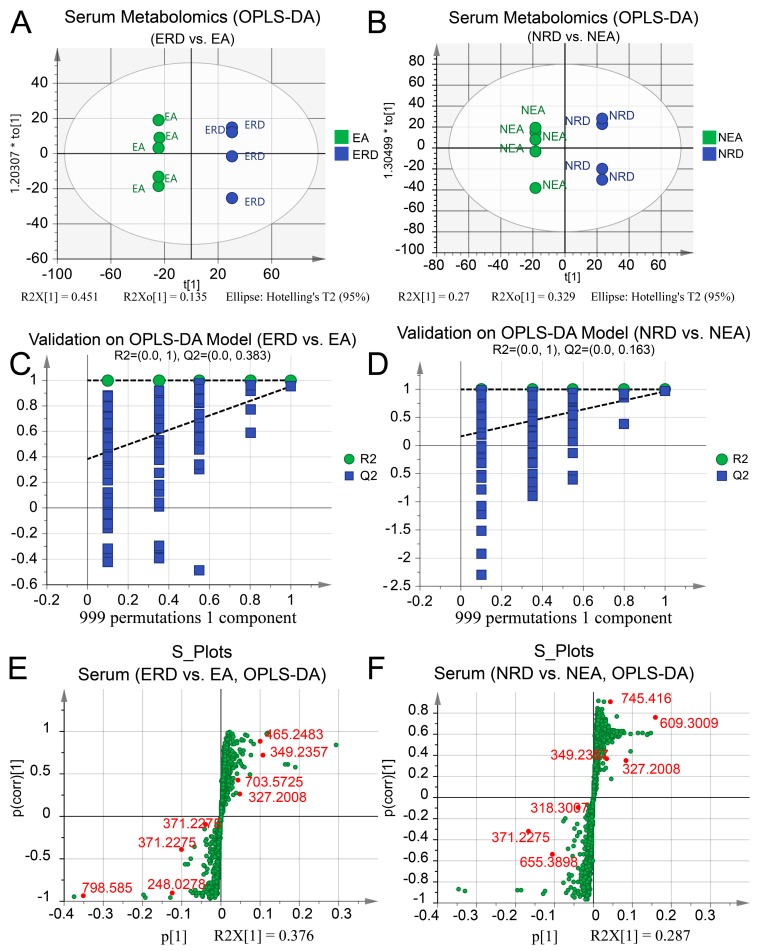
OPLS-DA score plots and the permutation tests of serum metabolomic profile with the identified metabolites displayed in the groups. OPLS-DA models of (**A**) ERD vs. EA, *p* < 0.001 and (**B**) NRD vs. NEA, *p* < 0.001. The permutation test on the OPLS-DA of (**C**) ERD vs. EA and (**D**) NRD vs. NEA. The S-plots based on OPLS-DA of (**E**) ERD vs. EA and (**F**) NRD vs. NEA and identified metabolites in serum. All *p*-values were estimated using the CV-ANOVA test. Ellipse in the OPLS-DA panels represents Hotelling’s T2 test with a confidence interval of 0.95. The counts of permutation tests are 999.

**Figure 11:**
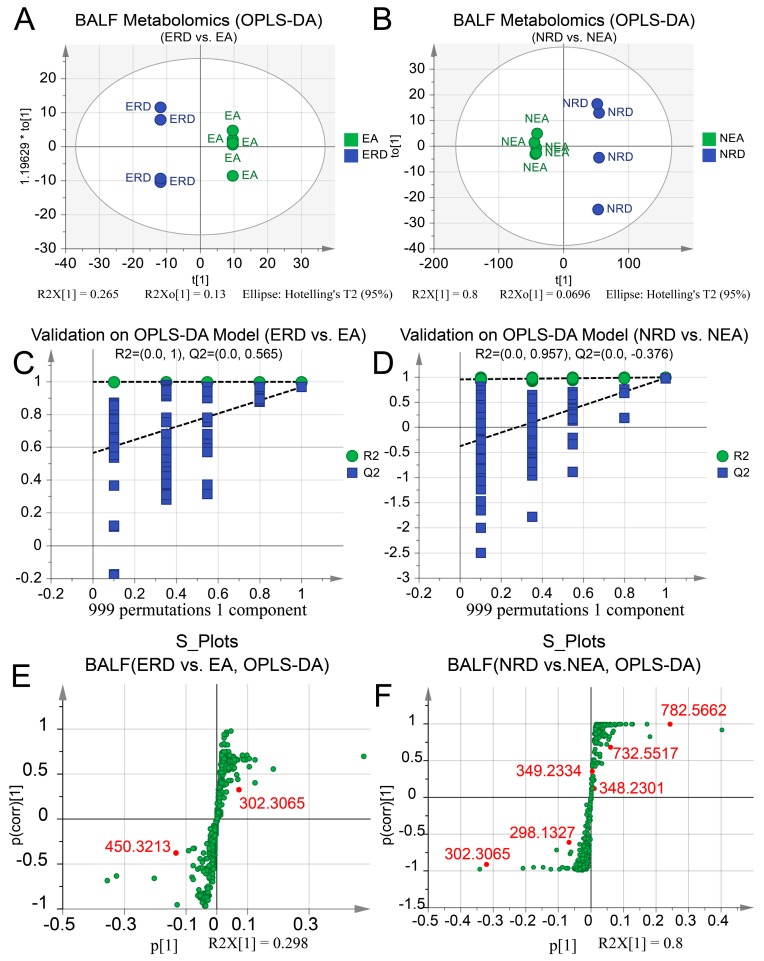
OPLS-DA score plots and the permutation tests of BALF metabolomic profile with the identified metabolites displayed in the groups. OPLS-DA models of (**A**) ERD vs. EA, *p* < 0.001 and (**B**) NRD vs. NEA, *p* < 0.001. The permutation test on the OPLS-DA of (**C**) ERD vs. EA and (**D**) NRD vs. NEA. The S-plots based on OPLS-DA of (**E**) ERD vs. EA and (**F**) NRD vs. NEA and identified metabolites in BALF. All *p*-values were estimated using the CV-ANOVA test. Ellipse in the OPLS-DA panels represents Hotelling’s T2 test with a confidence interval of 0.95. The counts of permutation tests are 999.

OPLS-DA models of serum were first established and displayed good separation (Figure 10A,B). CV-ANOVA and permutation tests showed a high significance and confidence for further identification of the distinct metabolites (Figure 10C,D). Similar discriminant models of BALF samples were also established and validated with the CV-ANOVA and permutation (Figure 11). The permutation line was under the random line, which indicated a series of well-fitting discriminant models. Moreover, the OPLS-DA models of EA vs. NEA, EA vs. HC, and NEA vs. HC of serum or BALF samples were also established to further clarify the pathogenesis of the experimental asthmatic inflammation phenotypes as shown in Figures S16−S18.

All differentiated metabolites in either serum or BALF were identified and displayed with S-plots in Figure 10E,F and Figure 11E,F, respectively. More details of the identified metabolites according to their MS/MS fragmentation pattern are summarized in Table 2. Total ion chromatogram and base peak ion chromatogram of serum and BALF samples are displayed in Figures S19 and S20, respectively. The identification and comparison details of the metabolites are provided in Figures S21–S54.

### 2.8. Perturbed Metabolic Pathways

**Figure 12:**
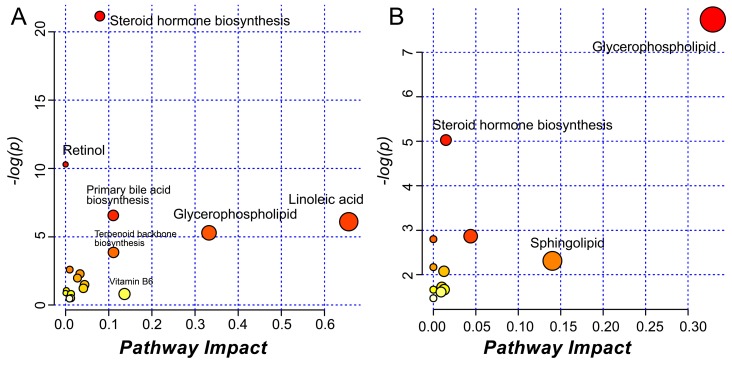
Perturbed metabolic pathways of (**A**) Serum and (**B**) BALF from MetaboAnalyst 4.0.

The perturbed metabolic pathways of serum and BALF were analyzed using MetaboAnalyst 4.0 as based on the identified metabolites and results are displayed in Figure 12. A total of 7 significantly changed pathways in serum and 3 in BALF were observed. However, the extent of perturbation differed for the different metabolic pathways. In detail, 3 of the changed metabolic pathways displayed extreme significance (impact > 0.1 and −log(*p*) > 4). Some pathways showed potential significance (impact > 0.1 or −log(*p*) > 2), but other pathways only had a trend of association (−log(*p*) > 2). Moreover, different OPLS-DA models involved different perturbed metabolic pathways because of their specific differentiated metabolites. The identified metabolic pathways are summarized in Table S13.

### 2.9. Multiomics Joint Exploration and Pharmacological Network of RWC

The metabolic function of the microbiota in NEA groups were further predicted with PICRUSt and produced a gene list (KEGG orthology format) of the potential distinct microbiota (Table S14 in part). Joint multiomics data were analyzed and reported a potential significance. The predicted genes of the colonized microbiota might be directly related to the perturbation of inflammation-related metabolic pathways, glucocorticoid resistance of the experimental asthma, and the susceptibility of the murine asthma (Figure S55A–C). BALF-based multiomics analysis revealed that sphingolipid and glycerophospholipid metabolic pathways might be directly influenced by the microbiota (Figure S55D).

Immunological and system biological research on the pharmacological mechanism of RWC has been performed. The pharmacological network was also established as Figure S56. On the one hand, RWC could generally regulate the balance of Th1/Th2 and Th17/Tregs, influencing the release of related cytokines and mucus hypersecretion; on the other hand, RWC might have an effect on the pathogenesis of the OVA-induced airway inflammation and systematic metabolism by intervening in the airway microbiota, which might be conversely regulated by systematic immune status.

## 3. Discussion

In the present study, we confirmed the quality of RWC injection by first quantifying the level of salidroside and establishing OVA-induced murine asthma models with different airway inflammation phenotypes. Treatment with RWC and/or DEX could improve the spirometry results, and alleviate AHR and pulmonary inflammatory status. Immunopharmacological studies revealed the regulatory effect on the imbalance of Th1/Th2 and Th17/Tregs, and the release of related cytokines. High-throughput sequencing of 16S rDNA of the microbiota reported an increased diversity, richness, and evenness in the murine models. Several taxonomic units were identified between the inflammation phenotypes. Treatment with RWC or DEX could significantly increase the diversity of the microbiota in EA and change the taxonomic levels of 4 genera and 2 species in NEA. Systematic and pulmonary metabolic profiles were changed in murine models after the treatment. A total of 34 differentiated metabolites and 8 perturbed metabolic pathways were identified. The joint multiomics study revealed that the microbiota colonized the lower airway might be related to the susceptibility of asthma and steroid resistance in NEA, which involves several metabolic pathways. Knowledge-based integrated systematic biological research revealed the pharmacological network and mechanism of RWC.

RWC could alleviate the AHR of NEA. Combined treatment with RWC and DEX could improve the spirometry results and AHR status of the both OVA-induced airway inflammation models, the pulmonary dynamic compliance of EA, and the steroid resistance of NEA. The inflammatory status of granulocyte infiltration of EA and NEA was also relieved. The improvement of the spirometry results and severity of the inflammation displayed a well-correlated changing trend. Immunological analysis showed that RWC could increase the level of Th1 in EA and reverse the imbalance of Th2/Th1. At the same time, it participated in the regulation of Th17. Wang J et al. observed the level of inflammatory mediators and reported that salidroside could balance Th2/Th1 [33], which is consistent with our results. In the present study, combined treatment with RWC and DEX negatively regulated Th17, whose therapeutic effect was even better than DEX. It is worth mentioning that DEX induced the pulmonary increase of Th17 in EA, which might be associated with the accumulation or survival of neutrophils in the airway [34]. However, from the increase of Th17 in this process, RWC seems to be protective. Tregs, as previously described, might take part in the inhibitory regulation of inflammation and the increase of Th17 [35,36]. Herein, we discovered that RWC might increase pulmonary levels of Tregs in NEA, especially when the treatment is combined with DEX. However, whether this effect was driven by salidroside needs further investigation regarding its molecular pharmacological mechanism.

The investigation of the cytokines related to OVA-induced inflammatory phenotypes revealed that treatment with RWC alone cannot reduce the high level of the cytokines. However, this was achieved using combined treatment, and the reduction included IL-5, a critical cytokine involved in chemotactic recruitment of eosinophils into the lung [37]. Moreover, IL-13, a cytokine tightly associated with the secretion of mucus, was observed to be increased in NEA but decreased after treatment, which indicated a potential unknown mechanism [38]. As for IL-17, it has been considered to take part in the AHR of NEA and play an antagonistic regulatory role to Tregs [39,40,41]. In our study, it was found that RWC might, at least in part, improve the counts and function of Tregs by reducing IL-17. Similarly, the high level of IL-17 in EA might also be associated with the accumulation of neutrophils after treatment with steroids. As reported previously, salidroside could restrict the phosphorylation and activation of p38 MAPK [18], though this point is controversial [42]. Activation of p38 MAPK can reduce the biological activity of MAPK phosphorylase I and further induce steroid resistance [20]. In addition, the mucus of airways, which is regulated by IL-13, and the inflammatory status have both been confirmed to be related with the colonization of microbiota in airways [43,44]. Given the complex pathogenesis of the disease and its unclarified immune mechanism, systematic investigation on the pharmacological mechanism of RWC, using omics techniques, is necessary.

The microbiome study was first performed in an environmental factors-restricted limited animal barrier facility. Bioinformatic analysis of the ecological features of the microbiota in the EA and NEA groups revealed a high diversity, richness, and evenness, which was different from our recent clinical study [9] but was consistent with previous murine research [45]. Traditionally, studies of the microbiome have mainly focused on differentiated genera but, in present study, we identified 20 differentiated species between the asthmatic phenotypes and 2 species among the NEA groups with high confidence under the high OTU classification threshold. However, until now, the research to date into the biological role of bacterial species has not been sufficient enough to illustrate the detailed mechanism of the differentiated species discovered in the present microbiota study. Despite this, some species have been investigated. For example, *Haemophilus parainfluenzae* has been discovered to be tightly associated with the asthmatic resistance to steroids [19]. *Propionibacterium granulosum* was also reported to regulate the immune function of airways [46]. *Prevotella melaninogenica*, as a common bacterium, was found at high levels in EA [47,48], but the detailed pathogenic mechanism has never been reported. Other species, including *Streptococcus luteciae* [49], *Clostridium sordellii* [50], and *Clostridium perfringens* [51], as the members of gut microbiota, have been studied extensively, but their role in the pathogenesis of asthmatic phenotypes needs further exploration.

Treatment with RWC and/or DEX within the murine inflammatory phenotypes showed a larger influence on the structure of microbiota in NEA with 4 differentiated genera and 2 species found. In details, the combined treatment with RWC and DEX increased the level of *Candidatus Koribacter*, *DA101* and decreased the level of *Citrobacter* and *Pseudomonas* in airway. It was reported that *Citrobacter* are involved in the antagonized effect of IL-17 on IL-25-mediated airway inflammation [52], while the reduction of *Pseudomonas* might be closely related to the alleviation of bronchiectasis. As for the distinction at species level, it was reported that *Proteus mirabilis*, whose high levels might be related to the infection of microbiota [53,54] was increased after treatment with DEX, but decreased when treated with RWC. The other distinct species, *Lactobacillus hamster*, became more abundant after the combination therapy. However, the potential mechanism is unknown. Metabolic prediction of the distinct microbiota based on PICRUSt discovered a large quantity of enzymes, which indicated the potential for in-depth understanding of the unknown mechanism.

Systematic metabolomics displayed a significant difference in metabolic profiles between the experimental murine asthmatic phenotypes, which is consistent with our research on clinical samples [28]. According to the fitting degree of OPLS-DA models, we discovered that compared to HC, NEA seemed to be perturbed at a larger scale than EA. Combination treatment with RWC and DEX could influence the metabolic profile of the both asthmatic phenotypes. A large number of metabolites identified in present study revealed that the combined treatment could significantly influence the linoleic acid, glycerophospholipid, and primary bile acid biosynthesis metabolism. Moreover, the therapy has a potential effect on systematic steroid hormone biosynthesis, and retinol and terpenoid backbone biosynthesis metabolism. In detail, in our studies, we observed distinct level of hormones, including hydroxytestosterone and hydroxydehydroepiandrosterone. Testosterone is an important hormone which may alleviate ILC2-mediated airway inflammation [55]. The presence of its metabolite, hydroxytestosterone, may indicate a perturbation in testosterone. Despite hydroxydehydroepiandrosterone having been confirmed to have extensive biological activities, including in immune regulation [56], its role in the pathogenesis of asthmatic phenotypes and pharmacological mechanisms of the therapeutic strategies needs further investigation. Ursodeoxycholic acid, a chemical component of primary bile acid metabolism, has been reported to suppress the infiltration of eosinophils into the airway by inhibiting the activity of dendritic cells [57]. Chenodeoxycholic acid, discovered to be perturbed, has been revealed by a previous study to have the potential to eliminate OVA-induced airway Th2 inflammation and lower the level of related cytokines [58].

Similar to our previous report on the metabolic features of clinical asthmatic patients, roles in linoleic acid, glycerophospholipid, and retinol metabolism were also discovered in murine models [28]. The intervention on primary bile acid metabolism may be related with the pharmacological effect of RWC on other organs. The biologically active form of vitamin B6, pyridoxal 5’-phosphate, has been observed at a lower level in asthmatics [59]. In present study, it increased in EA groups after treatment with RWC. However, the pharmacological details need further investigation. The metabolic profile of BALF was also analyzed in present studies with similar pathways observed, despite only two of them being regarded as significant. However, the number of metabolites identified in BALF were markedly lower than in serum because of the natural characteristics of the lung. Furthermore, given that the pulmonary metabolites may be from the colonized microbiota, the distinct metabolic pathways might result, in part, from the differentiated structure of the microbiota. Therefore, it is essential to illustrate the relationship between the microbiota, metabolic profile, and pathophysiological features of the experimental asthmatic phenotypes after treatment with RWC, especially in NEA.

Integrative analysis of microbiomes and untargeted metabolomes in disease contexts have also allowed for the identification of microbially and systematically produced compounds found to mediate biological effects [60], like pharmacological mechanisms. Herein, the joint multiomics studies revealed that the colonization of microbiota might cause steroid resistance by intervening in metabolic pathways, which is consistent with a previous report [61]. More in-depth analysis which combines the potential diseases and constructs an exhaustive interactions net may show us how the susceptibility of NEA is related to and regulated by the colonized microbiota via intervening metabolic pathways. The relationship between the colonized microbiota in lower airway and its local perturbed metabolic pathways were also combined, and two lipid metabolic pathways were reported as being involved. Targeted or lipid metabolomics studies are required to confirm and validate their differences and the potential immune role of microbiota in airway. 

In addition, knowledge-based analysis comprehensively revealed the pharmacological mechanism of RWC, including in regulating the innate and adaptive immune system and improving the structure of the microbiota which colonized airways and the metabolic profile of lower respiratory tracts. Multiple pharmacological targets is a characteristic feature of traditional Chinese medicine [62]. The multiomics and immune analyses here have revealed the pharmacological network and mechanism of RWC, but obvious limitations still exist. One is the limited metabolomics data for metabolite identification despite improvements being on the way. In addition, the granulocytes of infiltration need to be assessed more quantitatively using flow cytometry. Gut microbiota, as an essential factor in influencing the inflammatory status of asthmatics [63,64], should also be added into future joint in-depth multiomics studies. Moreover, in the future, the details of the omics data, including differentiated species markers, the predicted metabolic pathways, and the potential functional interactions need to be validated in-depth.

## 4. Materials and Methods 

### 4.1. Quantification on Salidroside in RWC Injection with HPLC

RWC injections were provided by Tonghua Yusheng Pharmaceutical Co., Ltd. (Approval Number of Chinese Medicine: Z20060361, Production Batch NO. X1001170405). Chromatographic columns (C18, 4.6 mm × 150 mm, 5 μm; Z2013112901, ACCHROM Technologies Co., Ltd.) were set at 30 ℃. The volume of each injection was 10 μL. Mobile phase A was a solution of acetonitrile–methanol (50:50 (*v*/*v*), Thermo Fisher Scientific Inc., Fisher Chemical, Waltham, MA, USA, Cat. A452-1 and A998-4, respectively). Mobile phase B was the solution of aqueous phosphoric acid (0.07%). The chromatographic conditions were set as described in Table S1. The wavelength of the UV detector was 278 nm.

The chemical standard salidroside (YZ-110818, from National Institutes for Food and Drug Control, Beijing, China) was weighed precisely and dissolved with methanol at 10.0 mg/mL as a standard solution for quantification. All solution samples were filtered with microporous membrane (0.22 μm) and injected into the system. Stability, repeatability, Intermediate precision, linear, and accuracy were first evaluated. Twelve consecutive injections of an RWC sample were performed for each quantification. Chromatographic data were collected and processed with Empower software (Waters Corporation, Milford, MA, USA. v3.0).

### 4.2. Experimental Animal and Procedure of Model Establishment

Specific pathogen-free (SPF) BALB/c female mice, weighing 20 ± 1 g, were purchased from Yi-si Corporation of Changchun City, China (Quality Certificate No. SCXK(JI)-2016-003). All mice were fed in individually ventilated cages of the Observation Facility of Animal Experimental in Barrier Environment (SPF level, College of Basic Medicine, Jilin University). The animal food was irradiated with cobalt-60. The drinking water was reverse osmosis water. All animals had ad libitum access to food and water. The animal experimenters were trained and qualified for the operations. All animal experiments were performed on a sterile clean bench.

Murine asthmatic phenotypes were established and optimized as previously described [32,65,66] and shown in Figure 1A. The mice were randomly grouped and sensitized with ovalbumin/aluminum (OVA/Alum) for EA or ovalbumin/complete Freund’s adjuvant (OVA/CFA) for NEA at day 0, 7, and 14. The mice were challenged with 2% OVA via an ultrasonic atomized inhalation machine (OMRON Corporation, Kyoto, Japan. NE-C900) at day 21 and 22. All mice were sacrificed at day 23. Treatment began at day 17 with RWC (i.g, 550 μL/20 g) and/or DEX (i.p, 20 μg/20 g) until day 23, as shown in Table 3.

All animal operational procedures were optimized according to the 3R principles and performed based on the guidelines for the administration of laboratory animals (Directive 86/609/EEC, 1986) and ARRIVE guidelines [67]. The checklist of the ARRIVE guidelines is shown in Table S15. This study was approved by the Institutional Animal Care and Use Committee of Jilin University (No. SCXK-2013-0001, 20 July 2018).

### 4.3. Spirometry and AHR Examination

Spirometry and airway hyperresponsiveness (AHR) were performed as previously described [68,69]. Briefly, all mice were anesthetized with pentobarbital (40 mg/kg) followed by endotracheal intubation. Spirometry was monitored with DSI Buxco^®^ FinePointe Pulmonary Function Test system (DSI Buxco, St. Paul, MI, USA. v2.3), with FRC and FEV0.1 data being collected. Resistance and compliance was examined with DSI Buxco^®^ FinePointe Resistance and Compliance system (DSI Buxco. v2.4) under the challenge of methacholine (Sigma, St. Louis, MO, USA. Cat. 62-51-1) with different concentrations (0, 6.25, 12.5, 25, and 50mg/mL). Resistance of inhalation (RI) and dynamic compliance (Cdyn) were recorded.

### 4.4. H&E Staining

After whole blood was collected and the mice anesthetized, the left lung lobes of 6 mice were obtained for H&E staining. In brief, the formaldehyde-fixed and paraffin-embedded lung tissues were sliced, dewaxed through xylene and a decreasing concentration of ethanol, stained with eosin and hematoxylin, differentiated with acid alcohol solution, and dehydrated with increasing concentrations of ethanol and xylene. The chemical reagents were of AR (analytical reagent) grade. Histopathological assessment of inflammatory status was performed with the following criteria as described previously [70]: 0, normal; 1, few cells; 2, a ring of inflammatory cells one-cell-layer deep; 3, a ring of inflammatory cells 2–4 cells deep; 4, a ring of inflammatory cells >4 cells deep. The degree of airway inflammation of all groups was determined by two independent analysts, who were blind to the experimental groups. The sections were observed with microscope (Olympus Corporation, 2-3-1 Nishi-Shinjuku, Tokyo, Japan, CKX41).

### 4.5. Flow Cytometry of Th1/Th2/Th17 and Tregs

Right lung lobes and spleens were fully ground and dissolved in sterile PBS buffer with red cell lysis buffer. Two-thirds of the cells collected were incubated with CD4 antibody conjugated with PerCP-CY5.5 and incubated with PMA (phorbol-12-myristate-13-acetate, BD Biosciences, 2350 Qume Drive San Jose, CA 95131, USA. Cat. 554656.) in RPMI-1640 medium (HYCLONE Corporation, 100 Results Way, Marlborough, MA 01752, USA. Cat. SH30023.01B) for 5 h. Then, the incubated cells were labeled with a mix of IL-14 antibody conjugated with APC, IL-17 antibody conjugated with PE, and IFN-γ antibody conjugated with FITC (BD Biosciences, 2350 Qume Drive San Jose, CA 95131, USA. Cat. 554436/550954/559502/554411, respectively).

The remaining third of lung and spleen cells were labeled with CD4 antibody conjugated with FITC, Foxp3 antibody conjugated with PE, and CD25 antibody conjugated with APC (eBiosciences, 5823 Newton Drive, Carlsbad, 92008, USA. Cat. E0082-1634/E01764-1641/4276862, respectively). All labeled cells were examined using flow cytometry (NovoCyte, ACEA Corporation, San Diego, CA, USA. 3005). Data were collected and processed with NovoExpress (ACEA Corporation. v1.3.0).

### 4.6. ELISA Assay

The serum of mice were obtained by removing the eyeballs while the mice were in a deeply anesthetized status. BALF was collected as previously described [71]. The BALF samples were concentrated 10-fold after lyophilizing at −60 ℃ and 10.0 Pa air pressure for 24 h for the quantification of IL-4, IL-5, IL-13, and IL-17 using ELISA kits (Thermo Fisher Scientific Inc., Invitrogen, Thermo Scientific Pierce, Waltham, MA, USA. Cat: BMS613/BMS610/BMS6015/BMS6001, respectively) according to the manufacturer’s instructions.

### 4.7. DNA Extraction and 16S rDNA Sequencing

BALF samples collected from the mice were centrifuged at 12,000× *g* for 30 min. The total DNA of was extracted using the CTAB DNA extraction method. Briefly, proteins were denatured with mercaptoethanol and removed with chloroform/isoamyl alcohol (24:1). CTAB/NaCl were mixed with the supernatant followed by the collection of precipitate. The DNA precipitate was dissolved in TE and extracted with phenol/chloroform/isoamyl alcohol (25:24:1), precipitated with ethanol, and then dissolved in TE. The above chemical reagents were AR grade. 

Total DNA was amplified in the 16S V3+V4 region using the primers 341F—CCTAYGGGRBGCASCAG and 806R—GGACTACNNGGGTATCTAAT and Phusion^®^ High-Fidelity PCR Master Mix with GC Buffer (New England Biolabs, 240 County Road, Ipswich, MA 01938, USA. Cat. M0532S). A sequencing library was constructed using TruSeq^®^ DNA PCR-Free Sample Preparation Kit (Illumina Corporation, 5200 Illumina Way, San Diego, CA, USA. Cat. 20015963) according to the manufacturer’s protocol. The quality of the library confirmed by agarose gel electrophoresis and qPCR was used for sequencing with Illumina HiSeq 2500 platform (Illumina Corporation. PE250). At least 100,000 tags were read for every sample.

### 4.8. Sample Preparation for Metabolomics and UPLC-Q/TOF-MS/MS Procedure

Serum and BALF samples were prepared as previously described [28,72]. Briefly, the protein was precipitated and removed with acetonitrile. The protein-free samples were lyophilized as described above. The dissolved residues were injected into the chromatographic system.

The Waters Xevo G2-S Quadrupole Time-of-Flight (QTOF) mass spectrometer coupled with a UPLC system (Waters ACQUITY UPLC system and BEH C18 column, 2.1 mm × 100 mm, 1.7 mm, Waters Corporation, Milford, MA, USA), as described in our previous study [28], was used here with the parameters of the UPLC-Q/TOF-MS/MS system set as before [28]. Data of the samples were collected in ESI+ mode. Quality control (QC) samples were prepared as described previously [28] and initially injected for the first 6 injections to ensure the stability and suitability of the system. The QC samples were also injected after every 4 samples throughout the whole procedure for real-time monitoring of the system. The data was collected with MassLynx (v4.1) and exported for further processing.

### 4.9. Omics Data Processing

Raw sequencing data were processed with QIIME2 (v2018-11) [73] which was based on Python (v2.7) and Miniconda 2.0 to merge the paired sequences, denoise the sequencing tags, remove the chimeras, and allow comparison to the taxonomic database (Greengenes v13.8). Operational taxonomic units (OTUs) were produced under the threshold of 99%. 

The raw data from the UPLC-Q/TOF-MS/MS system was pre-processed with MarkerLynx (v4.1) software for alignment, deconvolution, and reduction as previously described by our group [28]. The metabolomics data were exported for further multivariate analysis.

### 4.10. Bioinformatic and Statistical Analysis

OTU table was imported into the MicrobiomeAnalyst (v4.0) for ecological analysis and the identification of the taxonomic units [74]. The filtering parameters were set as default. The quality of sequencing tags was displayed with QIIME2. α-Diversity indexes were also calculated with QIIME2 software. The cladogram relationships of the different taxonomic units were plotted using the LEfse platform [75]. The metabolic function of the microbiota was predicted with Phylogenetic Investigation of Communities by Reconstruction of Unobserved States (PICRUSt) based on the filtered high-quality reads of the microbiota [76] in level 3.

Multivariate analysis was performed with SIMCA-P software (v14.1, Umetric, Umeå, Sweden) using the data exported above. Establishment of the PCA and OPLS-DA models was the same as in our previous report [28]. Variable importance of project (VIP) values and the statistical significance of the metabolites were used to identifiy the markedly perturbed metabolites (metabolites with VIP > 1.0 and *p* < 0.05 were considered as significant). All distinct metabolites were identified by matching the accurate mass of the fragmentation with the Human Metabolome Database (HMDB Version 4.0) [77] and METLIN database [78]. Metabolic pathways were analyzed with MetaboAnalyst platform (v4.0) based on identified metabolites [79].

The gene data (KEGG orthology format) predicted by PICRUSt and the distinct metabolites (HMDB format) were imported into the Network Explorer module of MetaboAnalyst platform (v4.0) for joint multiomics analysis. The interactions between metabolites and diseases, between metabolites and microbiota genes, and among the metabolites, genes, and diseases were each analyzed. The comprehensive pharmacological network, which involved the immune and multiomics mechanism, was constructed with Cytoscape (v3.6.5) [80].

The statistical method was as previously described [28,72]. Statistical significance was accepted when *p* < 0.05. The statistical significance was evaluated with R (v3.3.3) basic statistical package, and the bar charts were plotted with ggplot2 (v2.2.1) package which is based on R software [81].

## 5. Conclusions

In conclusion, we demonstrated that RWC could alleviate the AHR of NEA in a phenotype-specific manner. Combined treatment with DEX improved the AHR of asthmatic phenotypes, the dynamic compliance of EA, and the resistance to steroid. RWC combined with DEX plays a role in improving spirometry results and inhibiting airway inflammation through phenotype-specific regulation of the imbalance of Th2/Th1, decreasing Th17, enhancing Tregs, and suppressing the secretion of related inflammatory mediators. The combined treatment changed not only the taxonomic composition of the microbiota in NEA more significantly than EA, but also the systematic and pulmonary metabolic profiles. The changed structure of the microbiota in NEA after the combined treatment may be related to the steroid resistance and the susceptibility of the asthmatic phenotype, which might involve the direct perturbation of systematic or pulmonary metabolism.

## Figures and Tables

**Table 1:** Different species using LEfse in NEA.

Species	*P* Values	FDR	ND	NDR	NEA	NR	LDA Score
*Proteus mirabilis*	6.44 × 10^−4^	0.036724	116,110	162,152	14,237	9937	4.88
*Lactobacillus hamsteri*	0.0014144	0.040311	108,397	73,790	3508	3417	4.72

**Table 2:** Distinct metabolites identified in serum and BALF samples.

M	Mass	RT	Compound Name	KEGG_ID	Δm	Pathways	Sample
1	327.2008	2.04	Hydroxydehydroepiandrosterone/ Hydroxytestosterone	C18045/C05139/C05291/C05294	2	Steroid hormone biosynthesis	Serum
2	327.2008	2.04	LysoPC(18:3(9Z,12Z,15Z))	C04230	2	Glycerophospholipid metabolism	Serum
3	371.2275	3.02	Dihydroxypregnenolone	C05487/C05478	0	Steroid hormone biosynthesis	Serum
4	248.0278	5.67	Pyridoxal 5’-phosphate	C00018	0	Vitamin B6 metabolism	Serum
5	745.416	6.11	Octaprenyl diphosphate	C04146	4	Terpenoid backbone biosynthesis	Serum
6	435.2578	6.87	LysoPA (18:2(9Z,12Z)/0:0)	C00416	17	Glycerophospholipid metabolism	Serum
7	465.247	6.87	Testosterone glucuronide	C11134	2	Steroid hormone biosynthesis	Serum
8	655.3898	8.1	All-*trans*-heptaprenyl diphosphate	C04216	2	Terpenoid backbone biosynthesis	Serum
9	339.1873	8.12	Hydroxyretinoic/Epoxyretinoic acid/Deoxy-PGJ2	C16677/C16679/C16680/C14717	17	Retinol metabolism & Arachidonic acid	Serum
10	361.2005	8.29	Cortisone/Aldosterone	C00762/C01780	1	Steroid hormone biosynthesis	Serum
11	318.3007	12.79	Phytosphingosine	C12144	1	Sphingolipid metabolism	Serum
12	302.3065	14.99	Sphinganine	C00836	4	Sphingolipid metabolism	BALF
13	303.2323	18.12	Bovinic acid/Linoleic acid/Retinyl ester	C04056	1	Linoleic acid metabolism	Serum
14	298.1327	18.42	Phenethylamine glucuronide	C03033	13	Starch and sucrose metabolism	BALF
15	349.2352	19.13	Dihydroxypregnanedione	C05478	1	Steroid hormone biosynthesis	Serum
16	626.2956	19.19	Leukotriene C4	C02166	20	Arachidonic acid metabolism	Serum
17	609.3009	20.04	All-*trans*-hexaprenyl diphosphate	C01230	6	Terpenoid backbone biosynthesis	Serum
18	499.2297	20.3	Retinoyl b-glucuronide	C11061	1	Retinol metabolism	Serum
19	450.3213	22.01	Chenodeoxycholic acid glycine conjugate	C05466	0	Primary bile acid biosynthesis	BALF
20	265.2516	22.01	1-Hexadecanol	C00823	4	Fatty acid metabolism	BALF
21	433.2227	23.08	LysoPA (16:0/0:0)	C00416	1	Glycerophospholipid metabolism	BALF
22	555.2908	23.12	5b-Cyprinol sulfate	C05468	4	Primary bile acid biosynthesis	Serum
23	349.2357	23.13	11b,21-Dihydroxy-5b-pregnane-3,20-dione	C05475	2	Steroid hormone biosynthesis	Serum
24	469.1846	23.44	Estrone glucuronide	C11133	2	Steroid hormone biosynthesis	Serum
25	335.2568	24.1	Tetrahydrodeoxycorticosterone	C13713	3	Steroid hormone biosynthesis	Serum
26	742.5645	26.69	PC (18:2(9Z,12Z)/P-16:0)	C00157	13	Glycerophospholipid metabolism	Serum
27	760.5777	27.04	PC (18:1(9Z)/16:0)/PE (22:1(13Z)/15:0)	C00157/C00350	10	Glycerophospholipid metabolism	BALF
28	788.6045	27.06	PC (14:0/22:1(13Z))	C00157	15	Linoleic acid metabolism	Serum
29	798.585	27.1	PE (22:0/16:0)	C00350	17	Glycerophospholipid metabolism	Serum
30	703.5725	27.3	SM( d18:1/16:0)	C00550	3	Sphingolipid metabolism	Serum
31	782.5662	27.56	PC (18:4(6Z,9Z,12Z,15Z)/18:0)/PE (22:1(13Z)/15:0)	C00157/C00350	1	Glycerophospholipid metabolism	BALF
32	732.5517	27.75	PE (15:0/20:1(11Z))	C00350	3	Glycerophospholipid metabolism	BALF
33	349.2334	28.62	Dihydroxypregnanedione	C05475/C05478/C05487	4	Steroid hormone biosynthesis	BALF
34	425.341	28.7	Hydroxycholesterol	C05500/C05502/C15519/C03594/ C13550/C05451	0	Steroid hormone & Primary bile acid biosynthesis	Serum

M, metabolite number; RT, retention time; Δ, relative deviation (ppm); Sample, sample type; Mass, *m/z*.

**Table 3:** Administration method of all mice.

Groups	Treatments	Routes of Administration	Number (*n*)
Control	N/A	N/A	18
EA	NS	i.g	18
	RWC	i.g	18
	DEX	i.p	18
	RWC + DEX	i.g + i.p	18
NEA	NS	i.g	18
	RWC	i.g	18
	DEX	i.p	18
	RWC + DEX	i.g + i.p	18

NS: normal saline; i.g: intragastric administration; i.p: intraperitoneal administration.

## Supplementary Materials

Supplementary materials can be found at https://www.mdpi.com/1422-0067/20/17/4216/s1.

## Abbreviations

AHR	airway hyperresponsiveness
AR	analytical reagent
BALF	bronchoalveolar lavage fluid
Cdyn	dynamic compliance
CFA	complete Freund’s adjuvant
DEX	dexamethasone
EA	eosinophilic asthma
ED	eosinophilic asthma treated with dexamethasone
ER	eosinophilic asthma treated with *Rhodiola wallichiana* var. *cholaensis*
ERD	eosinophilic asthma treated with *Rhodiola wallichiana* var. *cholaensis* and dexamethasone
FDR	false discovery rate
FEV0.1	forced expiratory volume in 0.1 s
FRC	functional residual capacity
HC	healthy control
HPLC	high-performance liquid chromatography
H&E	hematoxylin and eosin
KEGG	Kyoto Encyclopedia of Genes and Genomes
KO	KEGG orthology
LDA	linear discriminant analysis
LEFse	linear discriminant analysis effect size
NEA	non-eosinophilic asthma
ND	non-eosinophilic asthma treated with dexamethasone
NMDS	non-metric multidimensional scaling
NR	non-eosinophilic asthma treated with *Rhodiola wallichiana* var. *cholaensis*
NRD	non-eosinophilic asthma treated with *Rhodiola wallichiana* var. *cholaensis* and dexamethasone
OBS	observed species
OPLS-DA	orthogonal projections to latent structures discriminant analysis
OTU	operational taxonomic units
OVA	ovalbumin
PCA	principal component analysis
PCoA	principal coordinates analysis
PD	phylogenetic diversity
PICRUSt	Phylogenetic Investigation of Communities by Reconstruction of Unobserved States
PMA	phorbol-12-myristate-13-acetate
QC	quality control
Q/TOF-MS	quadrupole time-of-flight mass spectrometry
RI	resistance of inspiration
RSD	relative standard deviation
RT	retention time
RWC	Rhodiola wallichiana var. cholaensis
SPF	specific pathogen-free
Tregs	regulatory T cells
UPLC	ultra-performance liquid chromatography
