# Supplementary material for: Phenotype-Specific Therapeutic Effect of Rhodiola wallichiana var. cholaensis Combined with Dexamethasone on Experimental Murine Asthma and Its Comprehensive Pharmacological Mechanism
Source: Int J Mol Sci. 2019 Aug 28;20(17):4216. doi: 10.3390/ijms20174216 (PMC6747379; doi:10.3390/ijms20174216)
Supplement: Supplementary file 1 [file ijms-20-04216-s001.pdf]

Supplementary Materials

Phenotype-specific Therapeutic Effect of *Rhodiola wallichiana var. cholaensis* Combined with Dexamethasone on Murine Asthma and Its Comprehensive Pharmacological Mechanism

Zhiqiang Pang[1], Nan Ran[1], Yuze Yuan[1], Cuizhu Wang[1,2], Guoqiang Wang[1], Hongqiang Lin[2], Alan Chen-Yu Hsu[3], Jinping Liu[2] and Fang Wang[1, 4*]

* Correspondence: wf@jlu.edu.cn; Tel.: +86-431-85649509 (F.W.)

Table S1. Chromatographic Gradient Condition Parameters

Time Points	Mobile Phase A/%	Mobile Phase B/%	Flow Rate (mL/min)
0.01min	9	91	0.5
24min	20	80	0.5
42min	22	78	0.7
60min	30	70	1.0
60.01min	9	91	1.0
75min	9	91	1.0

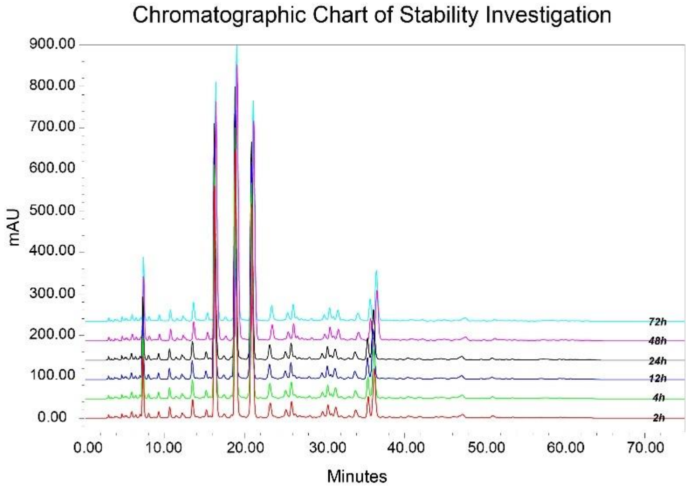

Figure S1. Chromatographic chart of the stability investigation at different time points (including 2h, 4h, 12h, 24h, 48h and 72h).

Table S2. Results of the Investigation on Stability

Time Points	Retention Time	Peak area	Percentage of Peak Area (%)	Peak Hight
2h	16.26	9354284	20.40	558902
4h	16.22	9431619	20.33	562594
12h	16.19	9405484	20.43	563594
24h	16.22	9512479	20.34	568769
48h	16.42	9328926	20.26	554830
72h	16.39	9603952	20.18	566178
Average	16.28	9439457.3	20.32	562477.83
RSD%	0.60	1.1	0.45	0.89

Supplementary Materials

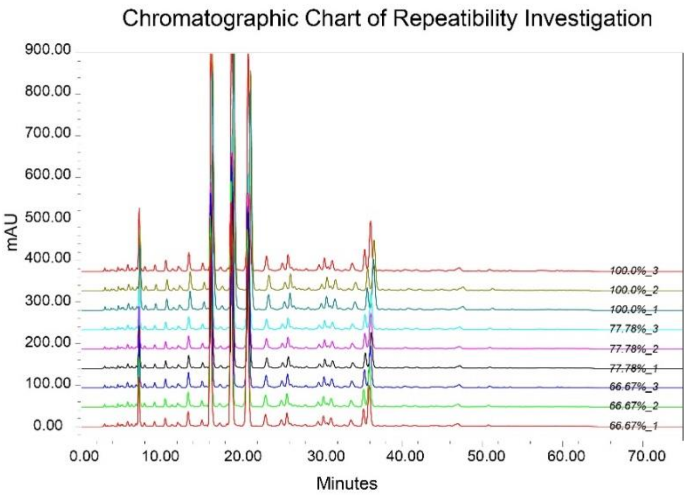

Figure S2. Chromatographic chart of the repeatability investigation at different relative concentrations (including 66.67%, 77.78% and 100%).

Table S3. Results of the Investigation on Repeatability

NO.		Retention Time	Peak area	Percentage of Peak Area	Peak Hight
Percentage of Conc	Times				
66.67%	1	16.25	6569928	20.10	401205
	2	16.23	6513661	20.03	399665
	3	16.23	6453186	20.06	396129
Average		16.2	6512258.3	20.1	398999.7
RSD%		0.06	0.90	0.18	0.65
77.78%	1	16.14	7464159	20.17	455557
	2	16.13	7520086	19.99	458753
	3	16.17	7693697	20.08	467769
Average		16.1	7559314.0	20.1	460693.0
RSD%		0.14	1.58	0.45	1.37
100%	1	16.39	9703952	20.18	576178
	2	16.42	9728926	20.26	574830
	3	16.22	9512479	20.34	568769
Average		16.3	9648452.3	20.3	573259.0
RSD%		0.66	1.23	0.39	0.69

Conc: concentration.

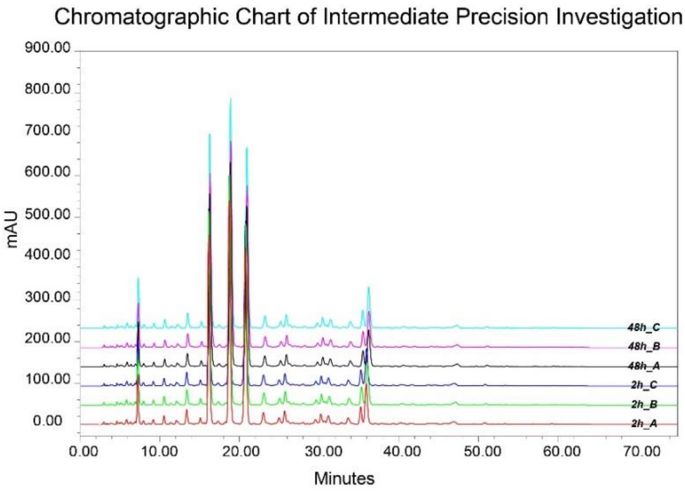

Figure S3. Chromatographic chart of the investigation on intermediate

precision of testing samples of different operators (A/B/C) in different timepoints (2h and 48h).

Table S4. Results of the Investigation on Intermediate Precision

NO.		Retention Time	Peak area	Percentage of Peak Area	Peak Hight
Time	Operators				
2h	A	16.39	9603952	20.18	569178
	B	16.22	9512479	20.34	568769
	C	16.22	9431619	20.33	562594
48h	A	16.26	9239270	20.46	547790
	B	16.25	9393182	20.14	553898
	C	16.26	9148635	20.41	541054
Average		16.27	9388189.5	20.31	557213.83
RSD%		0.40	1.8	0.62	2.07

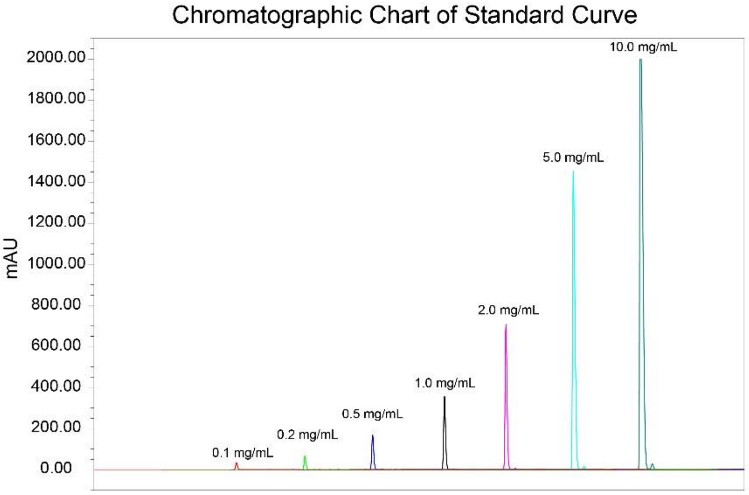

Figure S4. Chromatographic chart of the investigation on the linear at different concentrations (0.1, 0.2, 0.5, 1.0, 2.0, 5.0 and 10.0mg/mL).

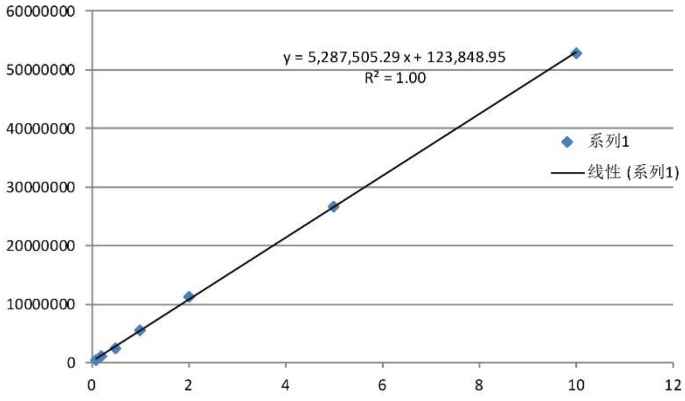

Figure S5. Regression equation of the investigation on the linearity at different concentrations (0.1, 0.2, 0.5, 1.0, 2.0, 5.0 and 10.0mg/mL).

Table S5 Results of the Investigation on Linear

NO_Conc(mg/mL)		A_0.1	B_0.2	C_0.5	D_1.0	E_2.0	F_5.0	G_10.0
PA	1	526321	1038457	2575931	5580350	11010583	26625171	53270040
	2	519505	1045373	2583733	5581070	11304394	26493581	52841399
	3	520110	1028414	2325293	5368352	11357070	26688486	52532493
	A	521978	1037414	2494985	5509924	11224015	26602412	52881310
PH	1	35542	69036	169454	357465	711548	1454640	2004293
	2	35152	69415	169702	353777	709279	1449428	2003690
	3	34892	68002	152866	346635	710000	1453506	2004218
	A	35195	68818	164007	352626	710276	1452525	2004067

PA: Peak Area; PH: Peak Height; A: Average; Conc: Concentration.

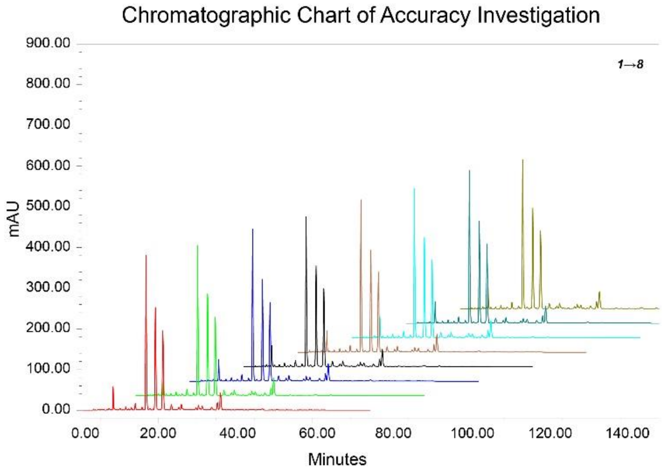

Figure S6. Chromatographic chart of the investigation on the accuracy based on Ratio of Recovery.

Table S6. Results of the Investigation on Accuracy

NO.	Retention Time	Peak area	Precentage of Peak Area (%)	Peak Hight	Concentration(mg/mL)	Ratio of Recovery (%)
1	16.30	5939234	30.59	366492	1.10	99.17
2	16.50	5908688	31.24	375670	1.09	98.01
3	16.23	5897949	31.26	366193	1.09	97.61
4	16.40	5898729	31.44	375312	1.09	97.63
5	16.19	5924084	31.26	367832	1.10	98.59
6	16.33	5937088	31.39	373332	1.10	99.09
7	16.09	5898992	31.14	369059	1.09	97.64
8	16.71	5936974	31.33	381407	1.10	99.08
Average	16.35	5917717.3	31.2	371912.1	1.1	98.4
RSD%	1.19	0.3	0.9	1.5	0.3	0.7

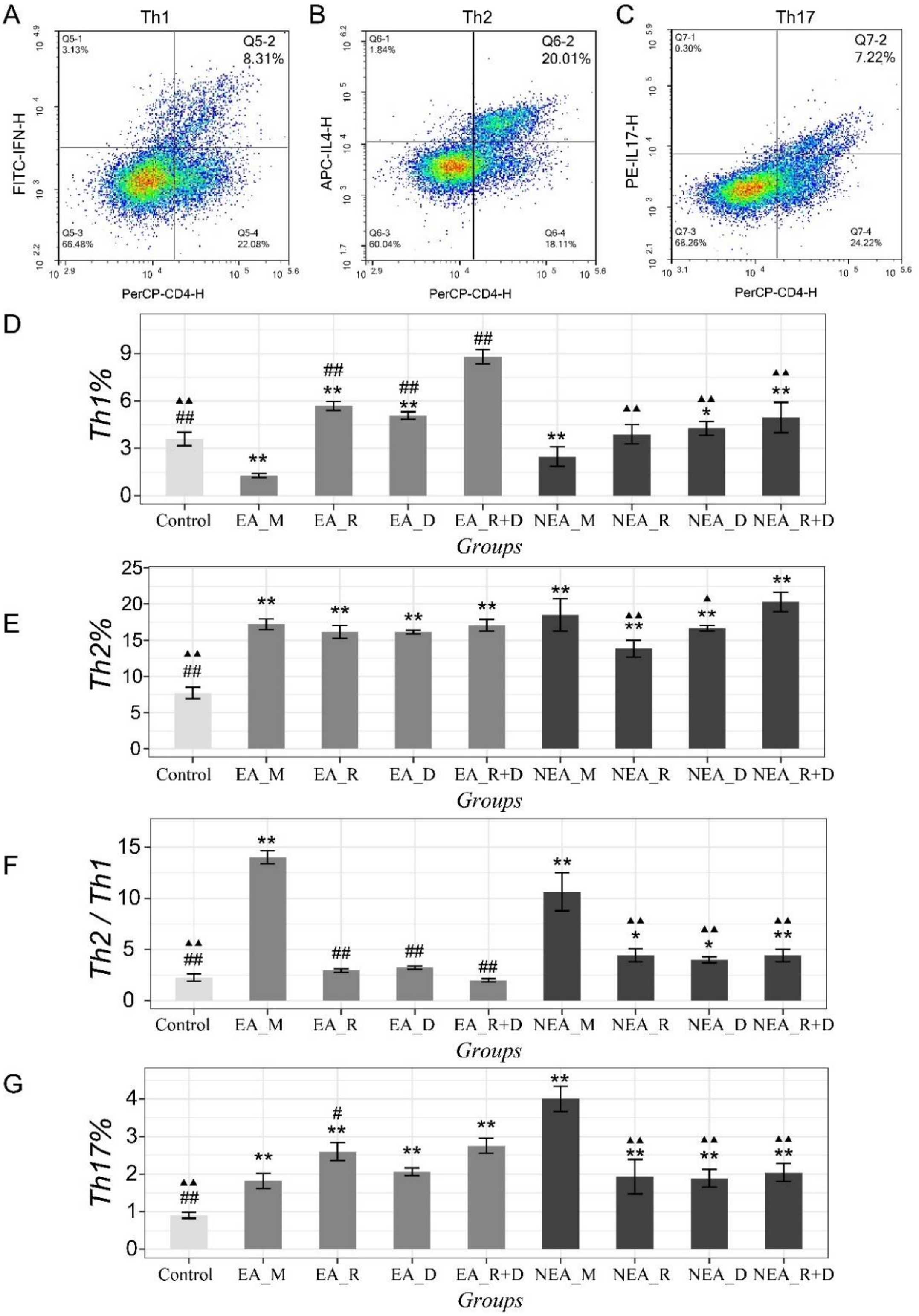

Figure S7. Typical flow cytometry dot plots of helper T cell clusters (including Th1/Th2/Th17, from one of the samples of all groups). (**A**) Th1 (CD4+-IFN-γ+) in spleen tissue; (**B**) Th2 (CD4+-IL-4+) in spleen tissue; (**C**) Th17 (CD4+-IL-7+) in spleen tissue. The statistical data of Th1 cells (**D**) Th2 cells (**E**) Th2/Th1 ratio (**F**) and Th17 cells (**G**) in mice spleen tissues with flowcytometry technique. * $p < 0.05$ vs. HC； ** $p < 0.01$ vs. HC; ## $p < 0.01$ vs. EA； # $p < 0.05$ vs. EA; ▲▲ $p < 0.01$ vs. NEA.

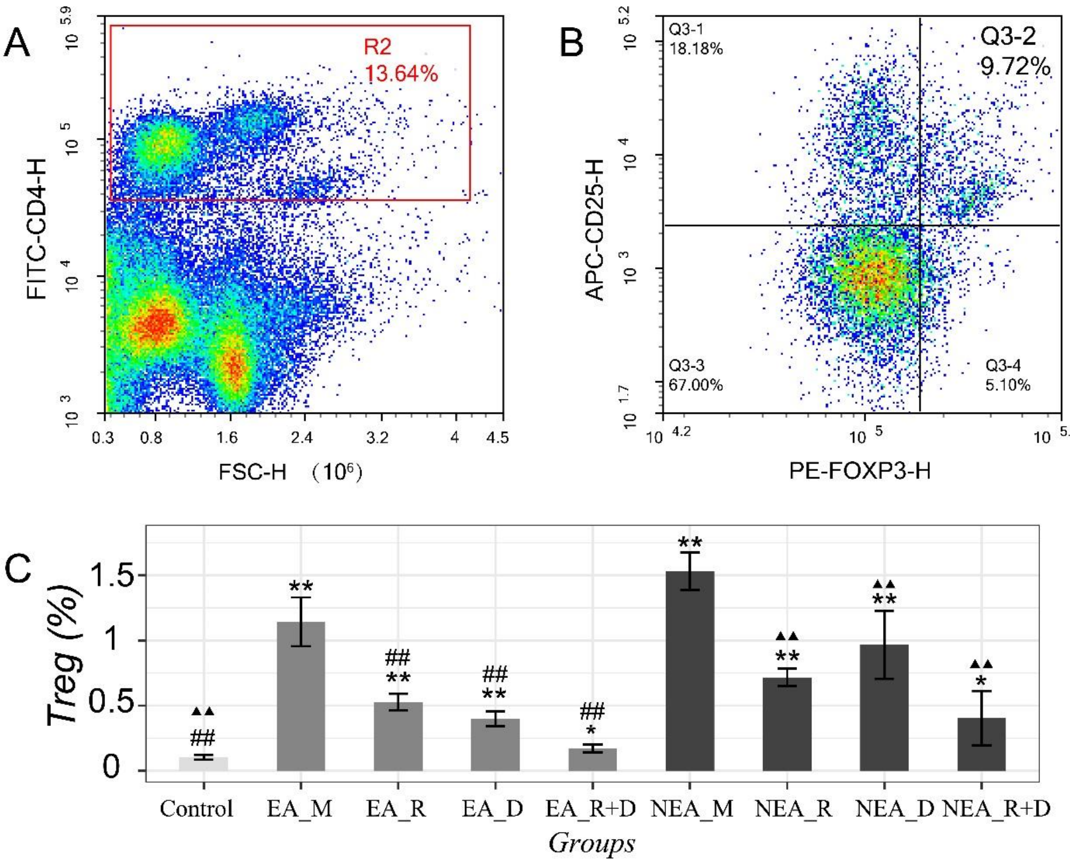

Figure S8. Typical flow cytometry dot plots of regulatory T cell clusters. (**A**) CD4 positive and negative cells in spleen; (**B**) Treg cells (CD4+-CD25+-Foxp3+) in spleen. The statistical data of Tregs (**C**) in mice spleen tissues with flowcytometry technique. * $p < 0.05$ vs. HC； ** $p < 0.01$ vs. HC; ## $p < 0.01$ vs. EA； # $p < 0.05$ vs. EA; ▲ $p < 0.05$ vs. NEA; ▲▲ $p < 0.01$ vs. NEA.

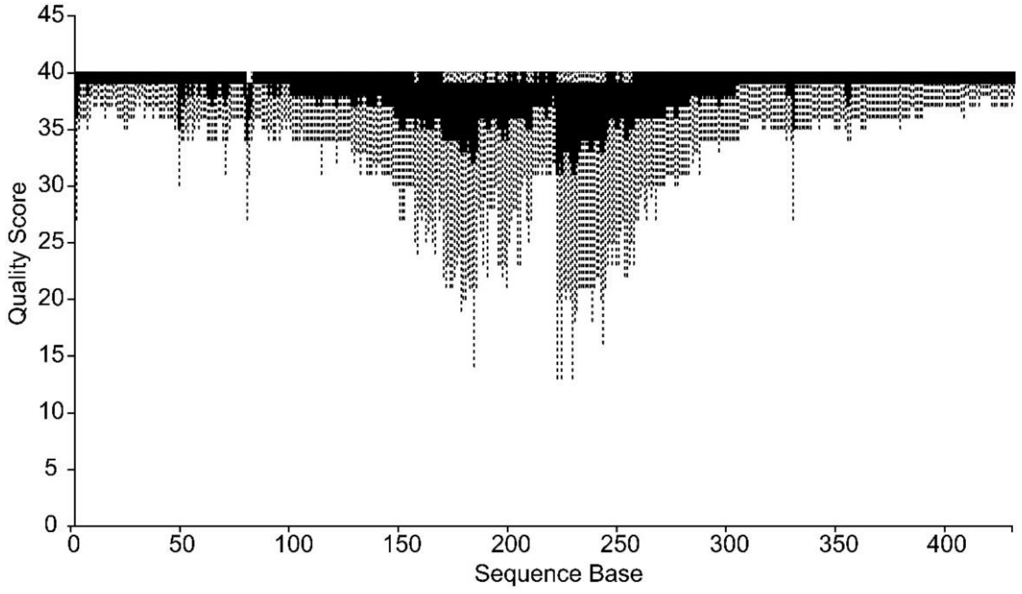

Figure S9. Statistical results of sequencing data from Hiseq sequencing platform after filtering. Quality score is the QC index.

Table S7. Statistic Results of OBS Index with Kruskal-Wallis (Pairwise)

Group 1	Group 2	H	*P*-value	Q-value
Control (n=8)	EA (n=6)	7.4105	0.006484	0.035663
Control (n=8)	ED (n=6)	9.6211	0.000923	0.013224
Control (n=8)	ER (n=8)	9.6211	0.001923	0.013224
Control (n=8)	EDR (n=8)	9.6211	0.001923	0.013224
Control (n=8)	NEA (n=8)	7.4105	0.006484	0.035663
Control (n=8)	ND (n=8)	9.7808	0.001763	0.013224
Control (n=8)	NR (n=8)	10.5187	0.001181	0.013224
Control (n=8)	NDR(n=7)	9.6211	0.001923	0.013224
ED (n=6)	ND (n=8)	8.5918	0.002124	0.035663
EDR (n=8)	ND (n=8)	4	0.045500	0.192501
ER (n=8)	ND (n=8)	5.224489796	0.022270	0.111354
ER (n=8)	NDR (n=7)	3.448979592	0.063290	0.248642

Table S8. Statistic Results of Shannon Index with Kruskal-Wallis (Pairwise)

Group 1	Group 2	H	*P*-value	Q-value
Control (n=8)	EA (n=6)	5.653846	0.017417	0.079829
Control (n=8)	ED (n=6)	9.6	0.001946	0.015288
Control (n=8)	ER (n=6)	9.6	0.001946	0.015288
Control (n=8)	EDR (n=6)	9.6	0.001946	0.015288
Control (n=8)	NEA (n=6)	8.066667	0.004509	0.030997
Control (n=8)	ND (n=8)	10.5	0.001194	0.015288
Control (n=8)	NR (n=8)	10.5	0.001194	0.015288
Control (n=8)	NDR (n=7)	7.384615	0.006578	0.040201
ED (n=6)	ND (n=8)	3.44898	0.033291	0.219723
ER (n=8)	ND (n=8	5.897959	0.015158	0.075792
EDR (n=8)	ND (n=8)	6.612245	0.010128	0.055704
ND (n=8)	NDR (n=7)	3.571429	0.048782	0.219723

Table S9. Statistic Results of Pielou Evenness Index with Kruskal-Wallis (Pairwise)

Group 1	Group 2	H	*P*-value	Q-value
Control (n=8)	ED (n=6)	9.6	0.001945	0.01783
Control (n=8)	EDR (n=8)	9.6	0.001945	0.01783
Control (n=8)	ER (n=8)	8.0666	0.004508	0.03099
Control (n=8)	ND (n=8)	10.5	0.001193	0.01783
Control (n=8)	NDR (n=7)	10.5	0.001193	0.01783
Control (n=8)	NEA (n=8)	7.3846	0.006578	0.04020
Control (n=8)	NR (n=8)	9.0535	0.002621	0.02060
EDR (n=8)	ND (n=8)	5.2244	0.022270	0.12249

Table S10. Statistic Results of Faith PD Index with Kruskal-Wallis (Pairwise)

Group 1	Group 2	H	*P*-value	Q-value
Control (n=8)	EA (n=6)	4.875	0.02724	0.13945
Control (n=8)	ED (n=6)	9.6	0.00194	0.03567
Control (n=8)	ER (n=8)	9.6	0.00194	0.03567
Control (n=8)	EDR(n=8)	9.6	0.00194	0.03567
Control (n=8)	NEA (n=8)	8.06666	0.00450	0.04959
Control (n=8)	ND (n=8)	4.83482	0.02789	0.13945
Control (n=8)	NR (n=8)	6.48214	0.01089	0.07491
Control (n=8)	NDR (n=7)	8.06666	0.00450	0.04959
EA (n=6)	EDR (n=8)	4.54545	0.03300	0.03567
ED (n=6)	EDR (n=8)	5.02564	0.02497	0.03567
ER (n=8)	EDR (n=8)	4.33333	0.03737	0.03567
EDR (n=8)	NEA (n=8)	4.33333	0.03737	0.03567
EDR (n=8)	NDR (n=7)	3.44897	0.04329	0.03567

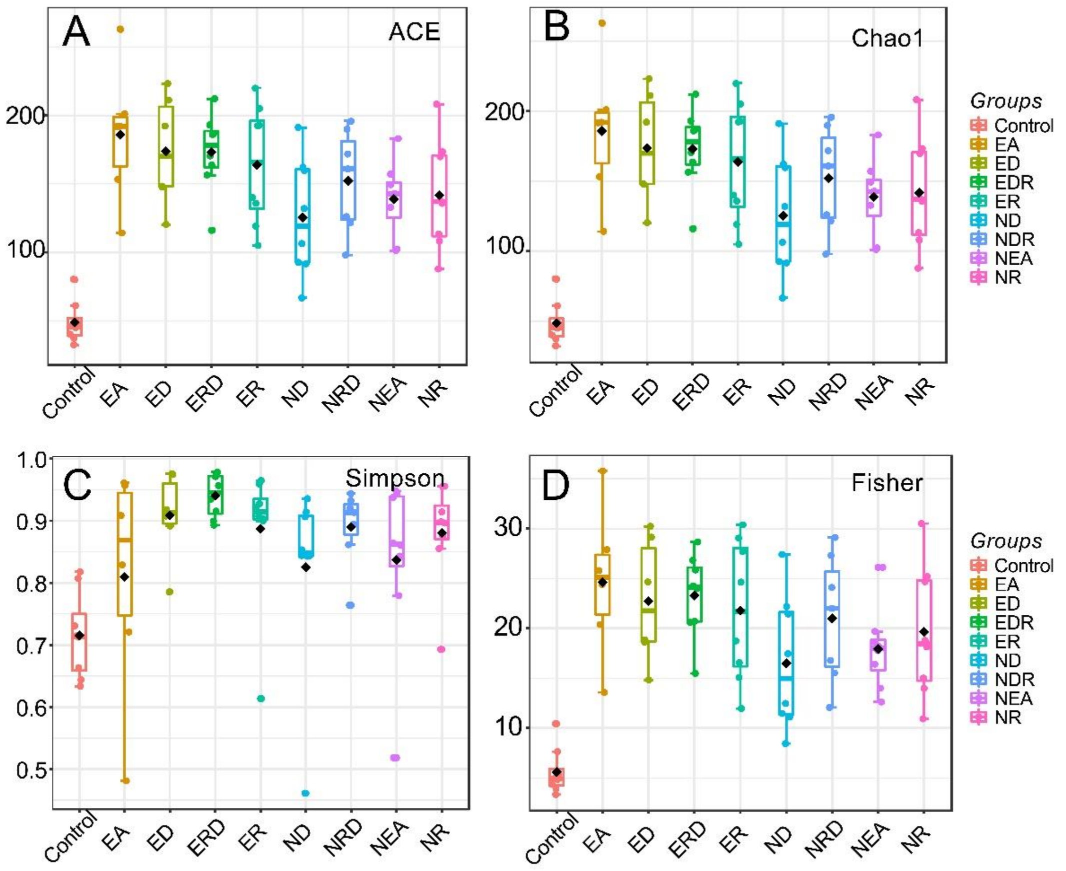

Figure S10. The box plots of α-diversity indexes, including (A) ACE, (B) Chao1, (C) Simpson and (D) Fisher.

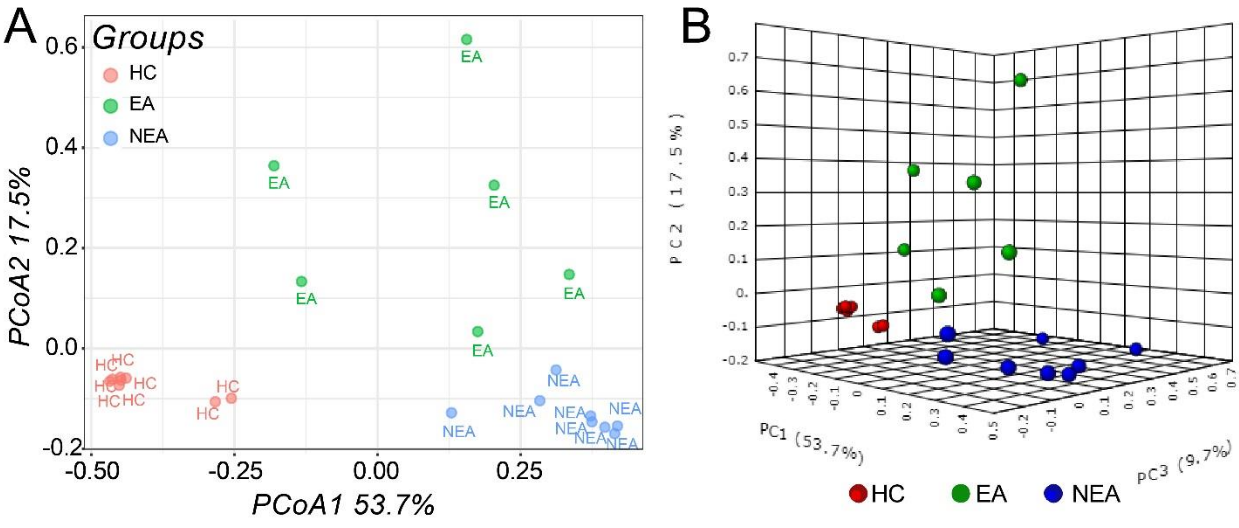

Figure S11. PCoA plots (PCoA1 = 53.7%, PCoA2 = 17.5%, PCoA3 = 9.7%) of Models and HC in (A) 2-demention (2D) and in (B) 3-demention (3D)

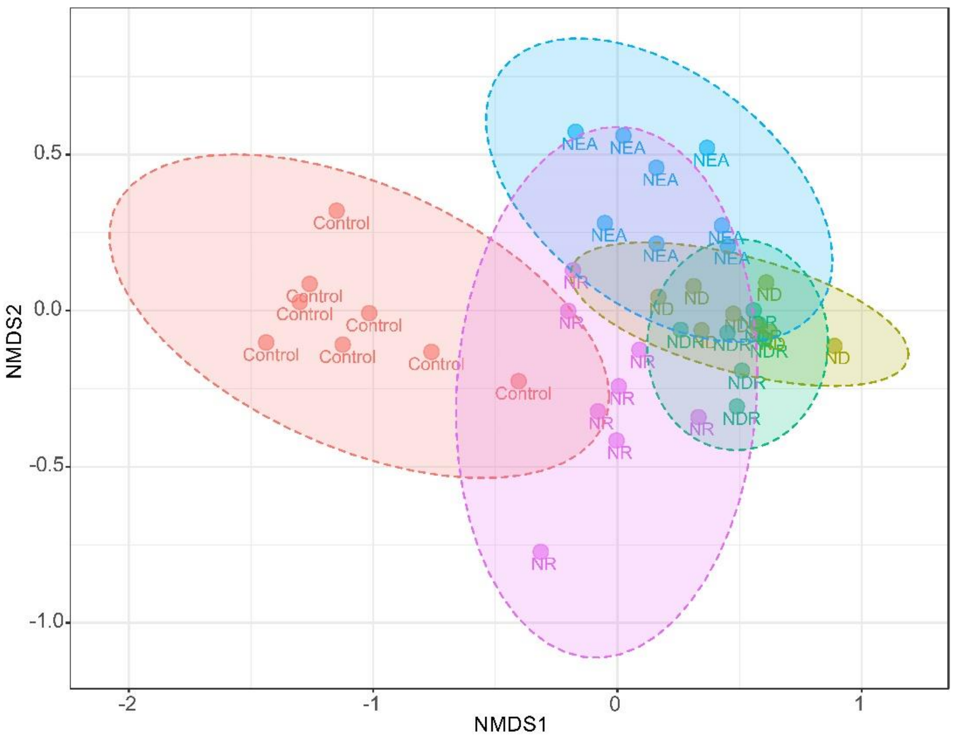

Figure S12. Results of NMDS β-Diversity Analysis (NEA Model and Therapeutic groups).

Table S11. Results of different genera in Model group based on LEfse analysis

Genera	*P* values	FDR	Control	EA	NEA	LDA score
Rahnella	8.97E-05	0.004811	0	3372.846	481880.7	5.38
Streptococcus	9.13E-05	0.004811	5599.431	2516714	107289.7	6.10
Staphylococcus	0.000144	0.004811	0	33216.3	69466.44	4.54
Bifidobacterium	0.000229	0.004811	0	105499.2	64900.57	4.72
Sphingomonas	0.000248	0.004811	3214017	519769.2	38825.21	6.20
Carnobacterium	0.000281	0.004811	0	0	47642.94	4.38
Pseudomonas	0.000281	0.004811	6025.886	83830.22	303817.3	5.17
Propionibacterium	0.000405	0.005664	1856.995	85606.13	277306.1	5.14
Brevundimonas	0.000539	0.005664	1646857	102183.5	23421.32	5.91
Delftia	0.000567	0.005664	320903.2	43345.7	3359.552	5.20
Roseburia	0.000799	0.006572	10337.09	43320.81	144692.1	4.83
Clostridium	0.000829	0.006572	15630.79	367802.9	70113.77	5.25
Bacillus	0.000902	0.006572	0	17483.3	24601.68	4.09
Bacteroides	0.000953	0.006572	7665.216	116664.3	177567.8	4.93
Phyllobacterium	0.001184	0.007029	3613293	1624600	976428.8	6.12
Gemmiger	0.00126	0.007029	1388.58	17218.34	30388.74	4.16
Mitsuokella	0.001269	0.007029	0	0	26087.57	4.12
Acinetobacter	0.001334	0.007029	18522.74	95574.07	106862.8	4.65
Streptomyces	0.001505	0.007222	121.7469	50847.43	21587.42	4.40
Blautia	0.001515	0.007222	6954.13	39689.65	57164.88	4.40
Coprococcus	0.001529	0.007222	2648.514	23727.02	47504.08	4.35
Enhydrobacter	0.001584	0.007234	2496.748	58368.87	33664.13	4.45
Faecalibacterium	0.001747	0.007324	8150.677	93842.84	102597.8	4.67
Weissella	0.001819	0.007324	0	24933.22	3349.114	4.10
Methylobacterium	0.001826	0.007324	3047.311	27097.8	17782.2	4.08
Kaistobacter	0.001871	0.007324	7050.993	49704.91	2000.731	4.38
Dialister	0.00212	0.007946	888.6914	6229.923	29728.92	4.16
Corynebacterium	0.002313	0.007946	1811.961	31256.13	26036.99	4.17
Bradyrhizobium	0.002314	0.007946	1907.687	41505.49	0	4.32
Candidatus_Koribacter	0.002354	0.007946	0	32236.42	4376.954	4.21
Proteus	0.002624	0.00817	0	4272.26	125742.4	4.80
Mycoplana	0.003277	0.009354	2388.358	28531.55	99986.1	4.69
Sutterella	0.004083	0.010553	1518.467	4740.701	30444.51	4.16
Deinococcus	0.004472	0.010906	121.7469	14938.29	24889.43	4.09
Gluconacetobacter	0.004561	0.010906	0	2397.489	30990.22	4.19
Pelomonas	0.004668	0.010906	79044.18	106858	9918.009	4.69
Trabulsiella	0.004822	0.010906	0	3670.514	153269.4	4.88
Paracoccus	0.005175	0.010906	2695.763	22820.96	29693.68	4.13
Succinivibrio	0.005724	0.01181	444.3457	4084.27	154841.6	4.89
Ruminococcus	0.006768	0.013635	18595.68	39567.63	110052.5	4.66
Megasphaera	0.010504	0.019447	499.8889	684.8386	25416.4	4.10
Lactobacillus	0.012897	0.023248	46703.44	244534.5	77310.82	5.00
Rhodoplanes	0.01475	0.025259	4512.635	43625.9	21832.64	4.29
Lachnospira	0.017683	0.029084	4534.365	15492.6	39541.43	4.24
Micrococcus	0.031341	0.044727	1299.603	22712.04	19048.75	4.03

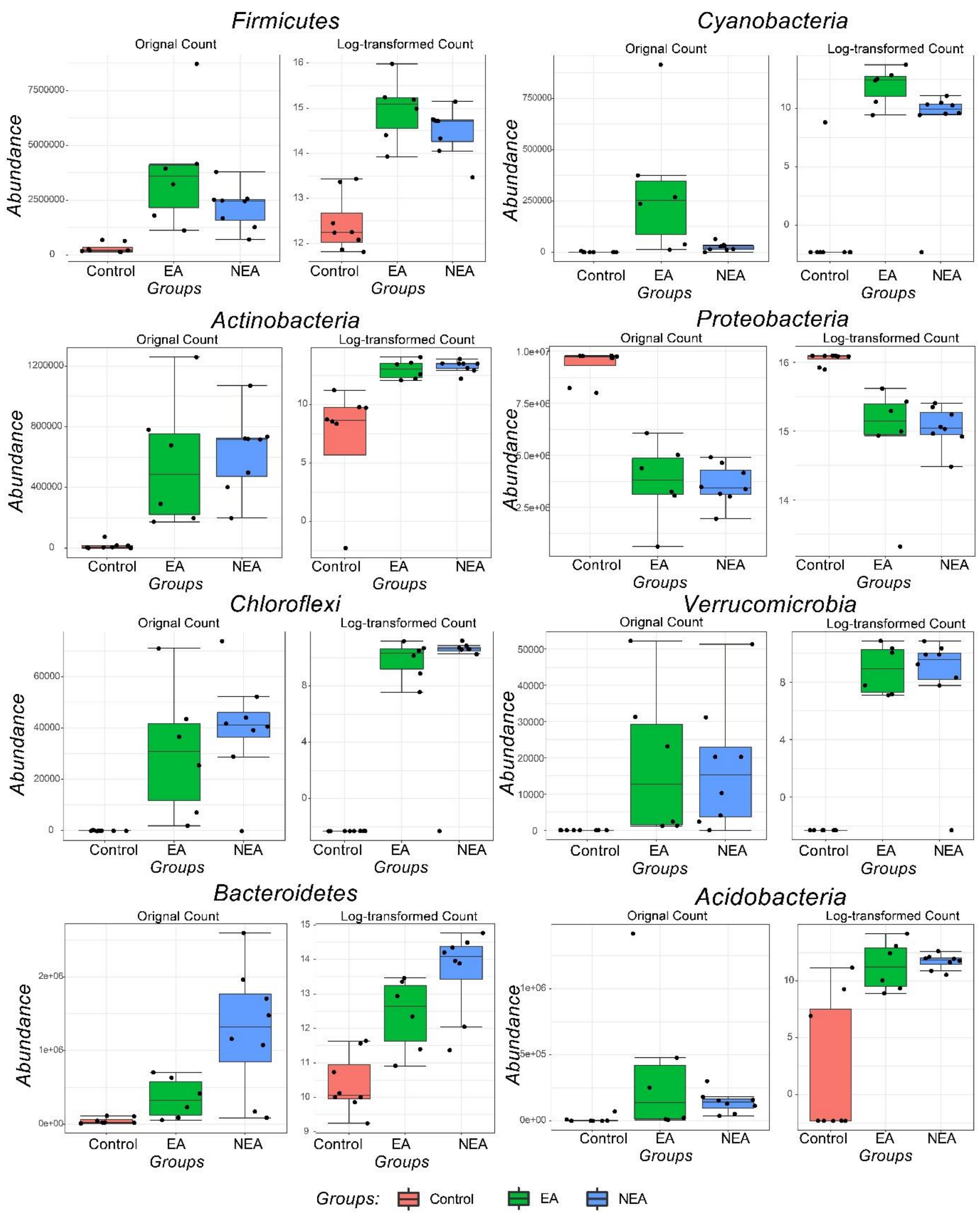

Figure S13. The statistical results of all distinct phyla in all model groups and control group, including the original counts and log-transformed data ($p < 0.05$, LDA>4).

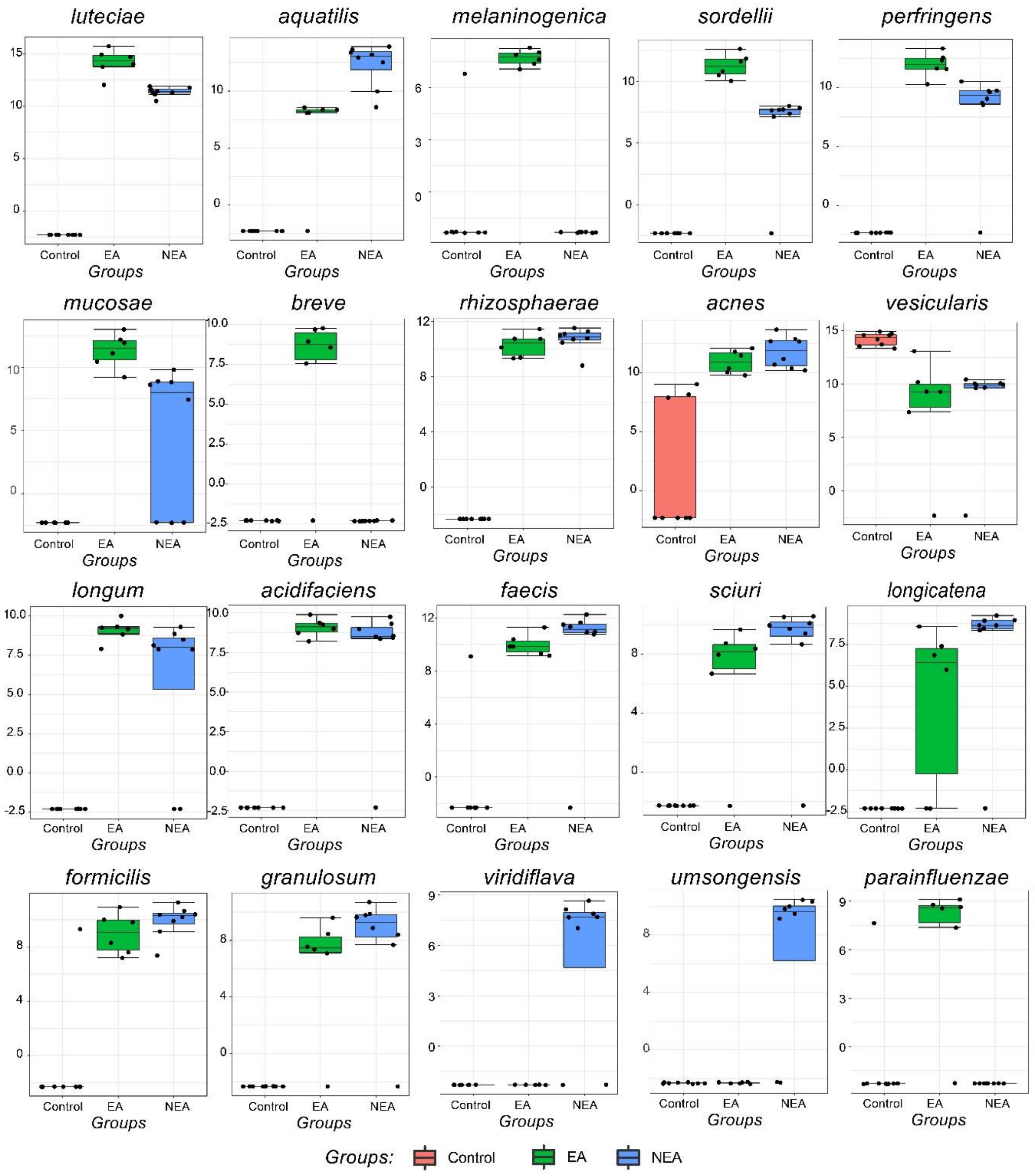

Figure S14. The log-transformed results of distinct species in model groups and control group ($p < 0.05$, LDA>4). All genus species were abbreviated as species and illustrated as follows, *Streptococcus luteciae* (*luteciae*), *Rahnella aquatilis* (*aquatilis*), *Prevotella melaninogenica* (*melaninogenica*), *Clostridium sordellii* (*sordellii*), *Clostridium perfringens* (*perfringens*), *Lactobacillus mucosae* (*mucosae*), *Bifidobacterium breve* (*breve*), *Chryseobacterium rhizosphaerae* (*rhizosphaerae*), *Cutibacterium acnes* (*acnes*), *Brevundimonas vesicularis* (*vesicularis*), *Bifidobacterium longum* (*longum*), *Propionibacterium acidifaciens* (*acidifaciens*), *Bacteroides faecis* (*faecis*), *Staphylococcus sciuri* (*sciuri*), *Dorea longicatena* (*longicatena*), *Gemmiger formicilis* (*formicilis*), *Propionibacterium granulosum* (*granulosum*), *Pseudomonas viridiflava* (*viridiflava*), *Pseudomonas umsongensis* (*umsongensis*) and *Haemophilus parainfluenzae* (*parainfluenzae*).

Table S12. Discrimination results on the stability and durability of the spectrometry and chromatography

Mode	NO	RT/min	m/z	m/z		RT		PA	
				Repeatability (RSD%)	Precision (RSD%)	Repeatability (RSD%)	Precision (RSD%)	Repeatability (RSD%)	Precision (RSD%)
ESI+	1	0.5788	201.6442	0.0006	0.0003	0.0012	0.0000	2.7081	5.2620
	2	12.7089	274.2749	0.0000	0.0001	0.0001	0.0000	1.1709	4.6712
	3	9.7526	338.3431	0.0001	0.0002	0.0215	0.1777	3.1600	2.9135
	4	27.1474	429.2413	0.0001	0.0001	0.1037	0.0554	2.2565	3.1535
	5	20.6052	524.3721	0.0002	0.0002	0.0052	0.0000	3.4421	5.1513
	6	27.6191	613.3402	0.0001	0.0001	0.2473	0.6784	2.1183	5.7450
	7	27.6164	701.3916	0.0000	0.0002	0.1245	0.6980	1.7551	3.8983
	8	27.1398	803.5436	0.0000	0.0001	0.0120	0.0640	2.7071	5.7567
	9	17.9762	953.7809	0.0004	0.0008	0.1020	0.0835	3.9924	2.3065
	10	16.9942	1039.6735	0.0001	0.0004	0.0047	0.0882	1.6940	5.6129

m/z, Mass-to-charge Ratio; RT, Retention Time; PA, Peak Area. NO, Number of the selected ions.

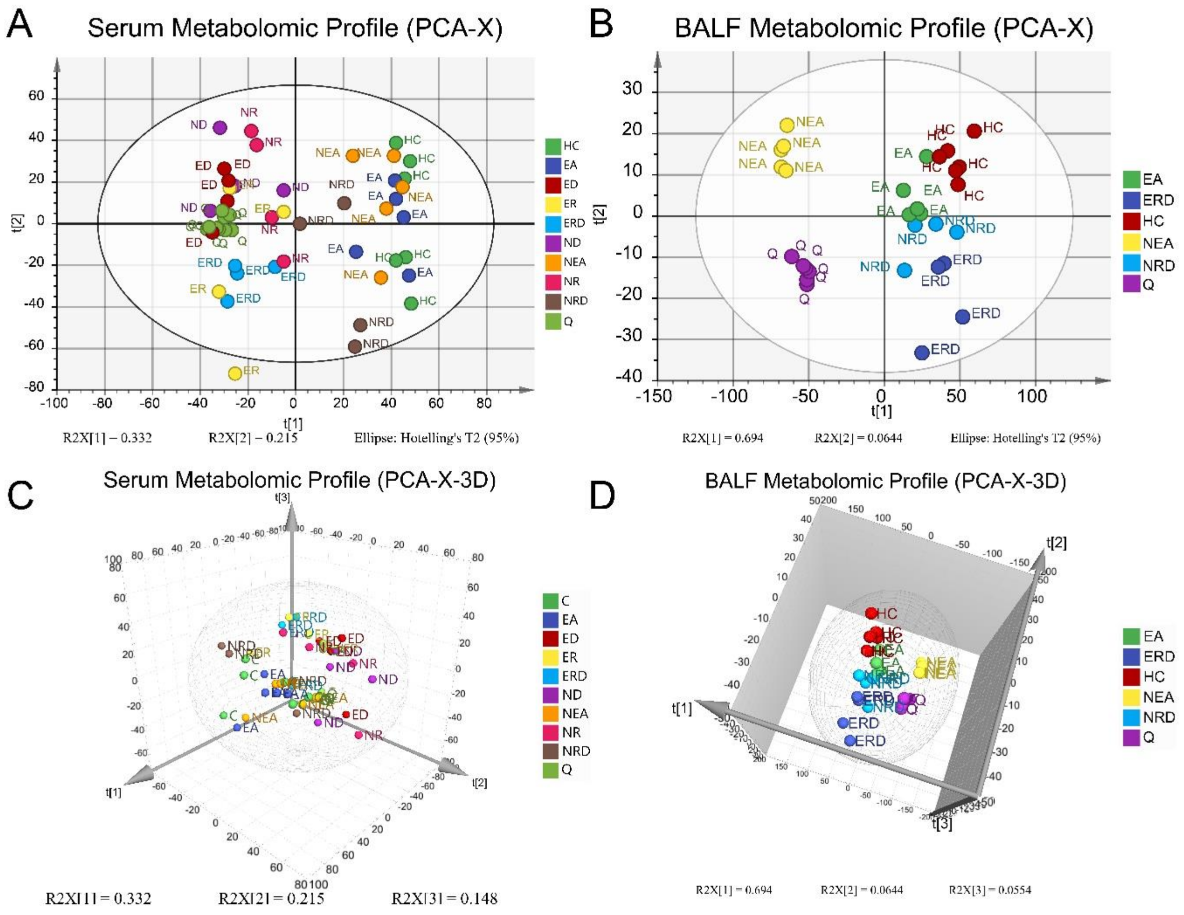

Figure S15. The principal component analysis (PCA) of (**A**) serum metabolomic profile (R2X=0.921， Q2=0.712, PCA1=33.2%, PCA2=21.5%) and its 3D diagram (**C**). PCA of (**B**) BALF metabolomic profile (R2X=0.814, Q2=0.687, PCA1=69.4%, PCA2=6.44%) and its 3D diagram (**D**).

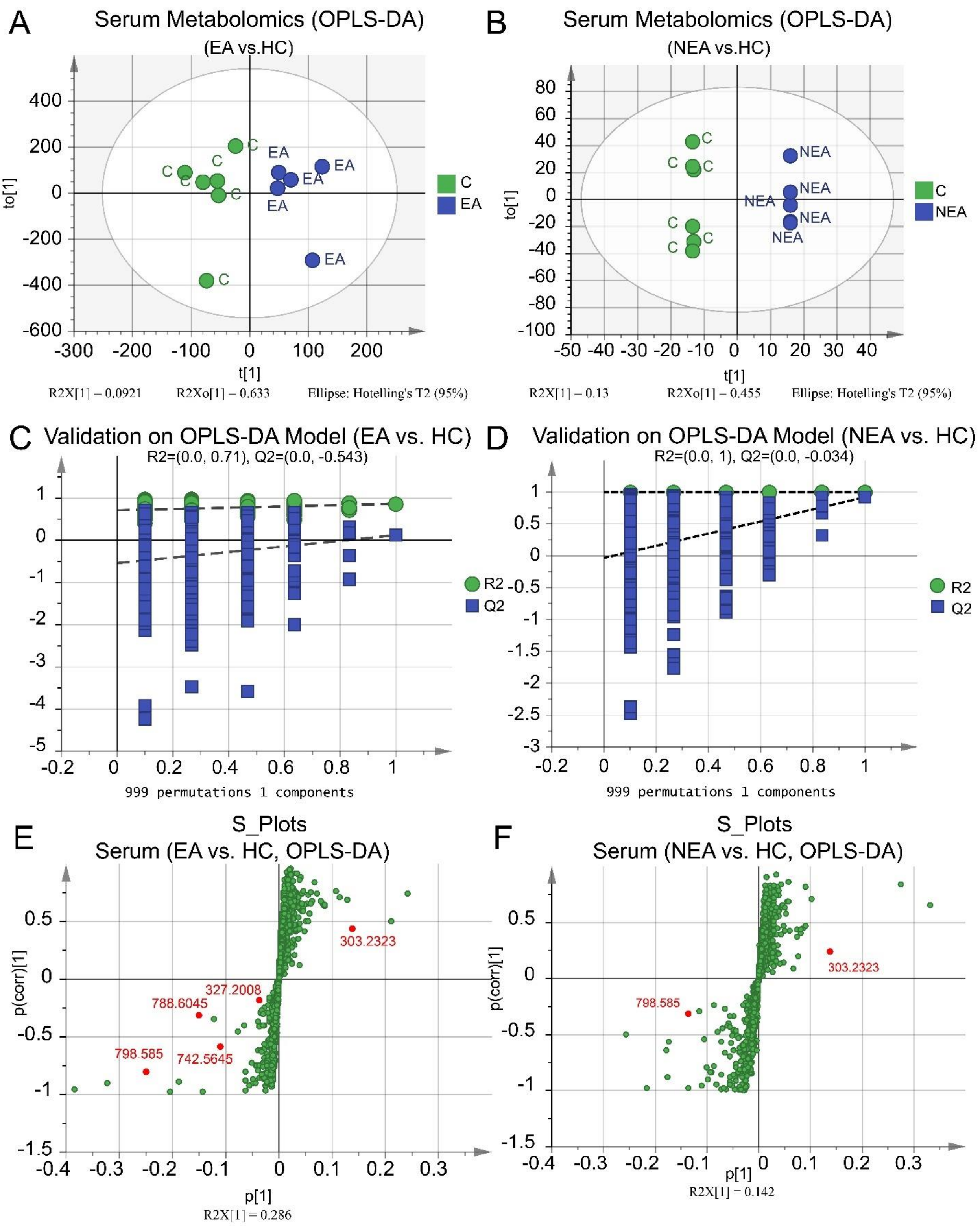

Figure S16. OPLS-DA score plots and their permutation tests of serum metabolomic profile with the metabolites identified displayed in the groups. OPLS-DA models of (A) EA vs. HC, $p < 0.0001$and (B) NEA vs. HC, $p < 0.0001$. The permutation test on the OPLS-DA of (C) EA vs. HC and (D) NEA vs. HC. The S-plots based on OPLS-DA of (E) EA vs. HC and (F) NEA vs. HC and identified metabolites in serum. P values were estimated with CV-ANOVA test. Ellipse in the OPLS-DA panels represents Hotelling's T2 test with the confidence interval of 0.95. The counts of permutation tests are 999.

Supplementary Materials

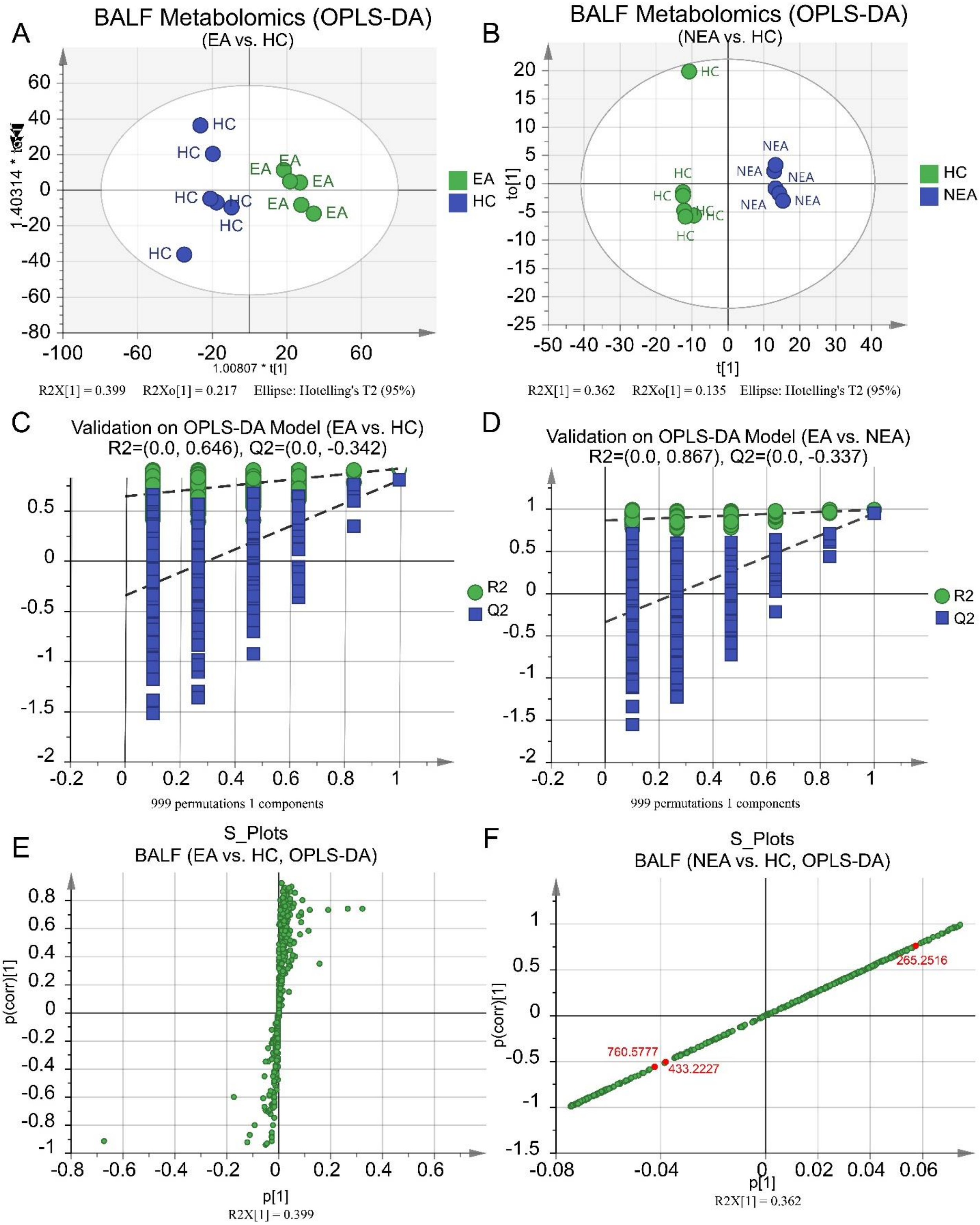

Figure S17. OPLS-DA score plots and their permutation tests of BALF metabolomic profile with the metabolites identified displayed in the groups. OPLS-DA models of (**A**) EA vs. HC, *P*< 0.001and (**B**) NEA vs. HC, *P*< 0.001. The permutation test on the OPLS-DA of (**C**) EA vs. HC and (**D**) NEA vs. HC. The S-plots based on OPLS-DA of (**E**) EA vs. HC and (**F**) NEA vs. HC and identified metabolites in BALF. *P* values were estimated with CV-ANOVA test. Ellipse in the OPLS-DA panels represents Hotelling's T2 test with the confidence interval of 0.95. The counts of permutation tests are 999.

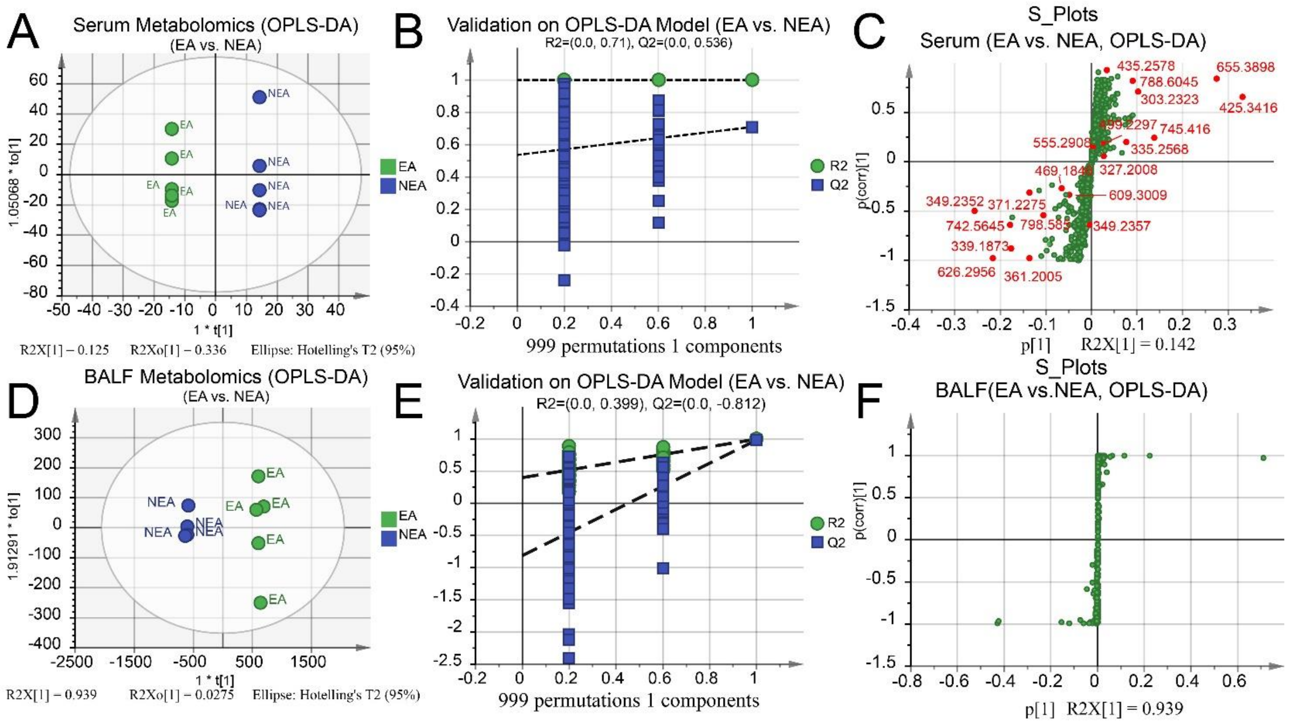

Figure S18. OPLS-DA score plots and their permutation tests of serum and BALF metabolomic profile with the metabolites identified displayed in the groups. OPLS-DA models of (**A**) EA vs. NEA of serum, $p < 0.0001$; (**D**) EA vs. NEA of BALF, $p < 0.0001$. The permutation test on the OPLS-DA of (**B**) EA vs. NEA of serum and (**E**) EA vs. NEA of BALF; The S-plots based on OPLS-DA of (**C**) EA vs. NEA of serum and (**F**)EA vs. NEA of BALF.

Table S13. The Perturbed Metabolic Pathways in Serum and BALF Based on MetaboAnalyst 4.0

Metabolic Pathways	Hits	Raw *P*	=log(*P*)	FDR	Impact	Samples
Steroid hormone biosynthesis	14	6.54E-10	21.148	5.23E-08	0.0793	Serum
Retinol metabolism	5	3.38E-05	10.294	0.001354	0	Serum
Primary bile acid biosynthesis	5	0.001403	6.5691	0.037413	0.11049	Serum
Linoleic acid metabolism	3	0.002225	6.1078	0.044508	0.65625	Serum
Glycerophospholipid metabolism	4	0.005016	5.2951	0.080254	0.33235	Serum
Terpenoid backbone biosynthesis	3	0.021224	3.8526	0.28298	0.11098	Serum
Sphingolipid metabolism	2	0.075136	2.5885	0.8587	0.00954	Serum
Arachidonic acid metabolism	3	0.10232	2.2797	1	0.03338	Serum
Vitamin B6 metabolism	1	0.44802	0.80292	1	0.13651	Serum
Glycerophospholipid metabolism	3	0.000437	7.7366	0.034925	0.32918	BALF
Steroid hormone biosynthesis	3	0.006561	5.0266	0.26245	0.01477	BALF
Linoleic acid metabolism	1	0.06071	2.8017	1	0	BALF
Sphingolipid metabolism	1	0.099319	2.3094	1	0.1402	BALF

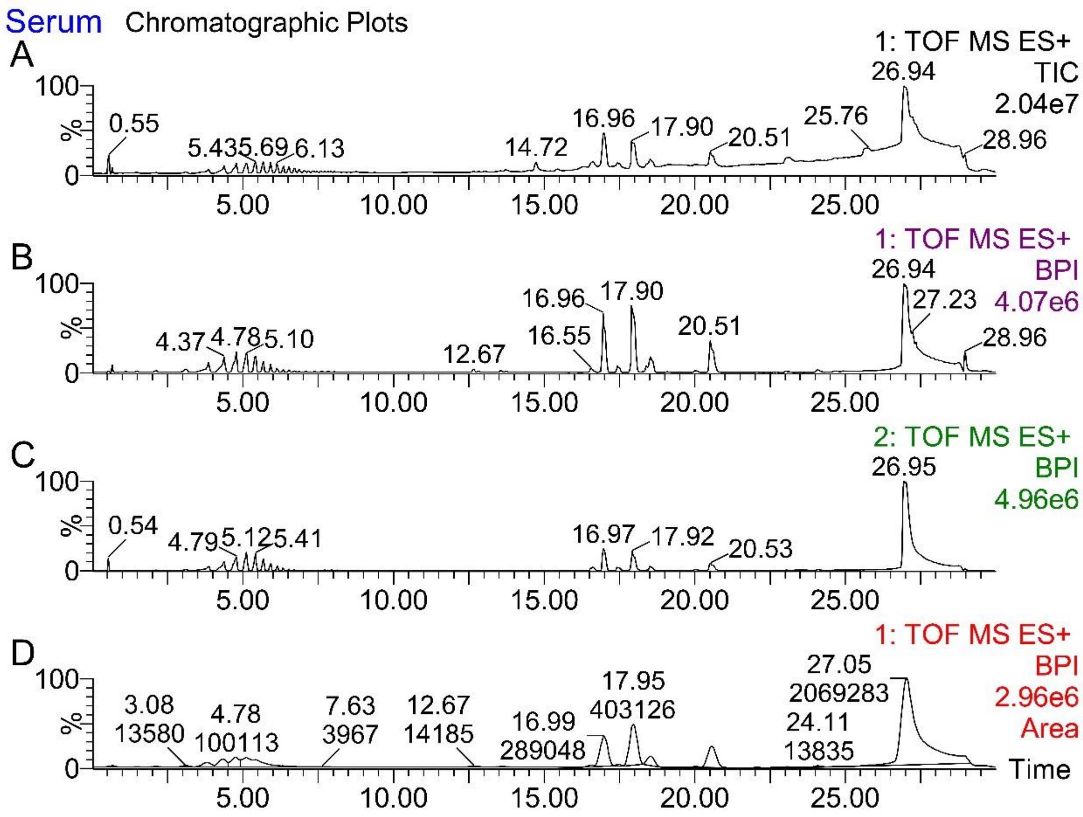

Figure S19. Total Ion Chromatogram (TIC) and Base Peak Ion (BPI) of Serum Samples.

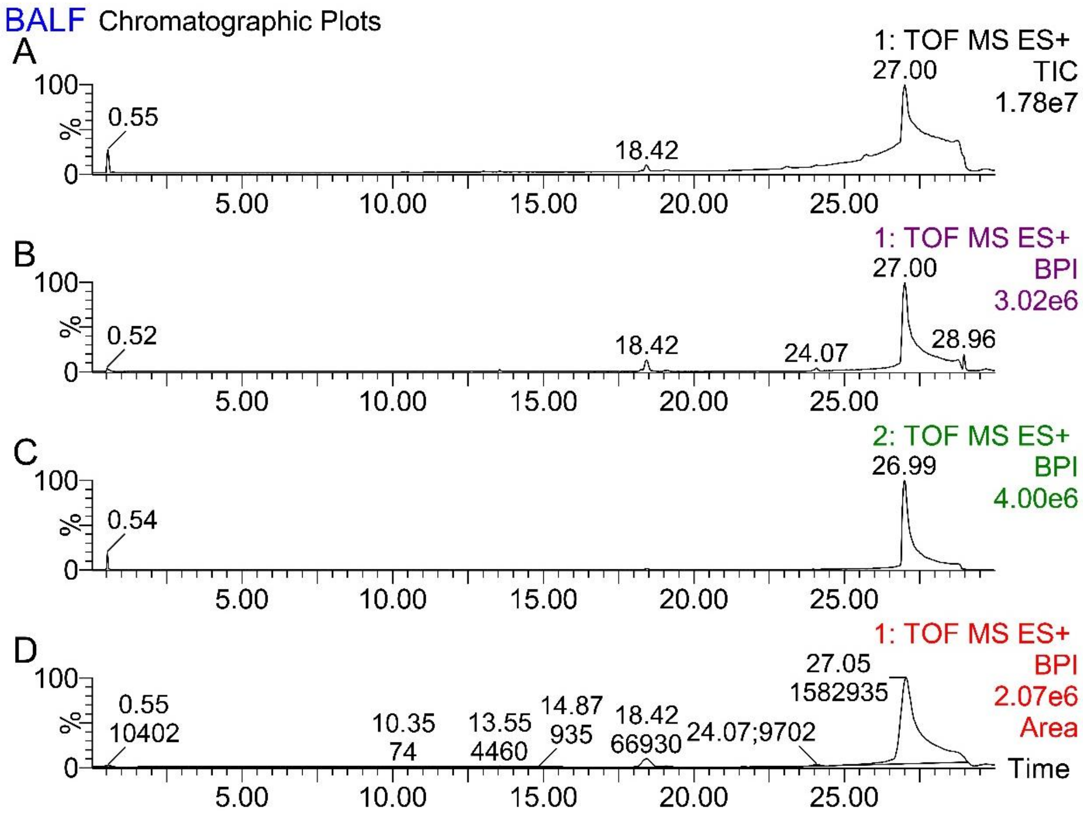

Figure S20. Total Ion Chromatogram (TIC) and Base Peak Ion (BPI) of BALF Samples.

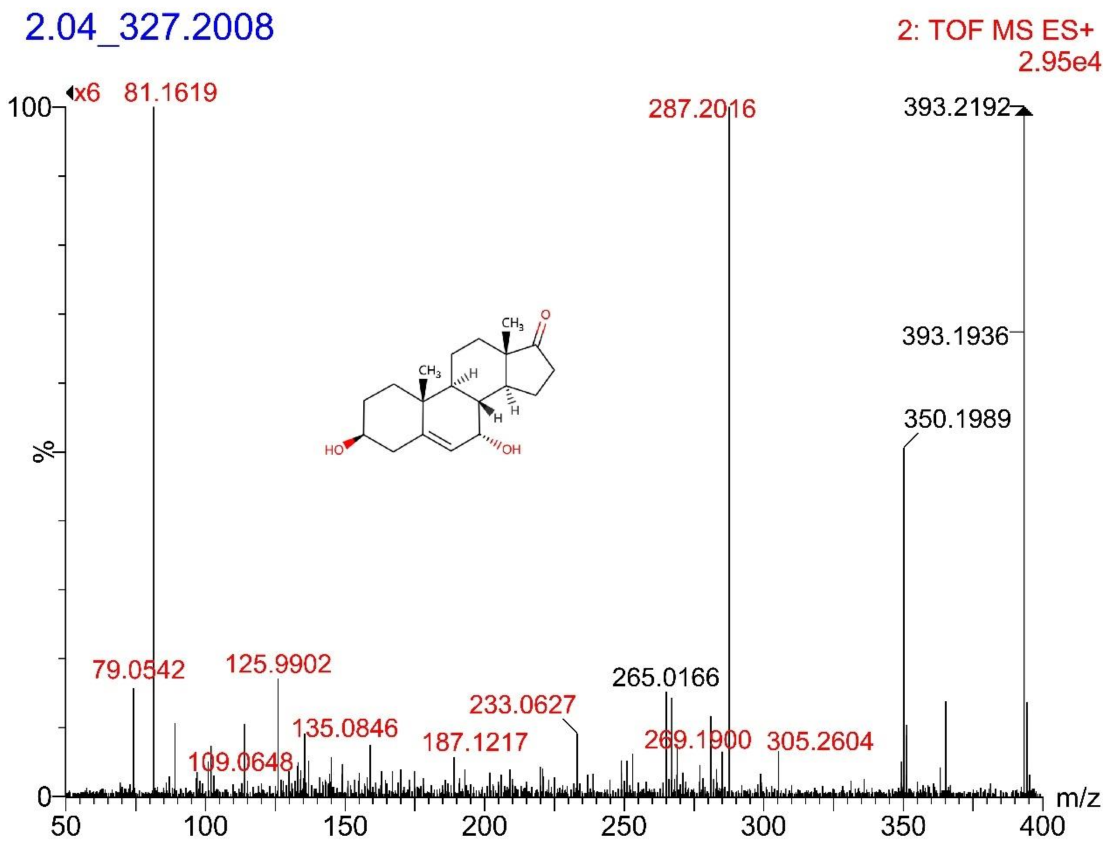

Figure S21. Identification Plot of Metabolite M1 with Mass Spectrum.

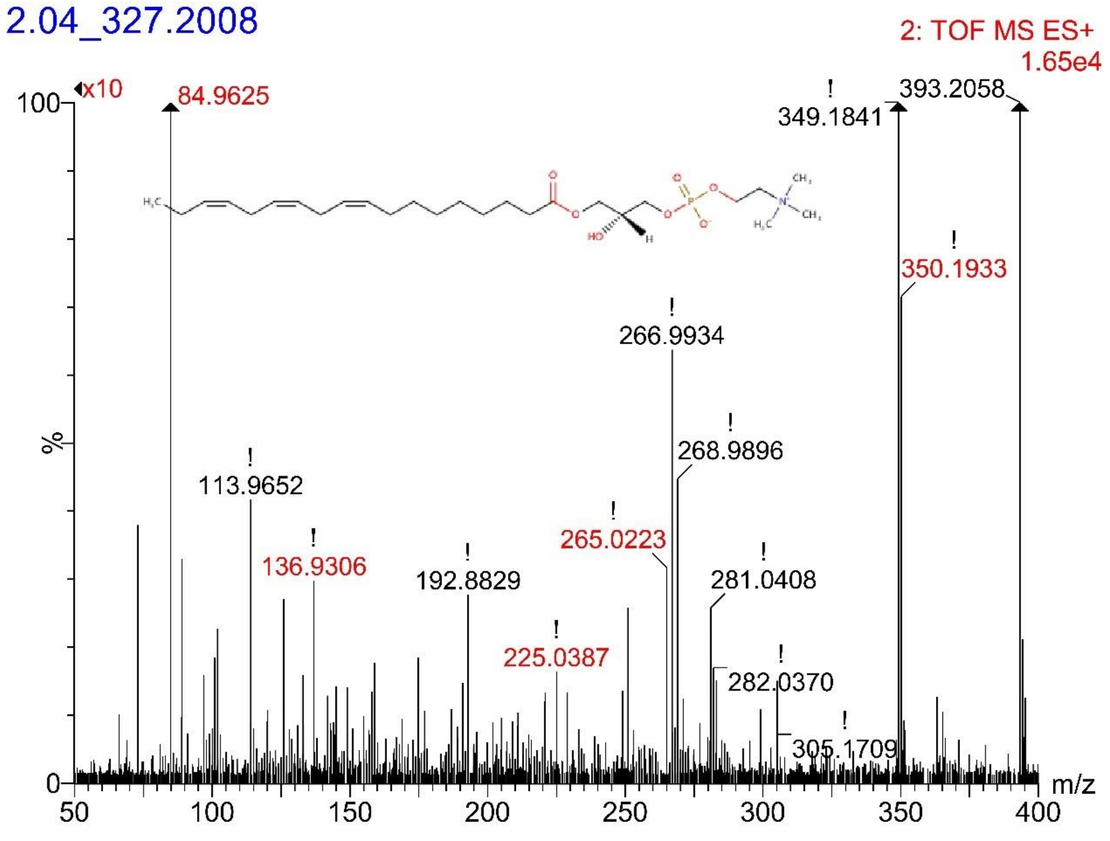

Figure S22. Identification Plot of Metabolite M2 with Mass Spectrum.

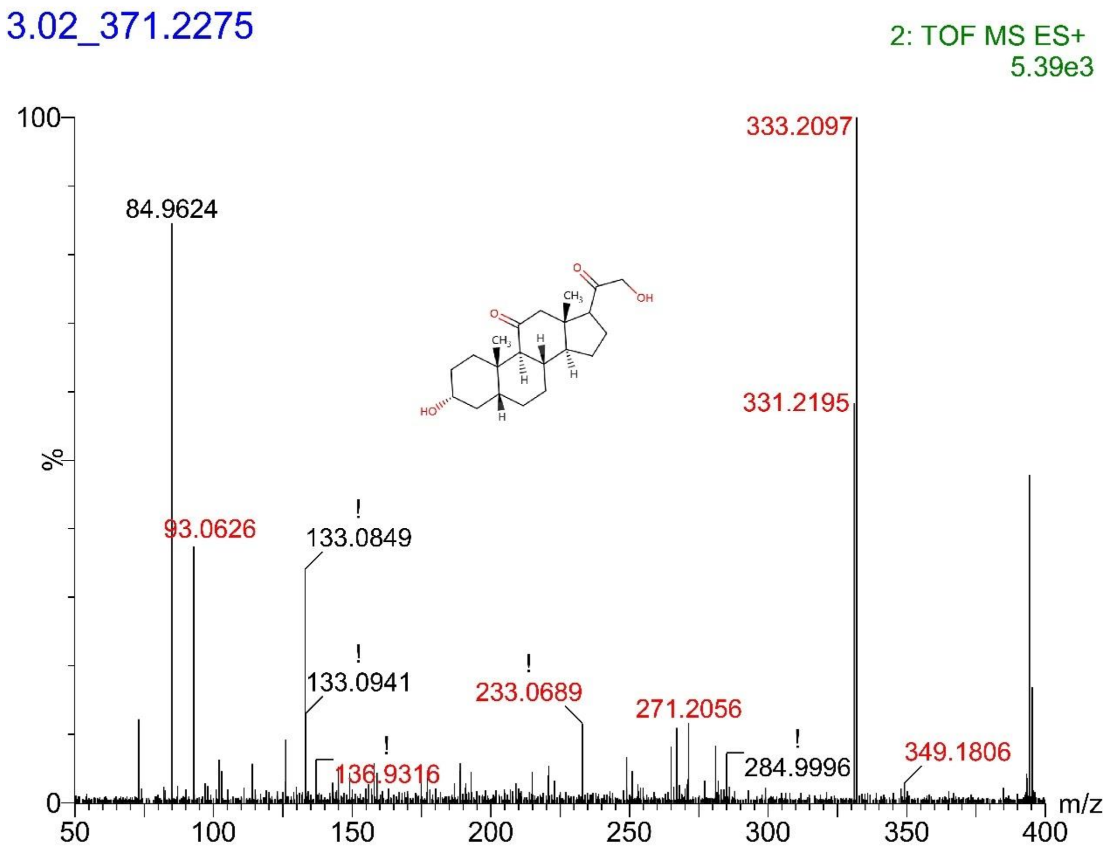

Figure S23. Identification Plot of Metabolite M3 with Mass Spectrum.

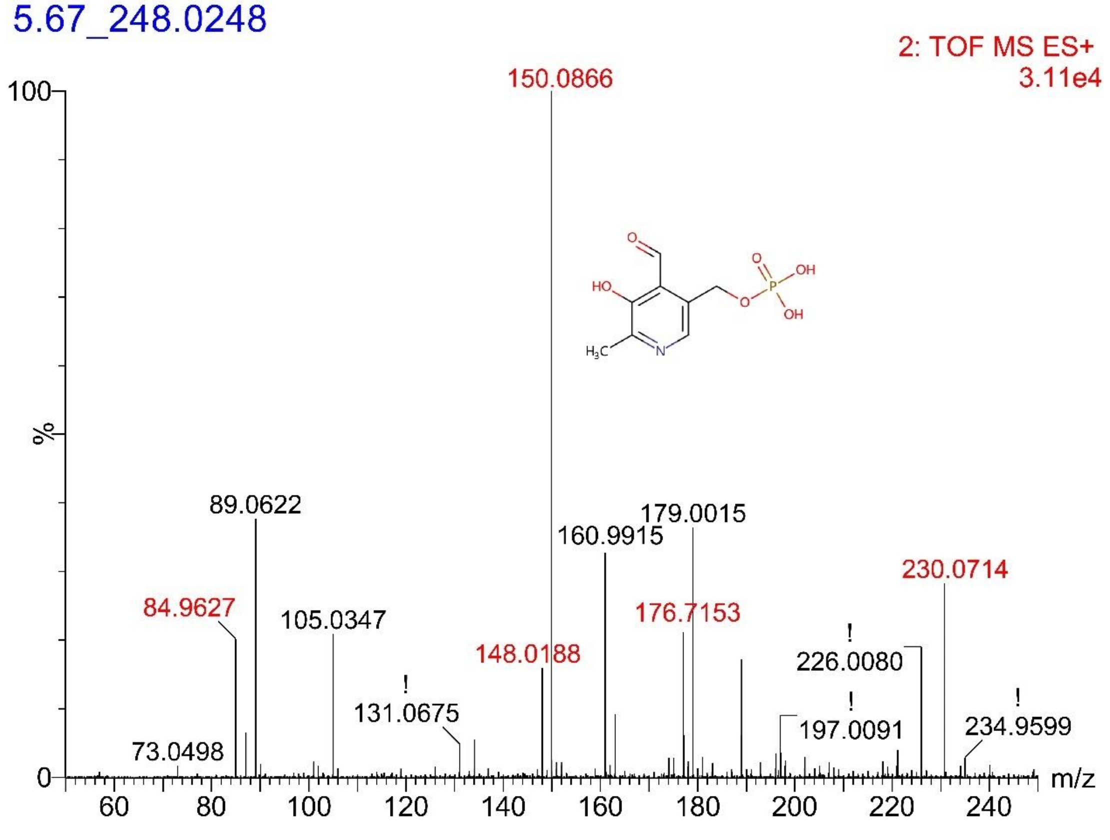

Figure S24. Identification Plot of Metabolite M4 with Mass Spectrum.

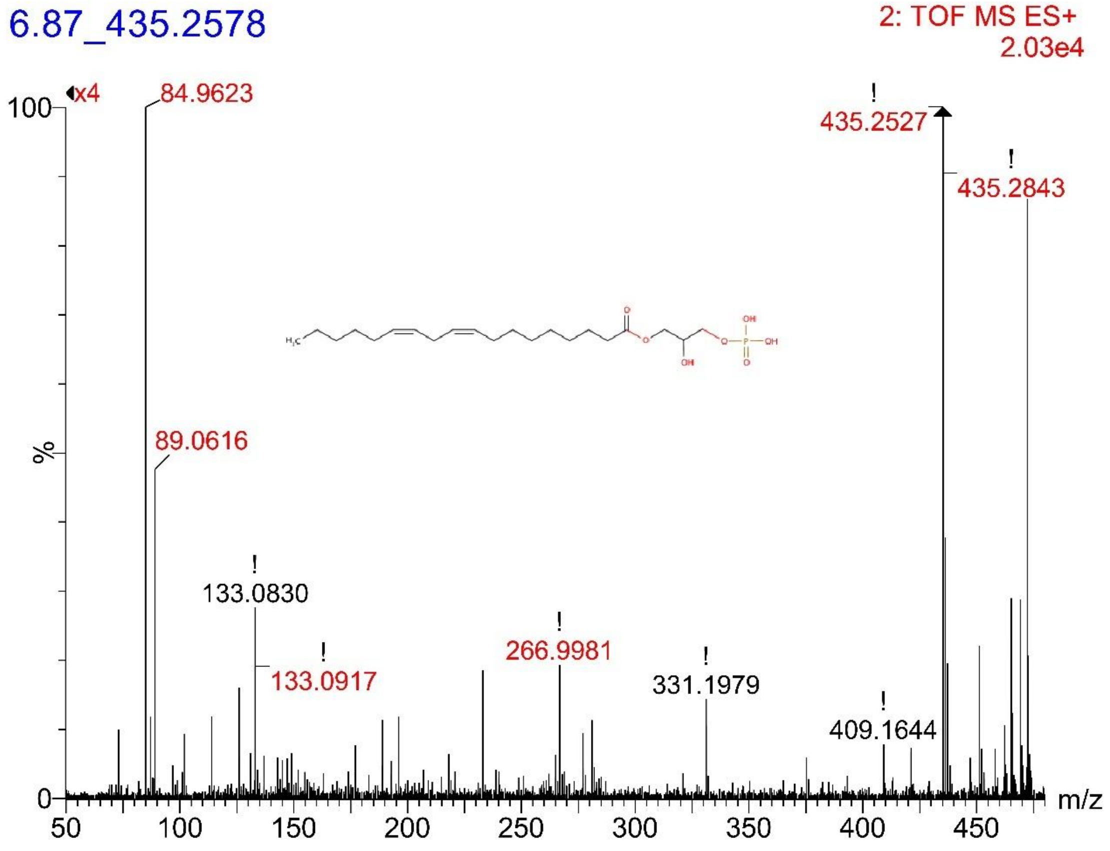

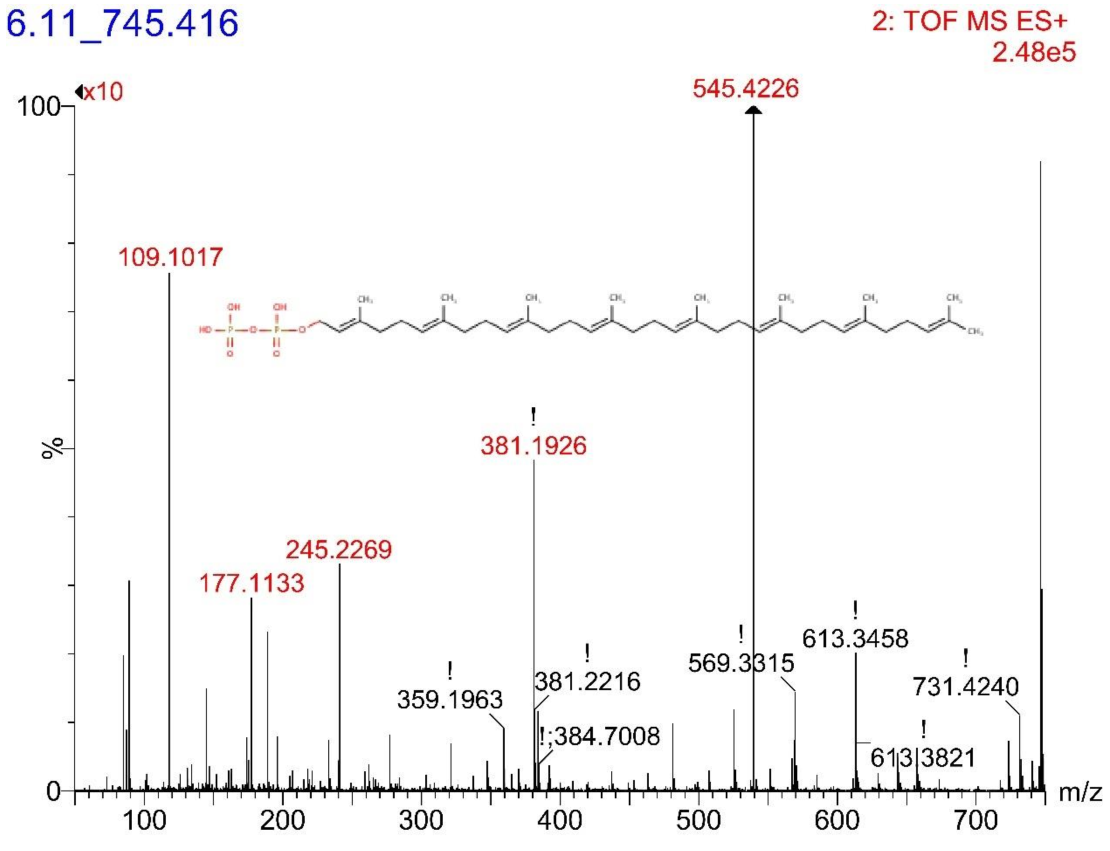

Figure S25. Identification Plot of Metabolite M5 with Mass Spectrum.

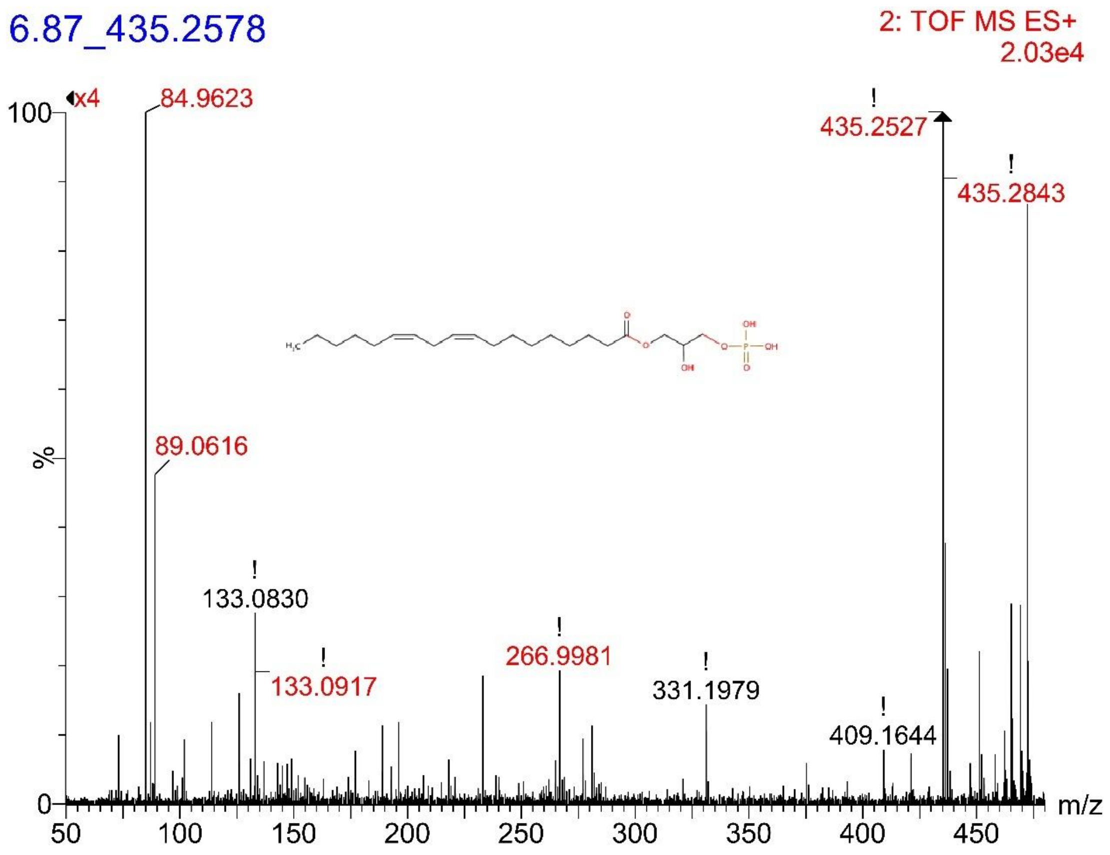

Figure S26. Identification Plot of Metabolite M6 with Mass Spectrum.

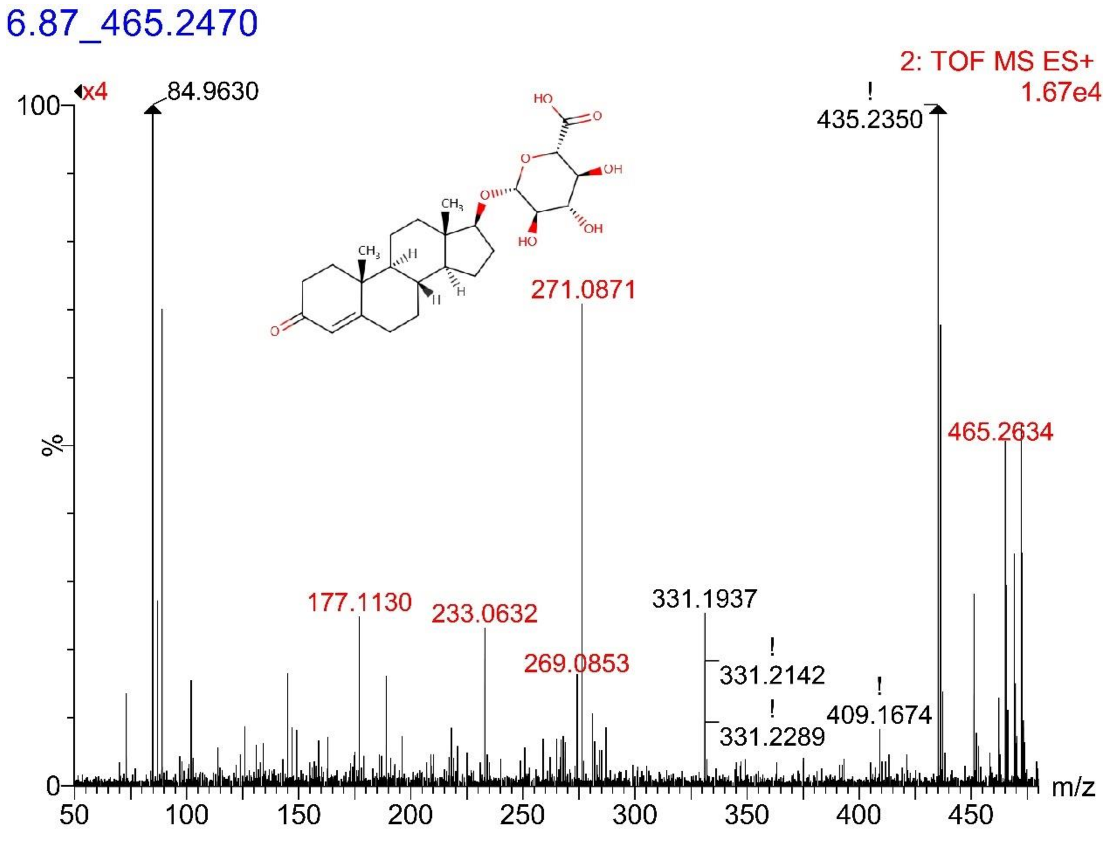

Figure S27. Identification Plot of Metabolite M7 with Mass Spectrum.

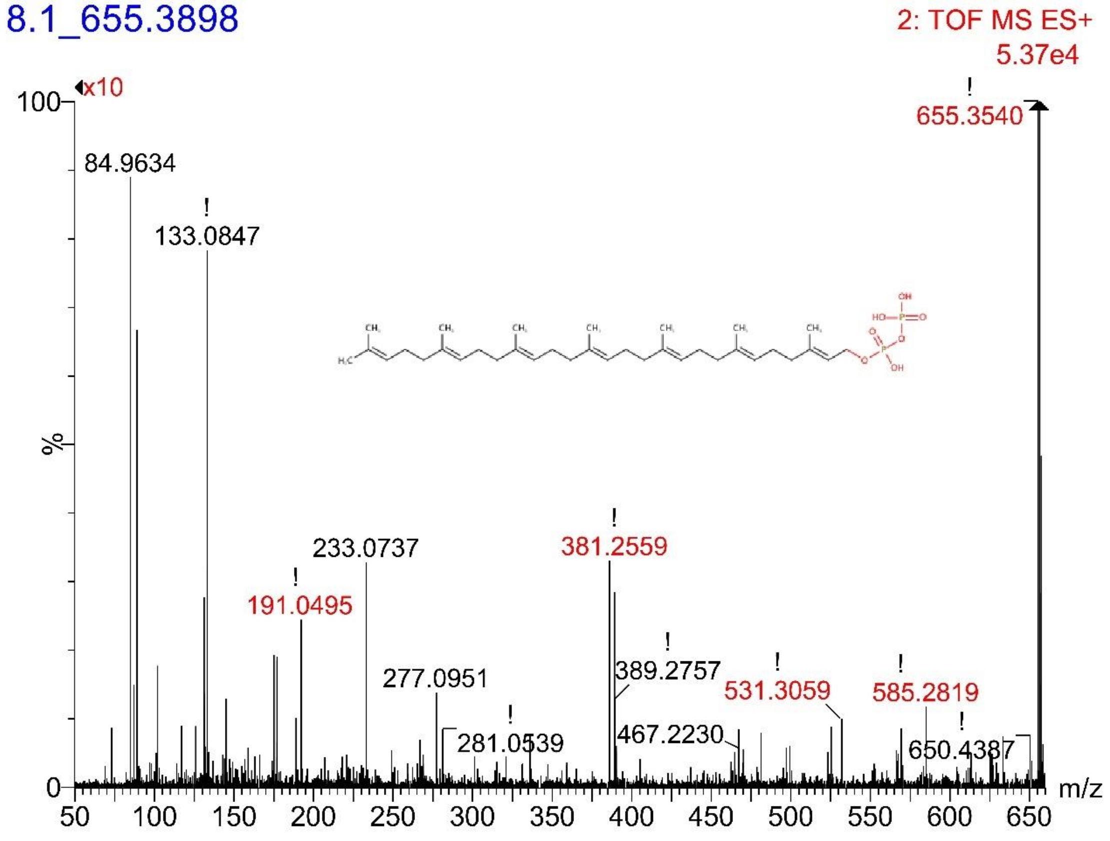

Figure S28. Identification Plot of Metabolite M8 with Mass Spectrum.

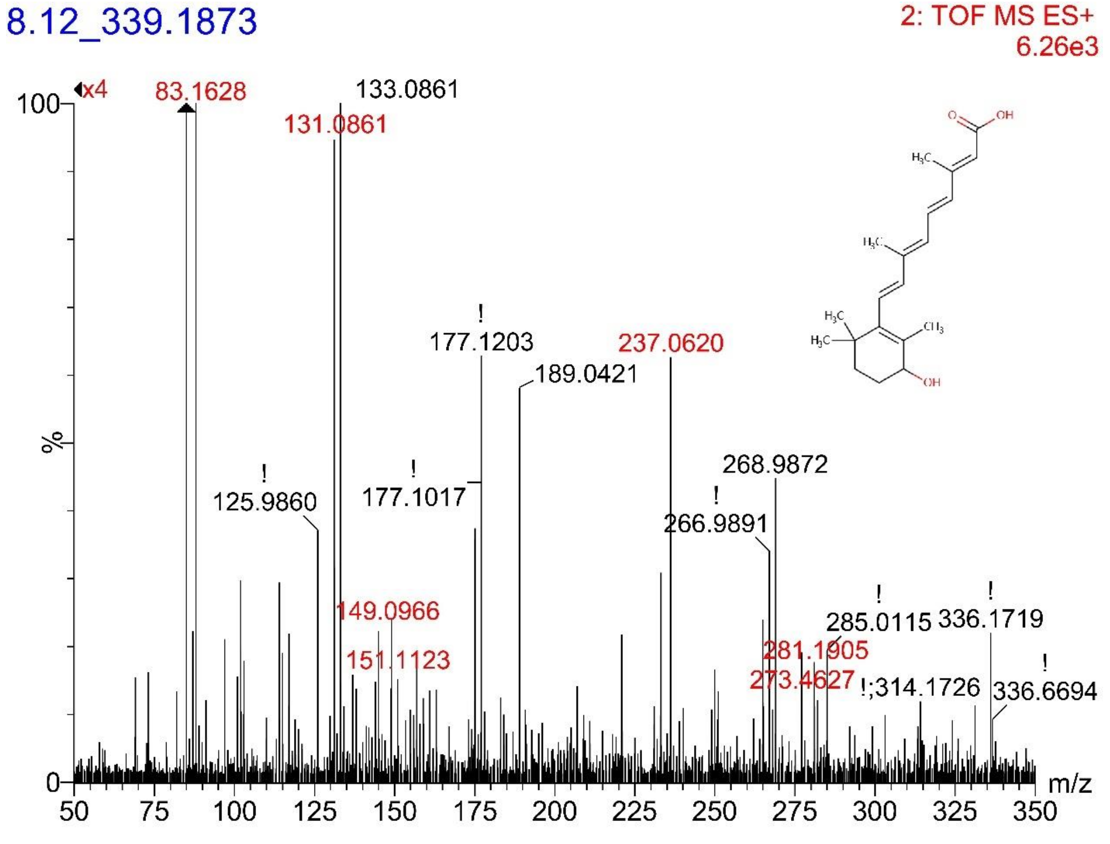

Figure S29. Identification Plot of Metabolite M9 with Mass Spectrum.

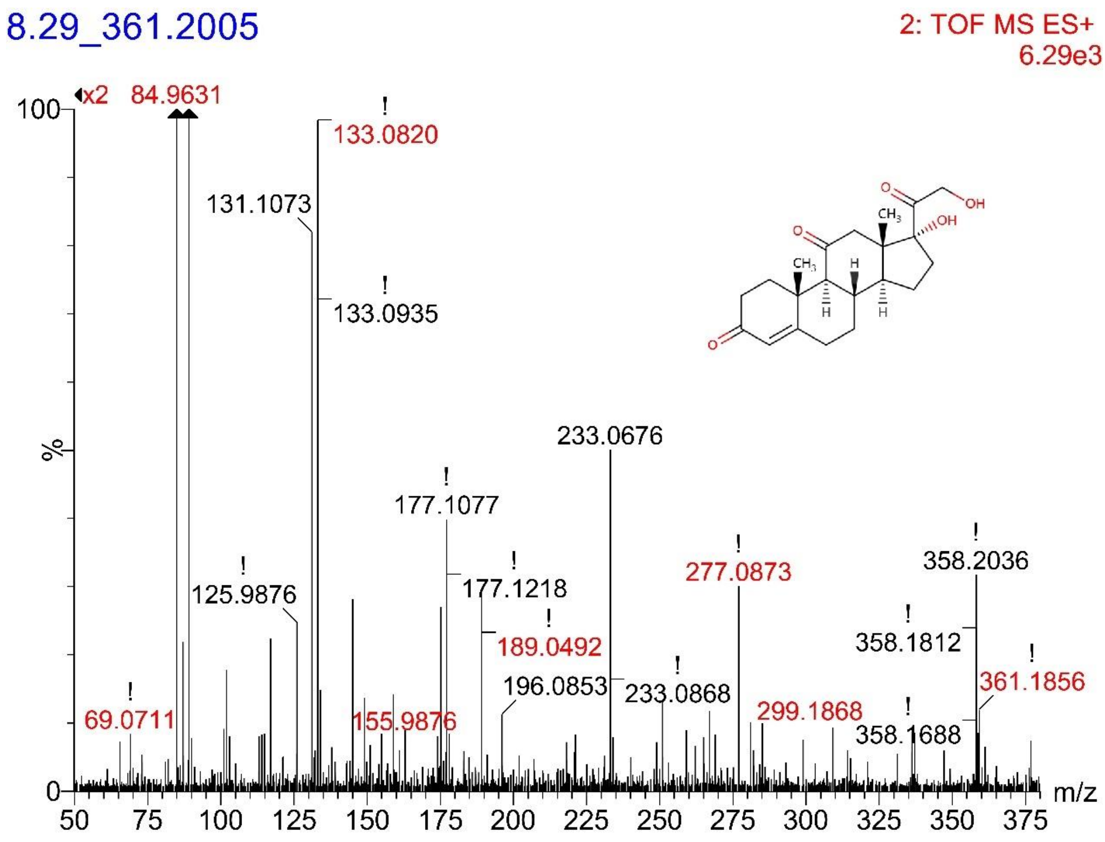

Figure S30. Identification Plot of Metabolite M10 with Mass Spectrum.

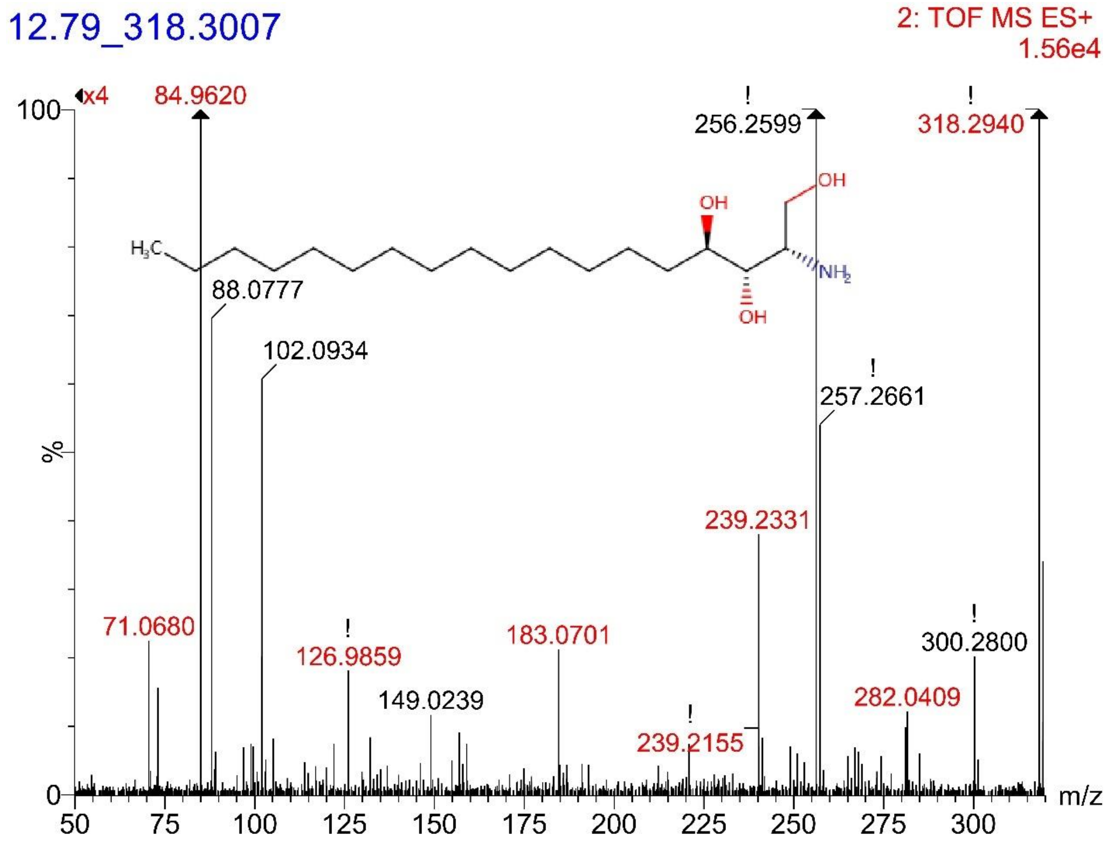

Figure S31. Identification Plot of Metabolite M11 with Mass Spectrum.

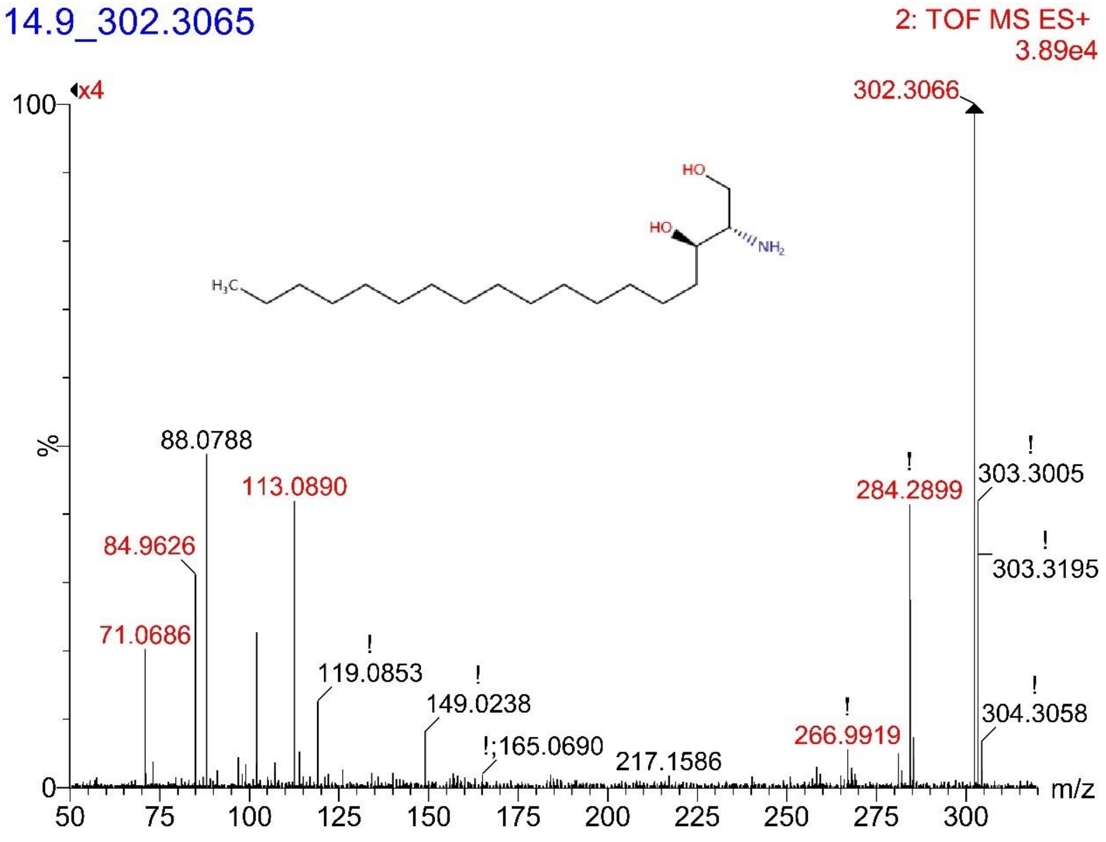

Figure S32. Identification Plot of Metabolite M12 with Mass Spectrum.

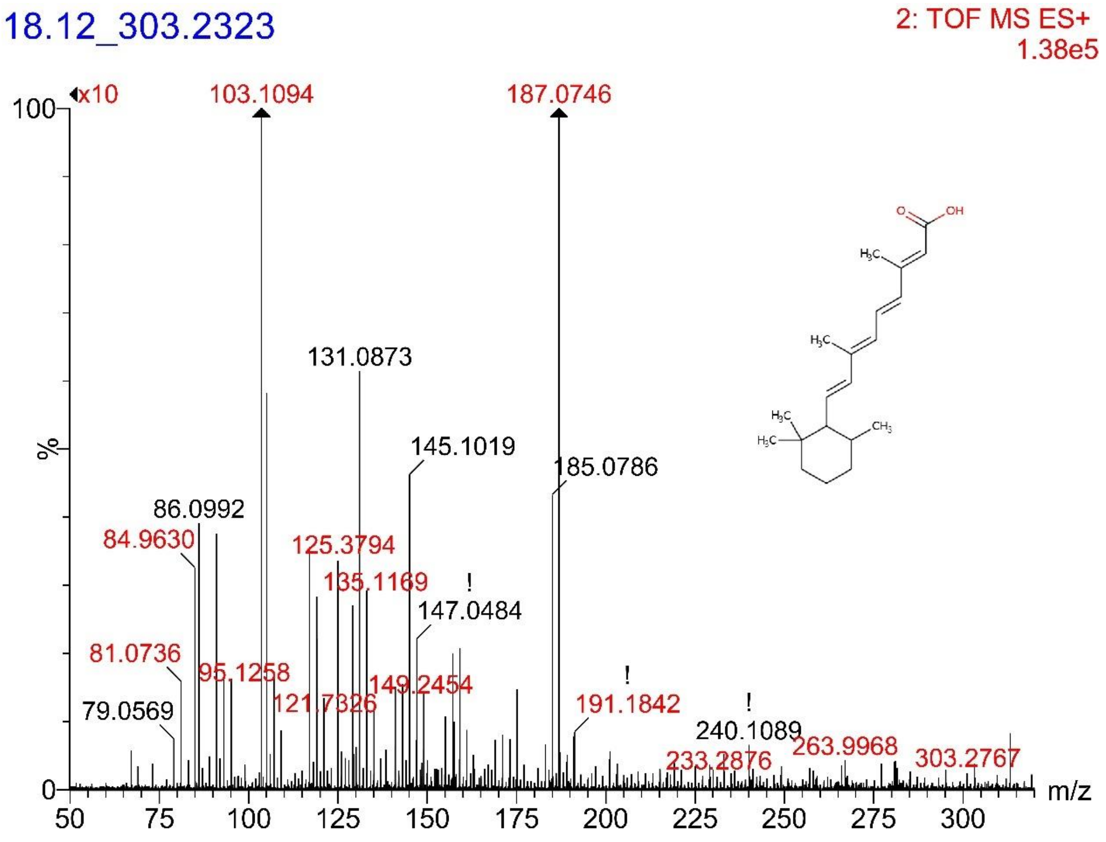

Figure S33. Identification Plot of Metabolite M13 with Mass Spectrum.

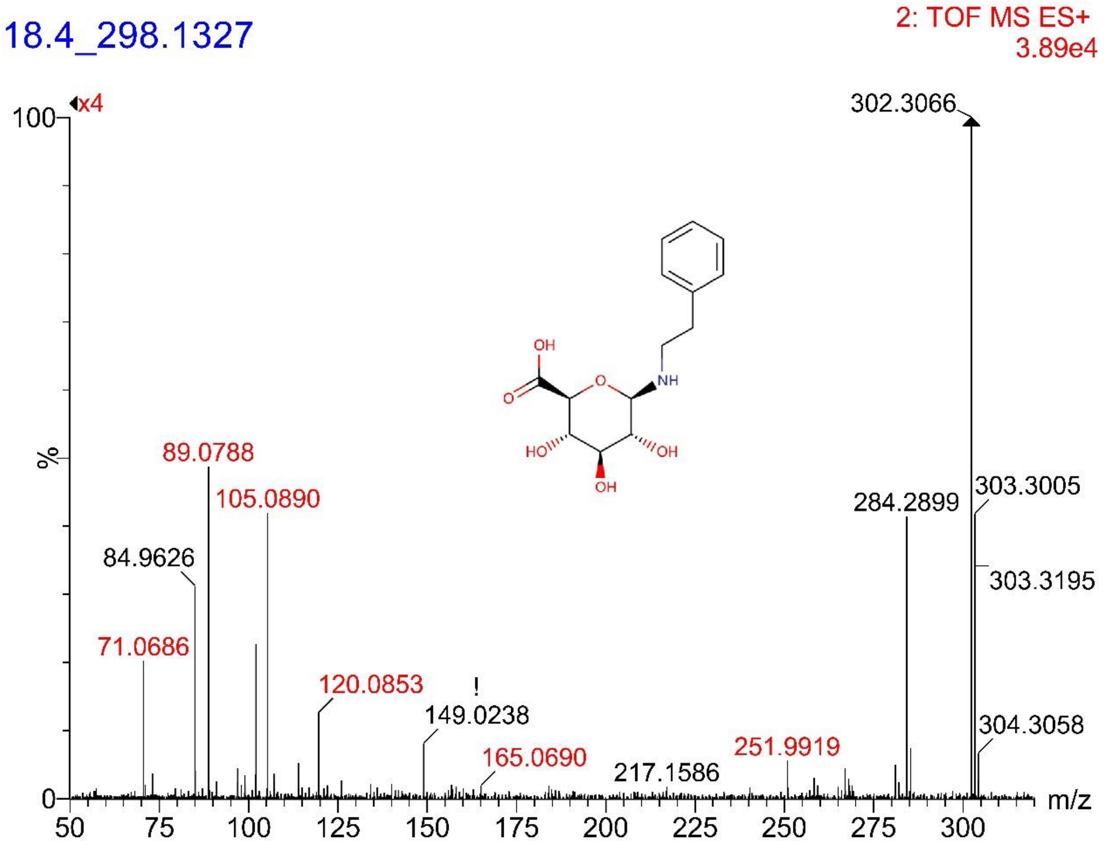

Figure S34. Identification Plot of Metabolite M14 with Mass Spectrum.

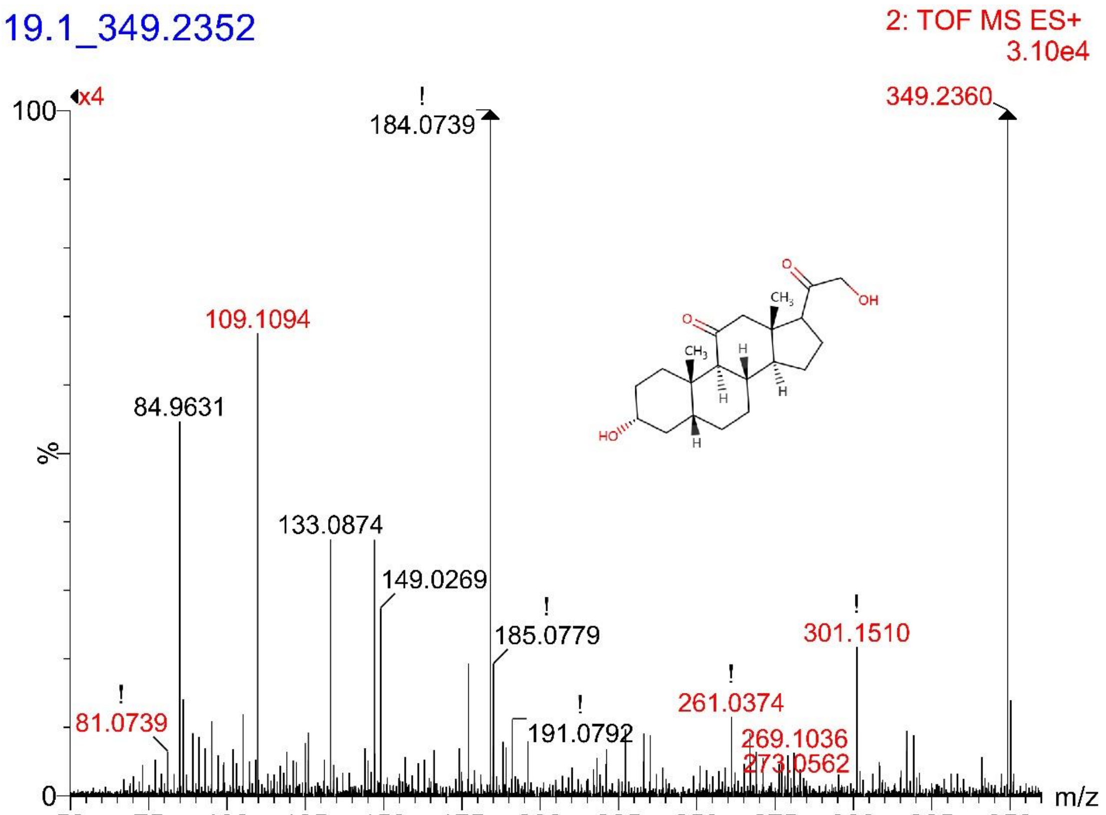

Figure S35. Identification Plot of Metabolite M15 with Mass Spectrum.

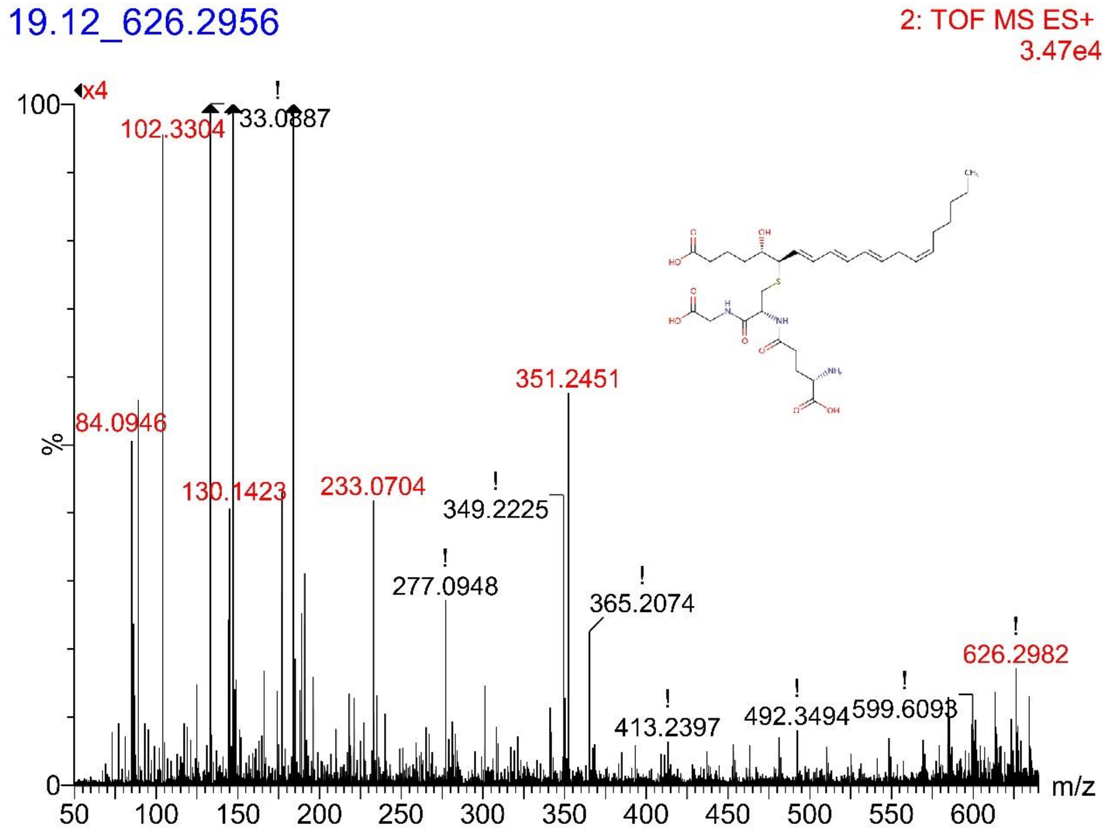

Figure S36. Identification Plot of Metabolite M16 with Mass Spectrum.

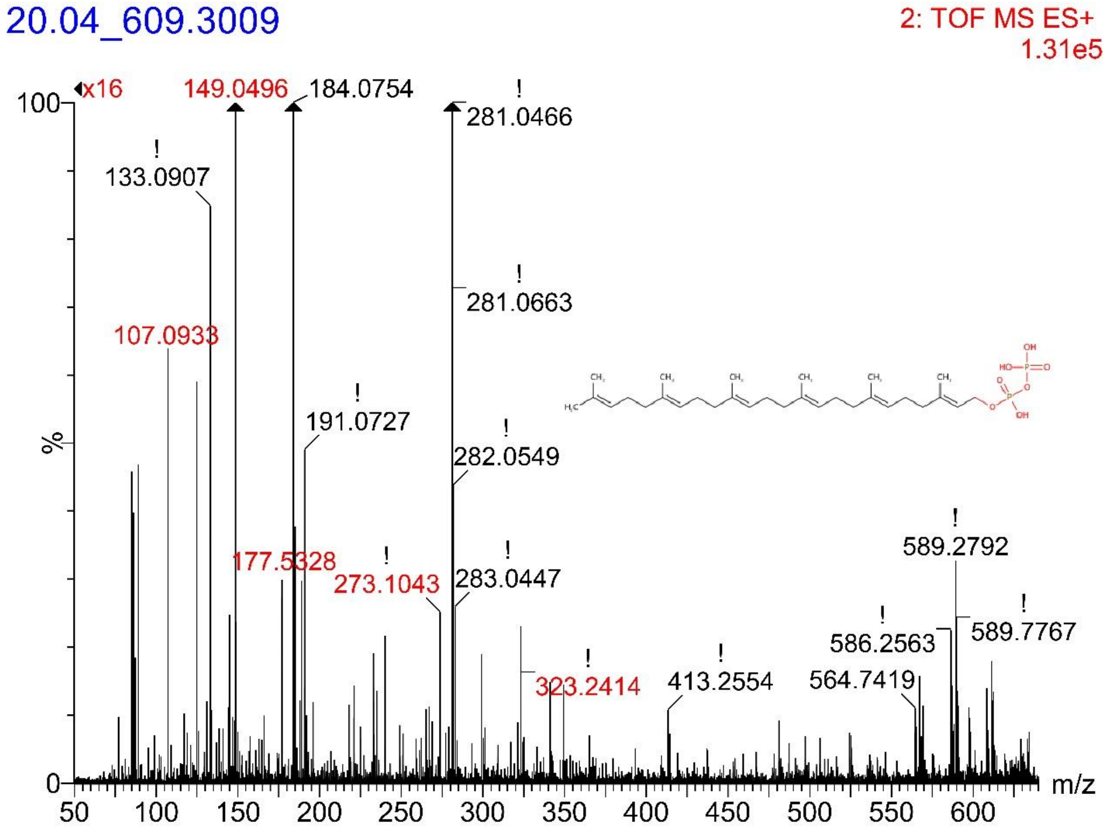

Figure S37. Identification Plot of Metabolite M17 with Mass Spectrum.

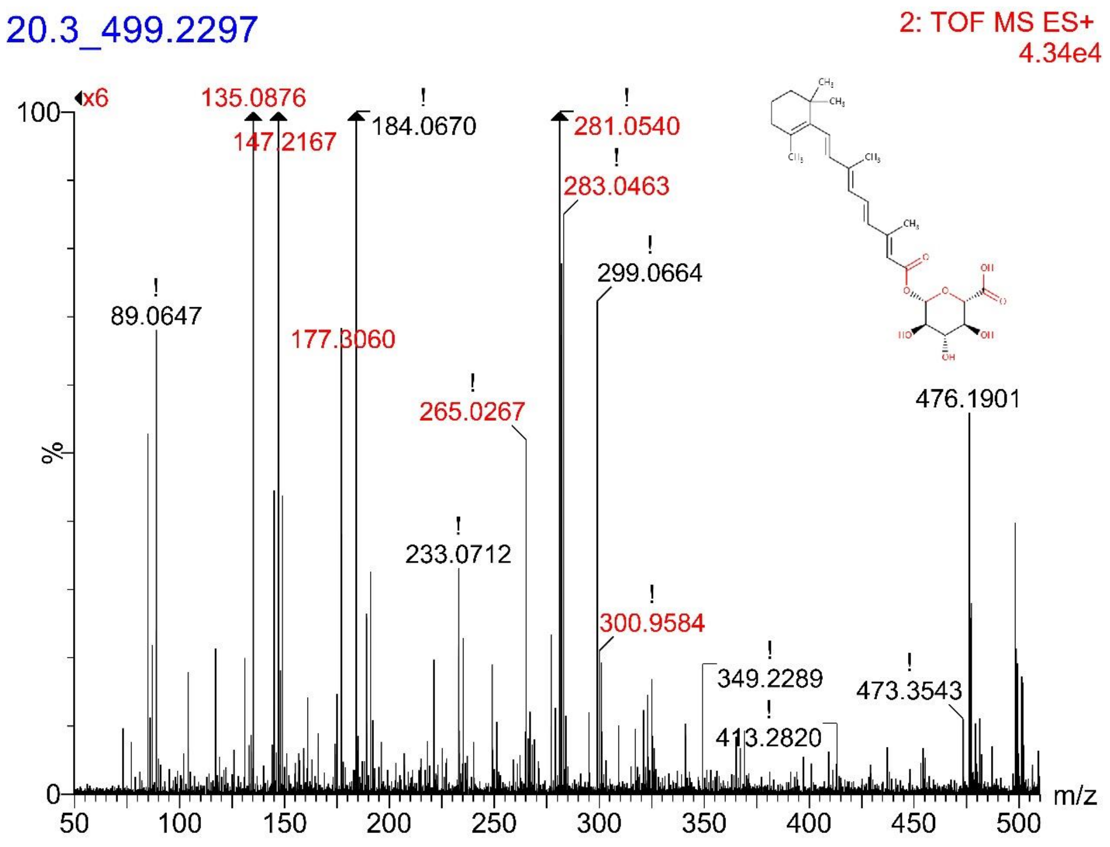

Figure S38. Identification Plot of Metabolite M18 with Mass Spectrum.

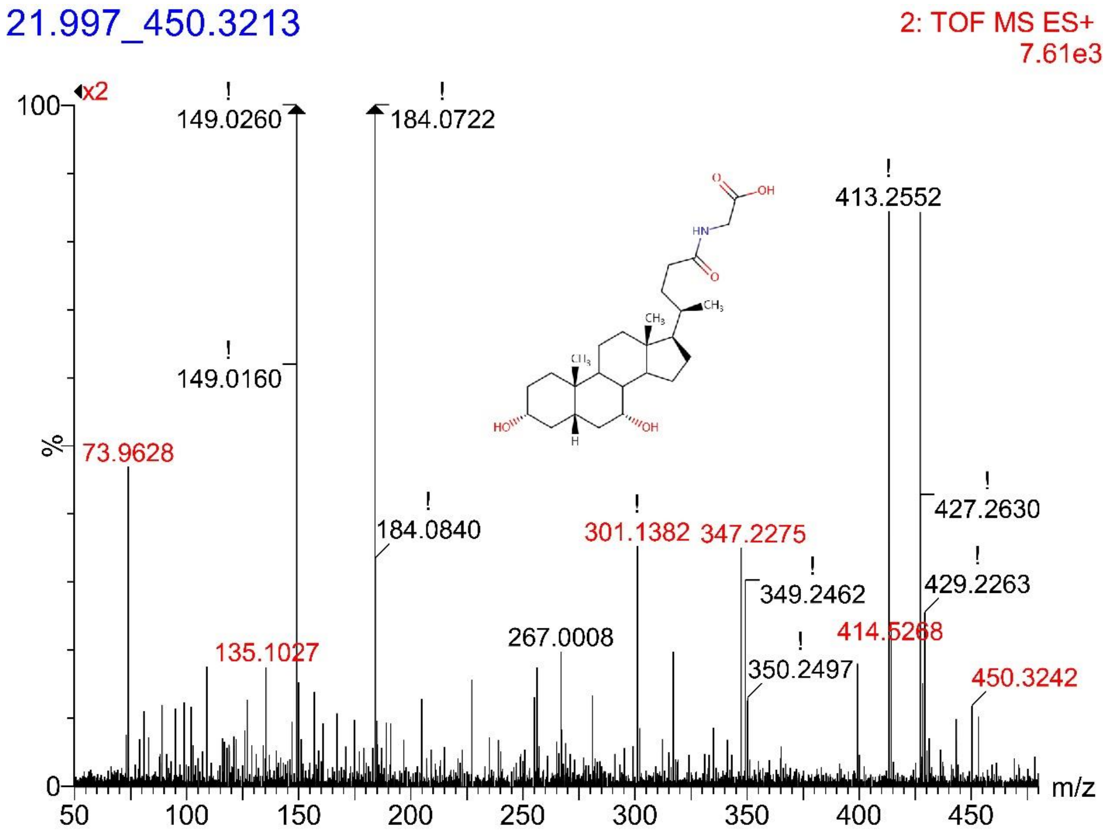

Figure S39. Identification Plot of Metabolite M19 with Mass Spectrum.

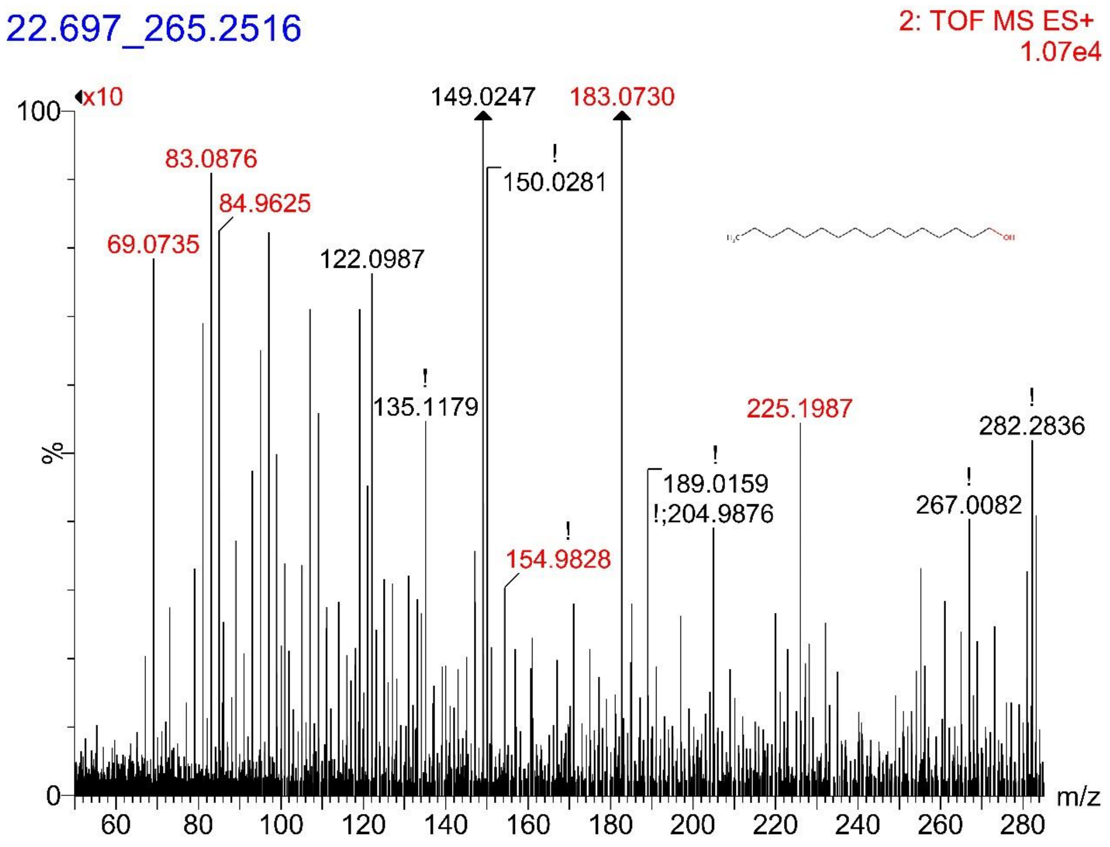

Figure S40. Identification Plot of Metabolite M20 with Mass Spectrum.

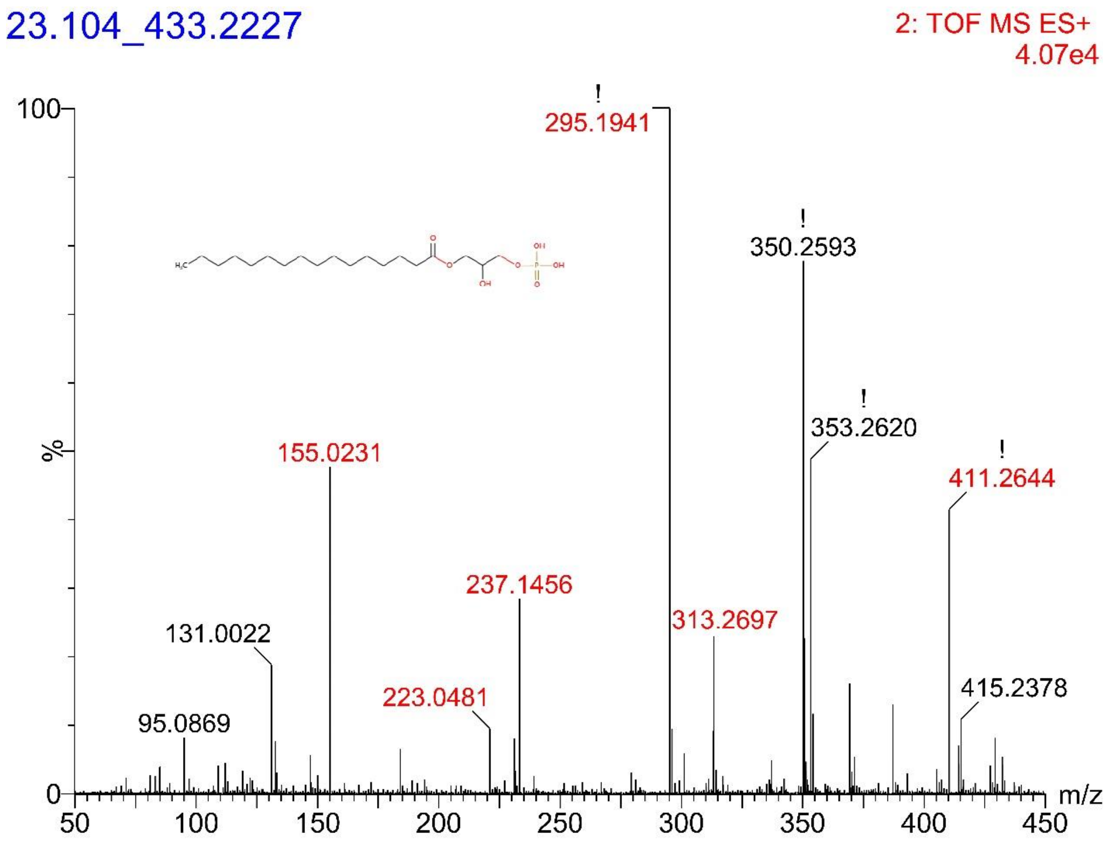

Figure S41. Identification Plot of Metabolite M21 with Mass Spectrum.

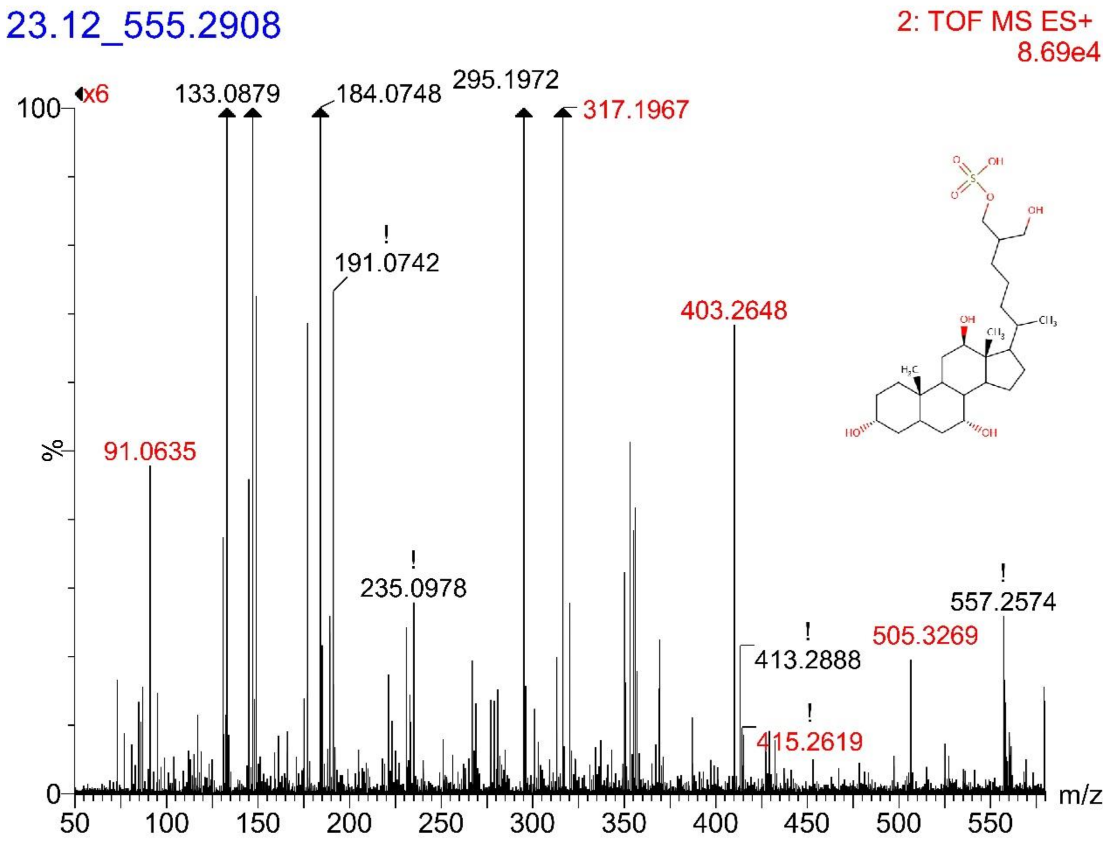

Figure S42. Identification Plot of Metabolite M22 with Mass Spectrum.

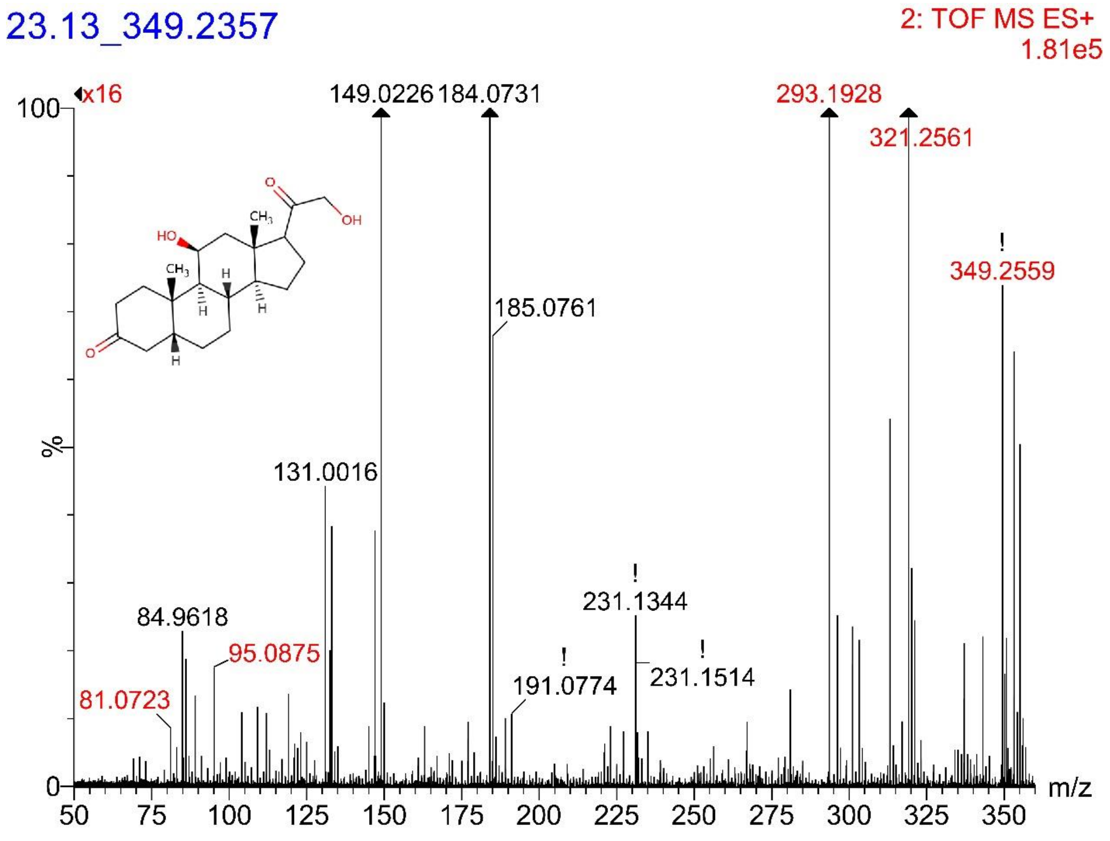

Figure S43. Identification Plot of Metabolite M23 with Mass Spectrum.

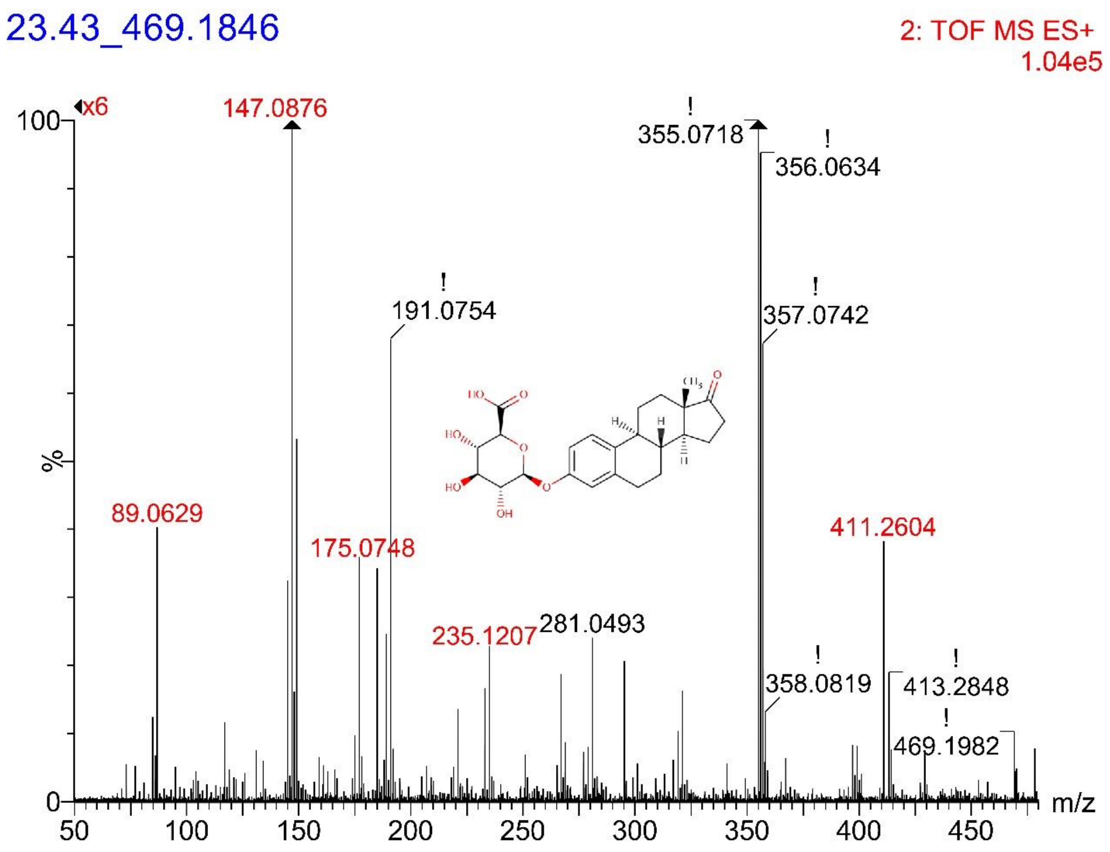

Figure S44. Identification Plot of Metabolite M24 with Mass Spectrum.

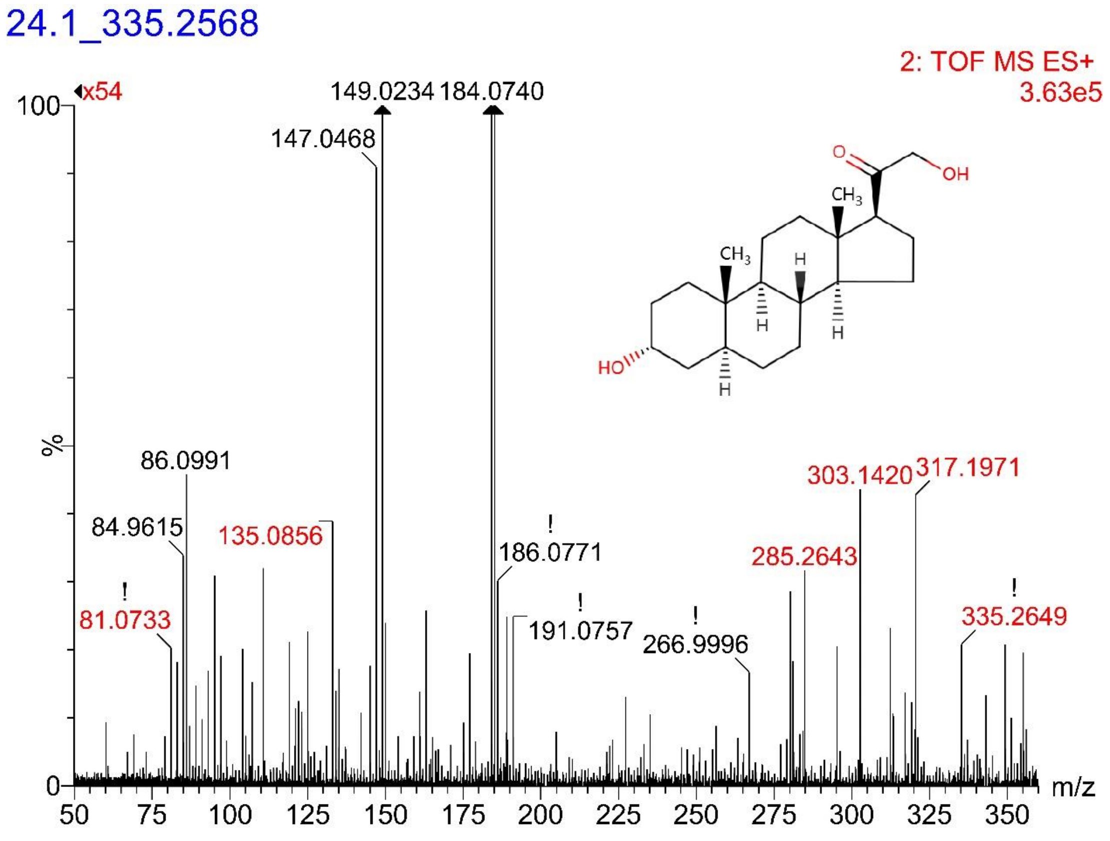

Figure S45. Identification Plot of Metabolite M25 with Mass Spectrum.

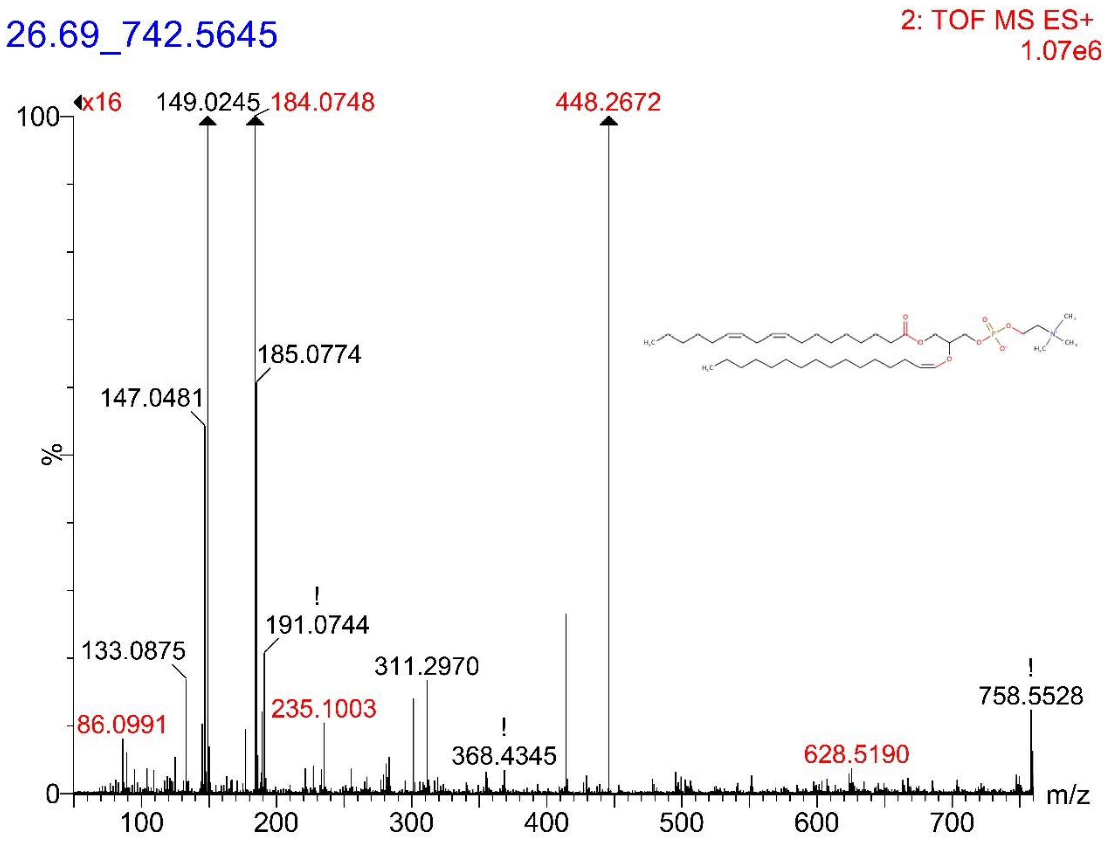

Figure S46. Identification Plot of Metabolite M26 with Mass Spectrum.

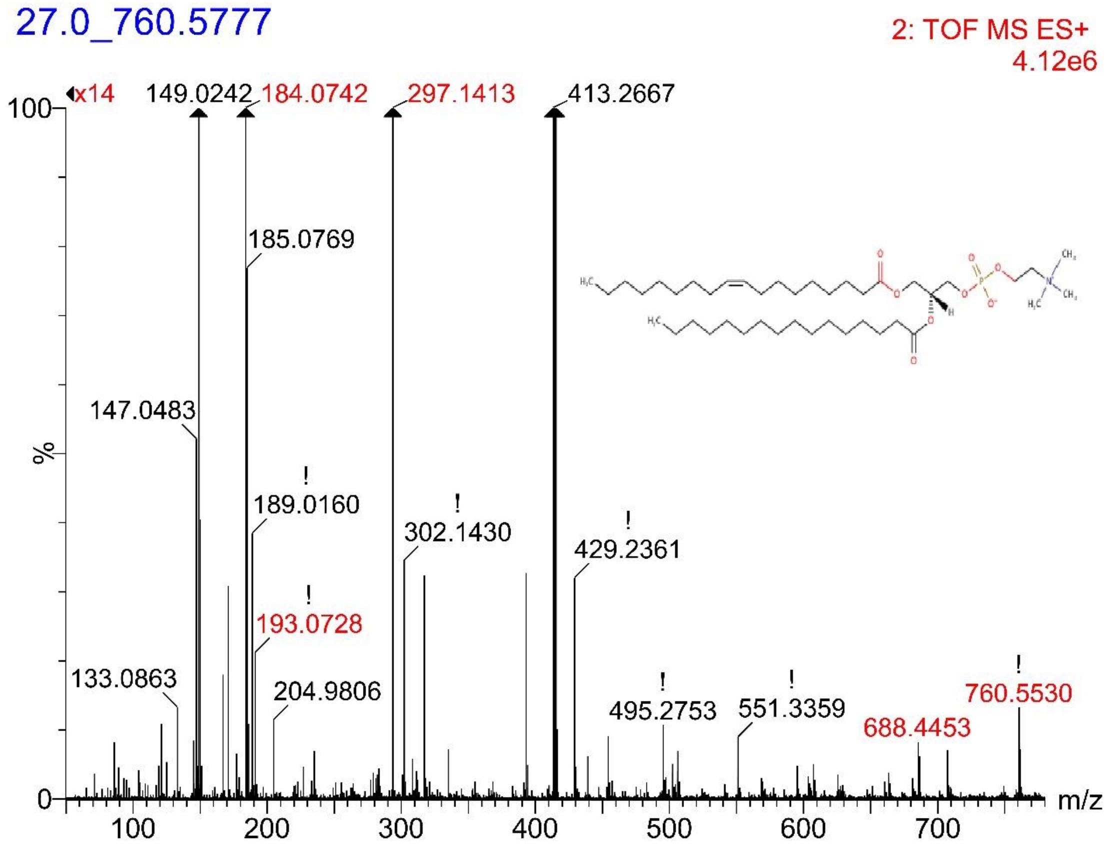

Figure S47. Identification Plot of Metabolite M27 with Mass Spectrum.

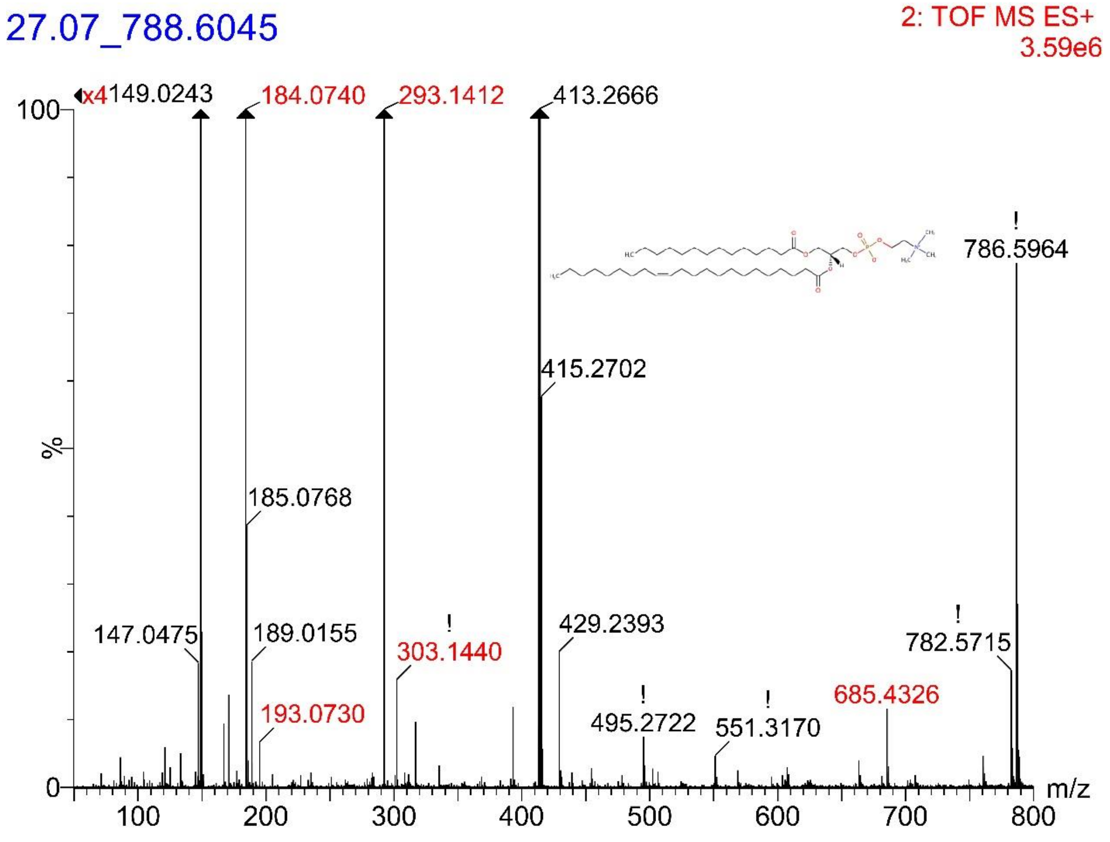

Figure S48. Identification Plot of Metabolite M28 with Mass Spectrum.

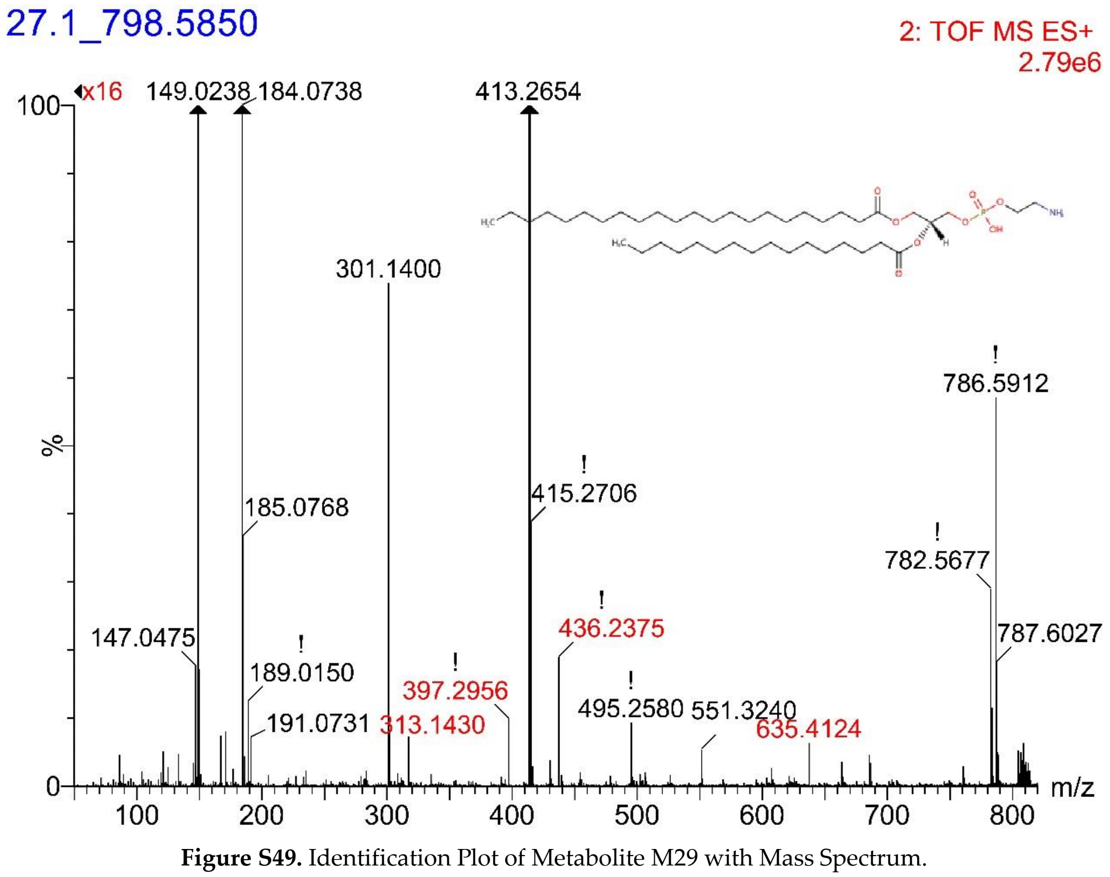

Figure S49. Identification Plot of Metabolite M29 with Mass Spectrum.

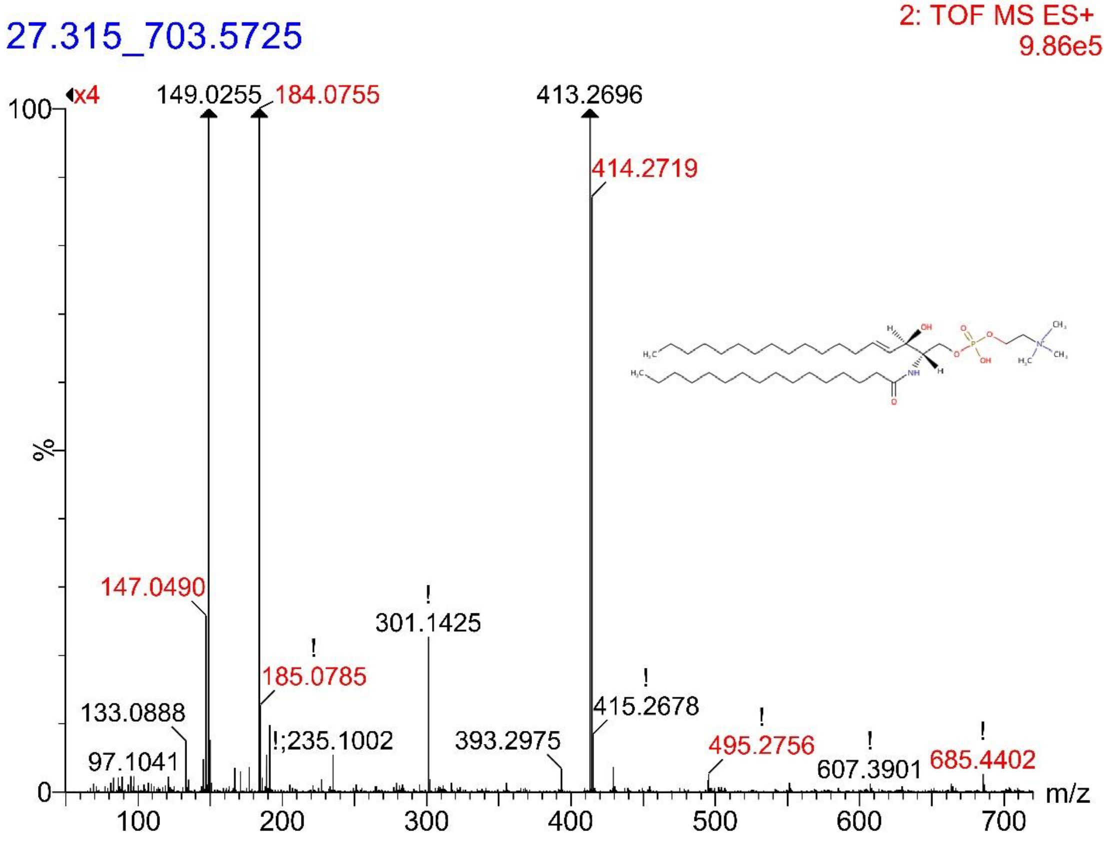

Figure S50. Identification Plot of Metabolite M30 with Mass Spectrum.

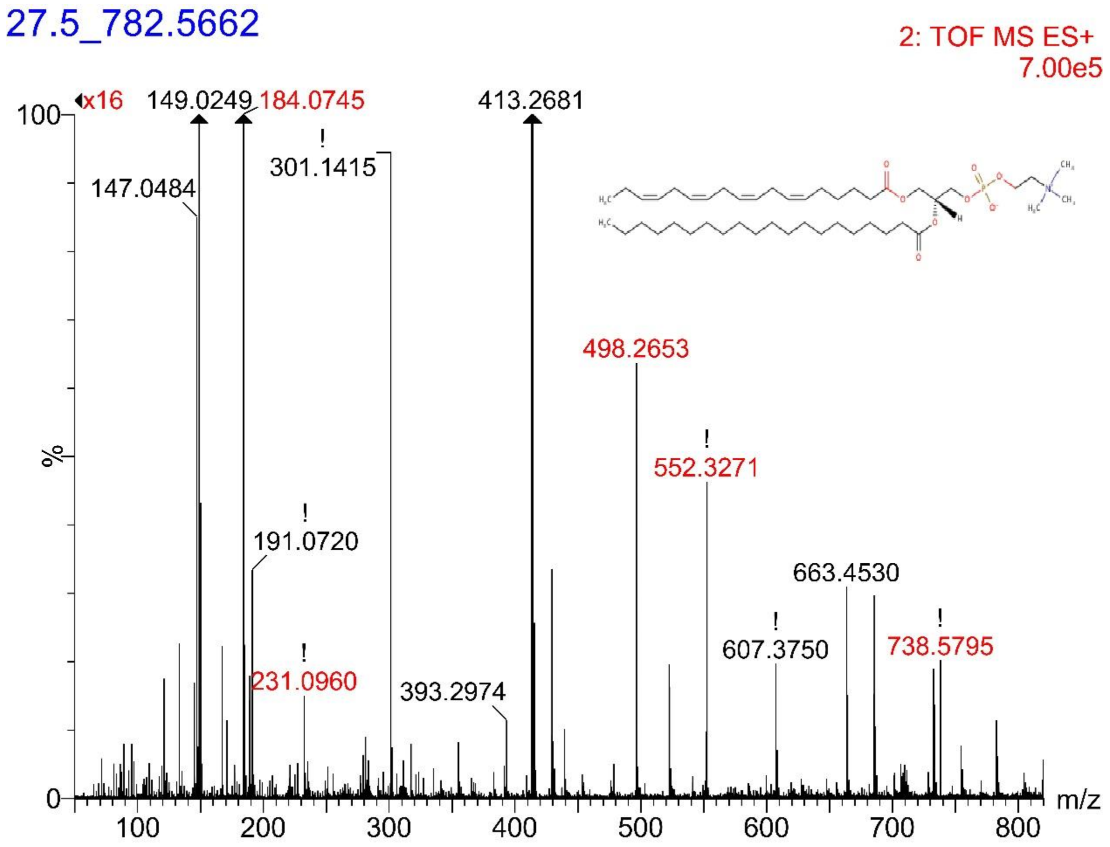

Figure S51. Identification Plot of Metabolite M31 with Mass Spectrum.

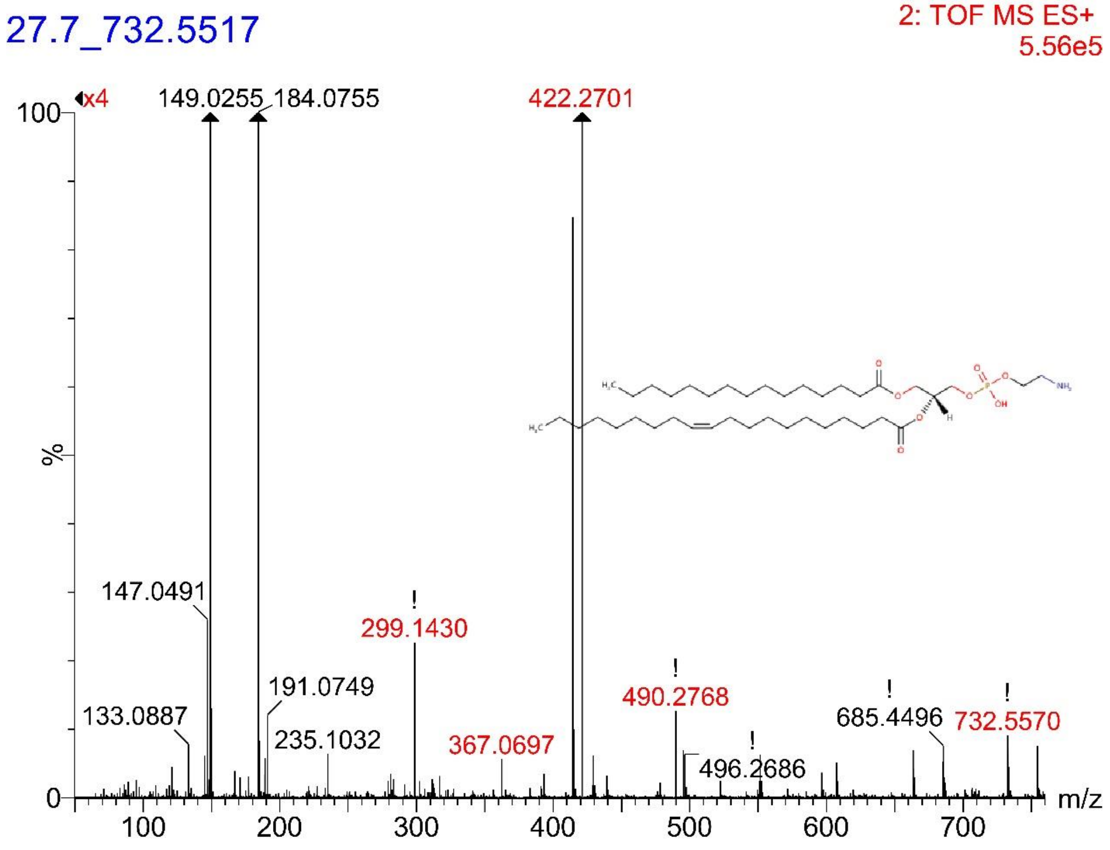

Figure S52. Identification Plot of Metabolite M32 with Mass Spectrum.

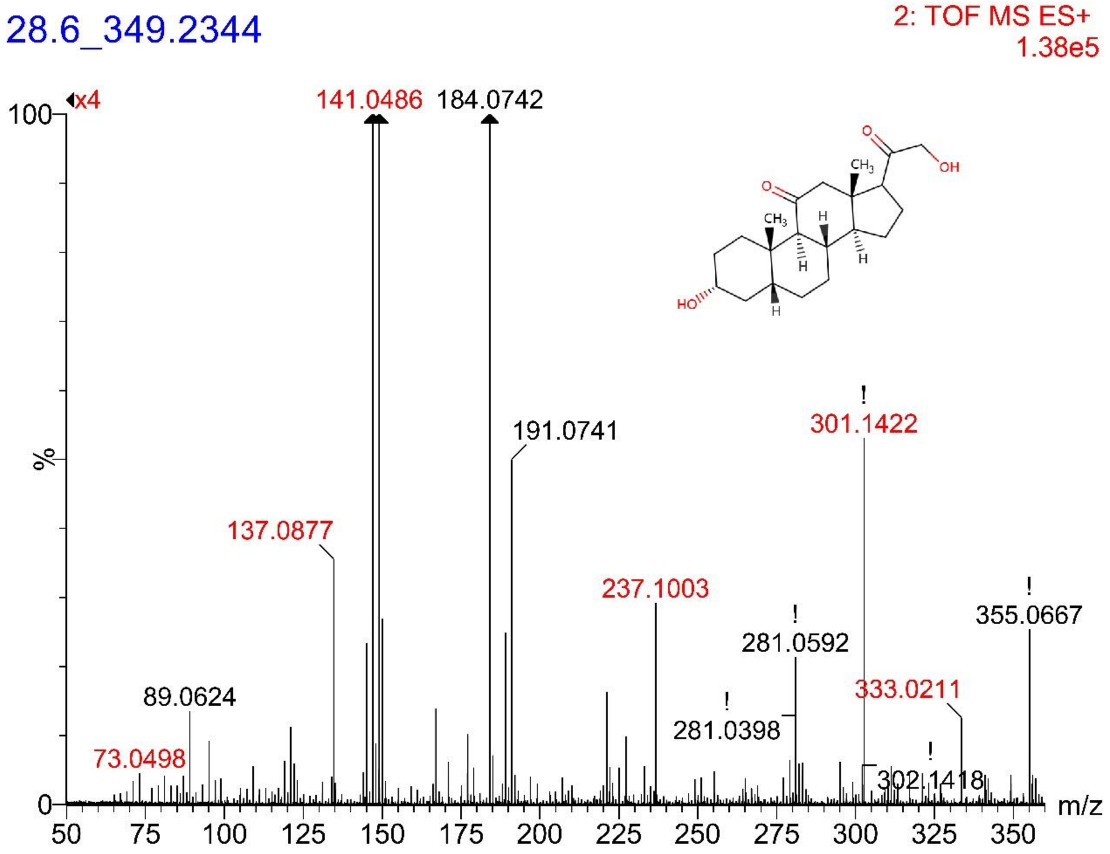

Figure S53. Identification Plot of Metabolite M33 with Mass Spectrum.

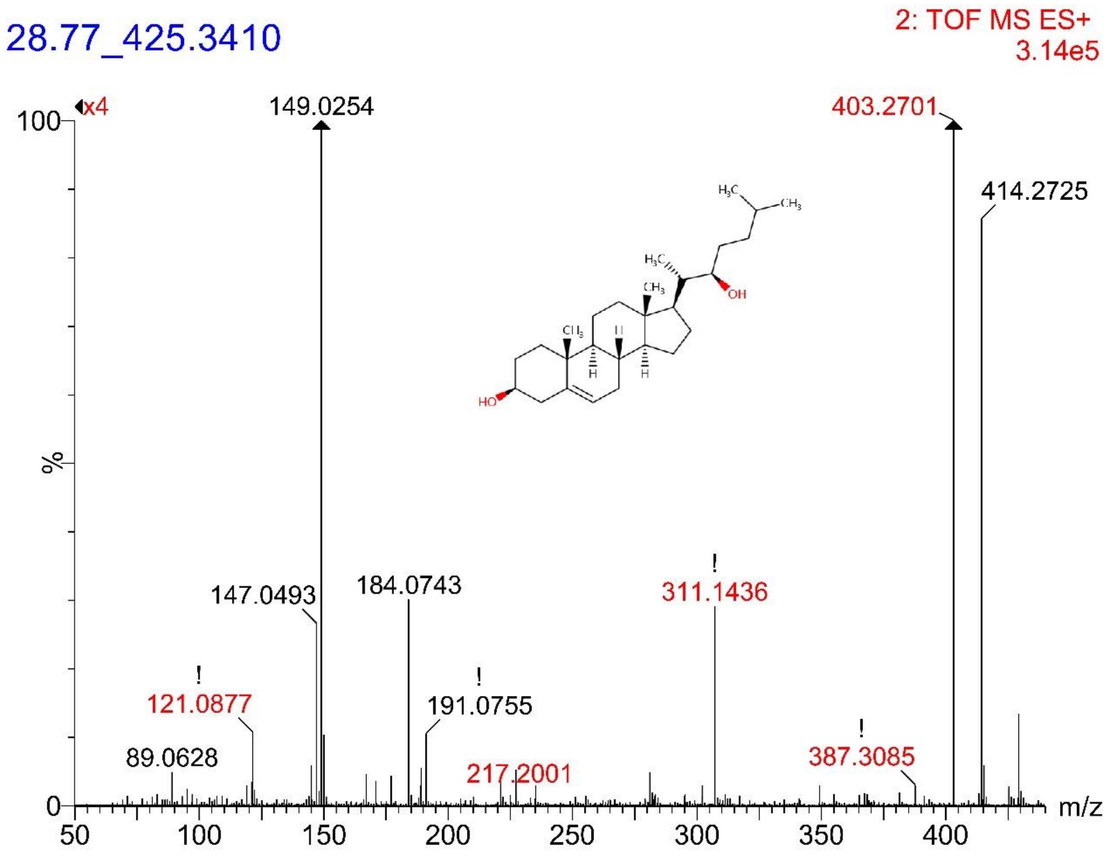

Figure S54. Identification Plot of Metabolite M34 with Mass Spectrum.

Table S14. The Metabolic Prediction Result (in Part) of the Microbiota in NEA Based on PICRUST

KO ID	Description of Function	*p* value (NDR vs. NEA)
K07570	general stress protein 13	0.000193887
K16619	phospholipase C / alpha-toxin	0.000211194
K11635	putative ABC transport system ATP-binding protein	0.00053108
K05739	uncharacterized protein	0.000576456
K13275	major intracellular serine protease	0.000653228
K14439	SWI/SNF-related matrix-associated actin-dependent	0.000653402
K13815	two-component system, response regulator RpfG	0.000718875
K09681	LysR family transcriptional regulator, transcription	0.000781819
K06979	macrolide phosphotransferase	0.00079561
K08168	MFS transporter, metal-tetracycline-proton antiporter	0.000930036
K06344	spore coat protein Z	0.001022808
K17737	LysR family transcriptional regulator, carnitine	0.001077033
K08152	MFS transporter, DHA1 family, multidrug resistance	0.001154285
K06414	stage V sporulation protein M	0.001287591
K19005	lipoteichoic acid synthase	0.00153677
K01942	biotin---protein ligase	0.001600332
K00260	glutamate dehydrogenase	0.001657444
K12268	accessory secretory protein Asp1	0.001757039
K03489	GntR family transcriptional regulator, transcriptional	0.001762219

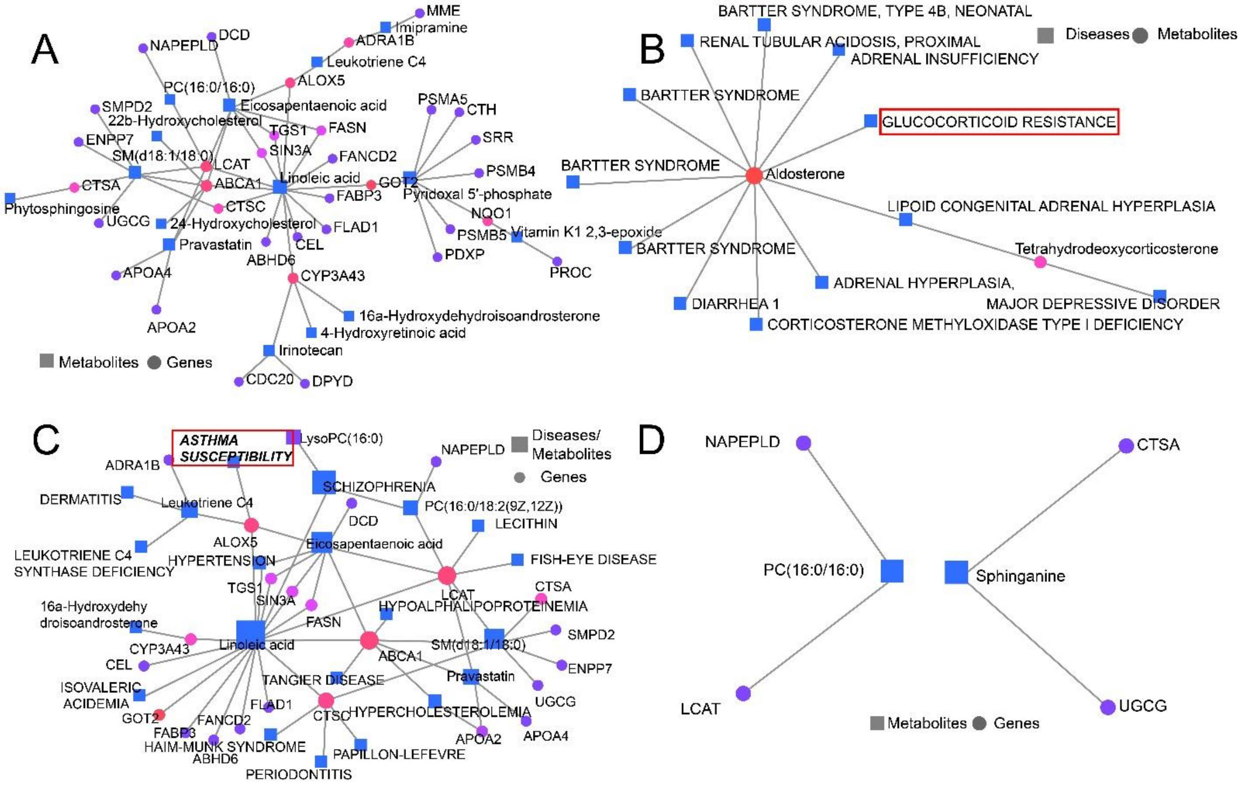

Figure S55. Results of interactions between differential predictive microbial genes and metabolites (**A**), between differential metabolites and the potential diseases (**B**), among differential metabolites, microbial predictive genes and the potential diseases (**C**) and between differential metabolites and microbial predictive genes in BALF (**D**) based on Network Explorer (samples from NEA groups). ABCA1: ATP-binding cassette transporter; ABHD6: α/β-hydrolase domain-6; ADRA1B: adrenergic receptor α-1B; ALOX5: Arachidonate 5-Lipoxygenase; APOA2: Apolipoprotein A2; APOA4: Apo- lipoprotein A4; CDC20: Cell Division Cycle 20; CEL: Carboxyl Ester Lipase; CTH: Cystathionase; CTSA: Cathepsin A; CTSC: Cathepsin C; CYP3A43: Cytochrome P450 Family 3 Subfamily A Member 43; DCD: Dermcidin; DPYD: Dihydropyrimidine Dehydrogenase; ENPP7: Ectonucleotide Pyro-phosphatase /Phosphodiesterase 7; FABP3: Fatty Acid Binding Protein-3; FANCD2: Fanconi anemia complementation group D2; FASN: Fatty Acid Synthase; FLAD1: Flavin Adenine Dinucleoti-de-1; GOT2: Glutamic Oxaloacetic Transaminase-2; LCAT: Lecithin-Cholesterol Acyltransferase; MME: Membrane Metalloendopeptidase; NAPEPLD: N-acyl phosphatidylethanol- amine phospholipase D; NQO1: NAD(P)H Quinone Dehydrogenase 1; PDXP: Pyridoxal Phosphatase; PROC: Protein C, Inactivator Of Coagulation Factors Va and VIIIa; PSMA5: Proteasome Subunit α5; PSMB4: Proteasome Subunit β4; PSMB5: Proteasome Subunit β5; SIN3A: SIN3 Transcription Regulator Family Member A; SMPD2: Sphingomyelin Phosphodiesterase 2; SRR: Serine Racemase; TGS1: Trimethylguanosine Synthase 1; UGCG: UDP-Glucose Ceramide Glucosyltransferase.

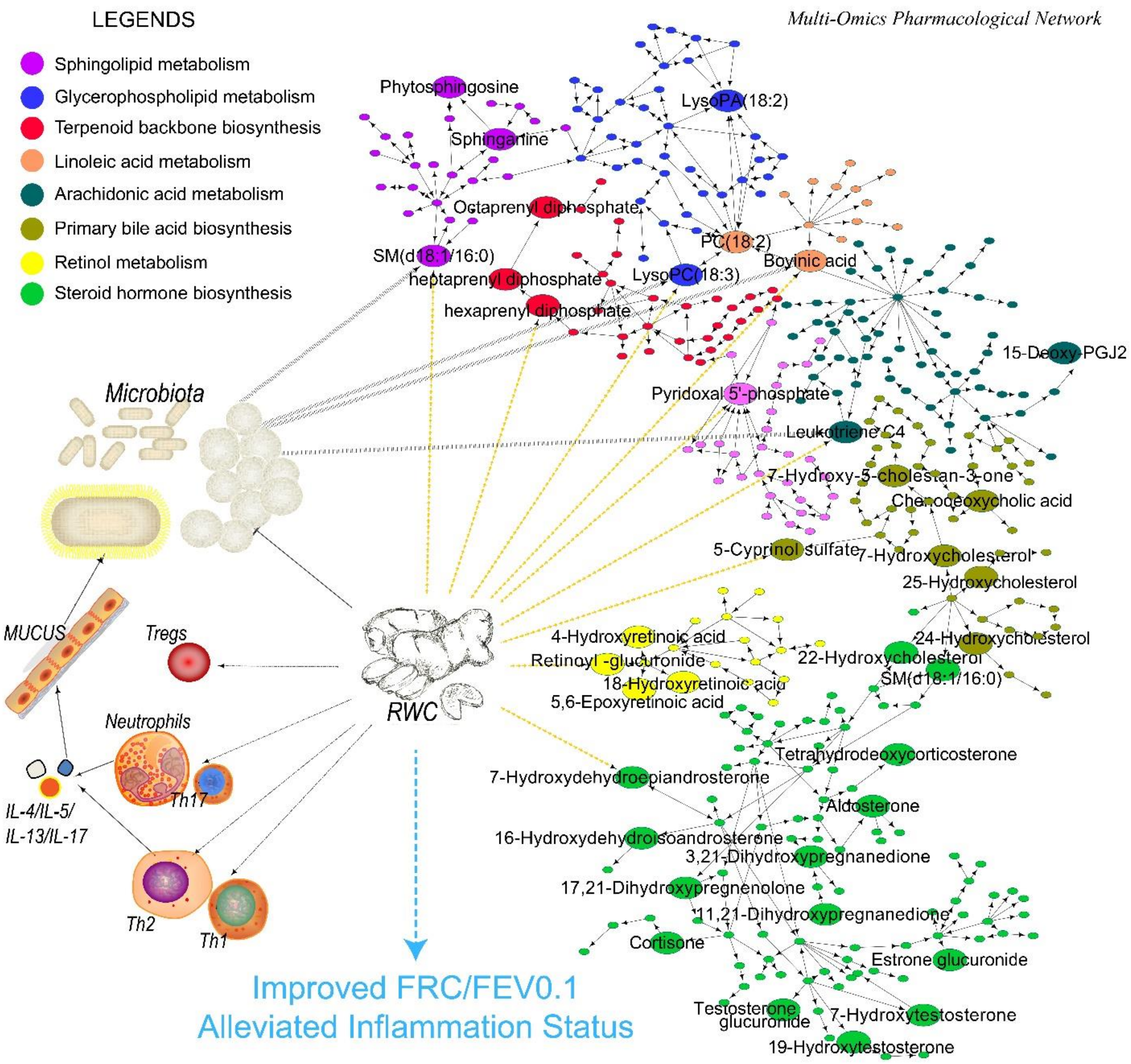

Figure S56. Network on the pathogenesis of asthma and the pharmacological mechanism of RWC based on present series of multi-omics studies.

Table S15. The ARRIVE Guidelines Checklist Animal Research: Reporting In Vivo Experiments

(Attached at the next page)

The ARRIVE Guidelines Checklist

Animal Research: Reporting In Vivo Experiments

Carol Kilkenny[1], William J Browne[2], Innes C Cuthill[3], Michael Emerson[4] and Douglas G Altman[5]

[1]The National Centre for the Replacement, Refinement and Reduction of Animals in Research, London, UK, [2]School of Veterinary Science, University of Bristol, Bristol, UK, [3]School of Biological Sciences, University of Bristol, Bristol, UK, [4]National Heart and Lung Institute, Imperial College London, UK, [5]Centre for Statistics in Medicine, University of Oxford, Oxford, UK.

	ITEM	RECOMMENDATION	Section/ Paragraph
Title	1	Provide as accurate and concise a description of the content of the article as possible.	1
Abstract	2	Provide an accurate summary of the background, research objectives, including details of the species or strain of animal used, key methods, principal findings and conclusions of the study.	1
INTRODUCTION			
Background	3	a. Include sufficient scientific background (including relevant references to previous work) to understand the motivation and context for the study, and explain the experimental approach and rationale.<br>b. Explain how and why the animal species and model being used can address the scientific objectives and, where appropriate, the study's relevance to human biology.	2
Objectives	4	Clearly describe the primary and any secondary objectives of the study, or specific hypotheses being tested.	2
METHODS			
Ethical statement	5	Indicate the nature of the ethical review permissions, relevant licences (e.g. Animal [Scientific Procedures] Act 1986), and national or institutional guidelines for the care and use of animals, that cover the research.	20
Study design	6	For each experiment, give brief details of the study design including:<br>a. The number of experimental and control groups.<br>b. Any steps taken to minimise the effects of subjective bias when allocating animals to treatment (e.g. randomisation procedure) and when assessing results (e.g. if done, describe who was blinded and when).<br>c. The experimental unit (e.g. a single animal, group or cage of animals).<br>A time-line diagram or flow chart can be useful to illustrate how complex study designs were carried out.	20
Experimental procedures	7	For each experiment and each experimental group, including controls, provide precise details of all procedures carried out. For example:<br>a. How (e.g. drug formulation and dose, site and route of administration, anaesthesia and analgesia used [including monitoring], surgical procedure, method of euthanasia). Provide details of any specialist equipment used, including supplier(s).<br>b. When (e.g. time of day).<br>c. Where (e.g. home cage, laboratory, water maze).<br>d. Why (e.g. rationale for choice of specific anaesthetic, route of administration, drug dose used).	20-22
Experimental animals	8	a. Provide details of the animals used, including species, strain, sex, developmental stage (e.g. mean or median age plus age range) and weight (e.g. mean or median weight plus weight range).<br>b. Provide further relevant information such as the source of animals, international strain nomenclature, genetic modification status (e.g. knock-out or transgenic), genotype, health/immune status, drug or test naïve, previous procedures, etc.	20

Housing and husbandry	9	Provide details of:<br>a. Housing (type of facility e.g. specific pathogen free [SPF]; type of cage or housing; bedding material; number of cage companions; tank shape and material etc. for fish).<br>b. Husbandry conditions (e.g. breeding programme, light/dark cycle, temperature, quality of water etc for fish, type of food, access to food and water, environmental enrichment).<br>c. Welfare-related assessments and interventions that were carried out prior to, during, or after the experiment.	20
Sample size	10	a. Specify the total number of animals used in each experiment, and the number of animals in each experimental group.<br>b. Explain how the number of animals was arrived at. Provide details of any sample size calculation used.<br>c. Indicate the number of independent replications of each experiment, if relevant.	20
Allocating animals to experimental groups	11	a. Give full details of how animals were allocated to experimental groups, including randomisation or matching if done.<br>b. Describe the order in which the animals in the different experimental groups were treated and assessed.	20
Experimental outcomes	12	Clearly define the primary and secondary experimental outcomes assessed (e.g. cell death, molecular markers, behavioural changes).	21
Statistical methods	13	a. Provide details of the statistical methods used for each analysis.<br>b. Specify the unit of analysis for each dataset (e.g. single animal, group of animals, single neuron).<br>c. Describe any methods used to assess whether the data met the assumptions of the statistical approach.	22
RESULTS			
Baseline data	14	For each experimental group, report relevant characteristics and health status of animals (e.g. weight, microbiological status, and drug or test naïve) prior to treatment or testing. (This information can often be tabulated).	3
Numbers analysed	15	a. Report the number of animals in each group included in each analysis. Report absolute numbers (e.g. 10/20, not 50%[2]).<br>b. If any animals or data were not included in the analysis, explain why.	3
Outcomes and estimation	16	Report the results for each analysis carried out, with a measure of precision (e.g. standard error or confidence interval).	3-16
Adverse events	17	a. Give details of all important adverse events in each experimental group.<br>b. Describe any modifications to the experimental protocols made to reduce adverse events.	3-16
DISCUSSION			
Interpretation/ scientific implications	18	a. Interpret the results, taking into account the study objectives and hypotheses, current theory and other relevant studies in the literature.<br>b. Comment on the study limitations including any potential sources of bias, any limitations of the animal model, and the imprecision associated with the results[2].<br>c. Describe any implications of your experimental methods or findings for the replacement, refinement or reduction (the 3Rs) of the use of animals in research.	17-19
Generalisability/ translation	19	Comment on whether, and how, the findings of this study are likely to translate to other species or systems, including any relevance to human biology.	17-19
Funding	20	List all funding sources (including grant number) and the role of the funder(s) in the study.	28

References:
1. Kilkenny C, Browne WJ, Cuthill IC, Emerson M, Altman DG (2010) Improving Bioscience Research Reporting: The ARRIVE Guidelines for Reporting Animal Research. *PLoS Biol* 8(6): e1000412. doi:10.1371/journal.pbio.1000412
2. Schulz KF, Altman DG, Moher D, the CONSORT Group (2010) CONSORT 2010 Statement: updated guidelines for reporting parallel group randomised trials. *BMJ* 340:c332.
